# New Linear Precursors of cIDPR Derivatives as Stable Analogs of cADPR: A Potent Second Messenger with Ca^2+^-Modulating Activity Isolated from Sea Urchin Eggs

**DOI:** 10.3390/md17080476

**Published:** 2019-08-17

**Authors:** Stefano D’Errico, Emy Basso, Andrea Patrizia Falanga, Maria Marzano, Tullio Pozzan, Vincenzo Piccialli, Gennaro Piccialli, Giorgia Oliviero, Nicola Borbone

**Affiliations:** 1 Dipartimento di Farmacia, Università degli Studi di Napoli Federico II, via Domenico Montesano 49, Napoli 80131, Italy; 2 ISBE Italy/SYSBIO Centro di System Biology, Università di Milano-Bicocca, piazza delle Scienze 2, Milano 20126, Italy; 3 Consiglio Nazionale delle Ricerche, Dipartimento di Scienze Biomediche, Istituto di Neuroscienze (Sezione di Padova), viale Giuseppe Colombo 3, Padova 35131, Italy; 4 Dipartimento di Scienze Biomediche, Università degli Studi di Padova, via Ugo Bassi 58/b, Padova 35131, Italy; 5 Dipartimento di Medicina Molecolare e Biotecnologie Mediche, Università degli Studi di Napoli Federico II, via Sergio Pansini 5, Napoli 80131, Italy; 6 Istituto Veneto di Medicina Molecolare, via Orus 2, Padova 35129, Italy; 7 Dipartimento di Scienze Chimiche, Università degli Studi di Napoli Federico II, via Cintia, 26, Napoli 80126, Italy

**Keywords:** cADPR, ryanodine receptors, neuroblastoma, caffeine, calcium mobilization, phosphorylation, C2C12 cells, IP3

## Abstract

Herein, we report on the synthesis of a small set of linear precursors of an inosine analogue of cyclic ADP-ribose (cADPR), a second messenger involved in Ca^2+^ mobilization from ryanodine receptor stores firstly isolated from sea urchin eggs extracts. The synthesized compounds were obtained starting from inosine and are characterized by an N1-alkyl chain replacing the “northern” ribose and a phosphate group attached at the end of the N1-alkyl chain and/or 5′-sugar positions. Preliminary Ca^2+^ mobilization assays, performed on differentiated C2C12 cells, are reported as well.

## 1. Introduction

**Figure 1:**
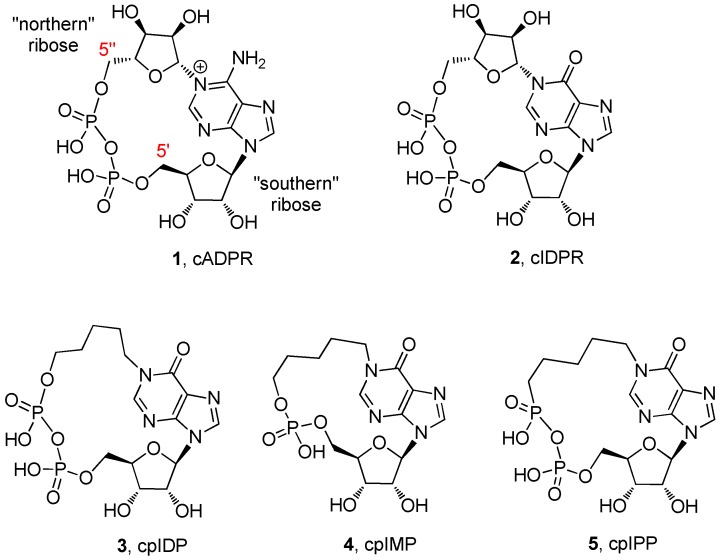
The structures of cyclic adenosine diphosphate ribose (cADRP; **1**) and some of its analogues (**2**–**5**).

Ca^2+^-mediated signaling is the major secondary messenger involved in several essential cell functions. Ca^2+^ ions are generally located in the endoplasmic reticulum (ER) and mitochondria until an extracellular stimulus causes their release into the cytosol [1,2]. This mobilization may lead to a great number of physiological effects such as gene regulation [3], cell proliferation, fertilization [4], muscle contraction [5], and neurotransmitter secretion [6]. Cyclic adenosine diphosphate ribose (cADPR, **1**, Figure 1) is an endogenous cyclic nucleotide that was firstly isolated from sea urchin egg extracts [7], later found also in mammalian cells, such as pancreatic β-cells [8], T-lymphocytes [9], smooth and cardiac muscle cells [10], and cerebellar neurons [11]. cADPR, together with inositol 1,4,5-triphosphate (IP3) and nicotinic acid adenine dinucleotide phosphate (NAADP), is a second messenger involved in cellular Ca^2+^ mobilization [12,13]. It interacts with ryanodine receptors through a mechanism which is still unclear, causing the Ca^2+^ release from intracellular stores [14]. The 18 membered cyclic structure comes from the catalytic activity of the enzyme ADP-ribosyl cyclase, which in mammalian cells is the multifunctional transmembrane glycoprotein CD38 [15,16]. The latter is able also to catalyze the inverse hydrolytic reaction that produces adenosine diphosphate ribose (ADPR) [17]. The physiological instability of cADPR at the N1-glycosidic bond [17], together with its low ability to cross membranes, likely for the presence of the negative charge at the pyrophosphate moiety [18], have prompted some researchers to develop semi-synthetic and/or synthetic methodologies to obtain novel non-hydrolysable and cell permeant analogues [17,19,20,21,22,23].

Specific structure–activity relationships on different cell lines have been tuned [24,25], featuring modifications mainly involving the “northern” and/or the “southern” ribose. In many cases, the replacement of the northern ribose with ether/alkyl chains was revealed to be the most fruitful modifications for the obtainment of cell permeant analogues, likely by virtue of their reduced molecular polarity [22]. The finding that the isosteric replacement of the adenine base with hypoxanthine generated a more stable analogue (cyclic inosine diphosphate ribose, cIDPR, **2**, Figure 1) with intact Ca^2+^-mobilizing properties [26], laid the foundations for a more detailed comprehension of the molecular mechanism of action of cADPR, that also took advantage from studies of the biological properties of novel cIDPR analogues [27,28]. However, the poor knowledge of the receptorial cADPR binding pocket [29] makes difficult the rational design of analogues; moreover, the analogues can behave differently depending on the cell line used.

Over the last years, our research group synthesized several cIDPR analogues [30,31,32,33,34,35,36,37]. In particular, the pyrophosphate (cpIDP, **3**, Figure 1) [30] and the monophosphate (cpIMP, **4**, Figure 1) [36] derivatives with a pentyl chain replacing the “northern” ribose showed interesting Ca^2+^-releasing activities in PC12 cells differentiated in neurons with the use of nerve growth factor (NGF) [18]. Meanwhile, the pyrophosphate derivative with a butyl chain in the place of the “northern” ribose was inactive on the same cell line [37]. In addition, we synthesized the derivative **5** (Figure 1), bearing a phosphono-phosphate anhydride in the place of the pyrophosphate, with the aim of better exploring the role of the pyrophosphate in Ca^2+^-mobilizing properties of cADPR/cIDPR analogues [37]. Unfortunately, the compound **5** also failed to modify the [Ca^2+^]_i_ in PC12 cells. These biological results allowed us to hypothesize two preliminary structure–activity relationships: (1) one phosphate group appears to be sufficient for the Ca^2+^-releasing activity in PC12 neuronal cells; (2) the N1-pentyl chain is likely essential for a better conformational accommodation within the putative binding pocket of the intracellular receptor.

**Scheme 1:**
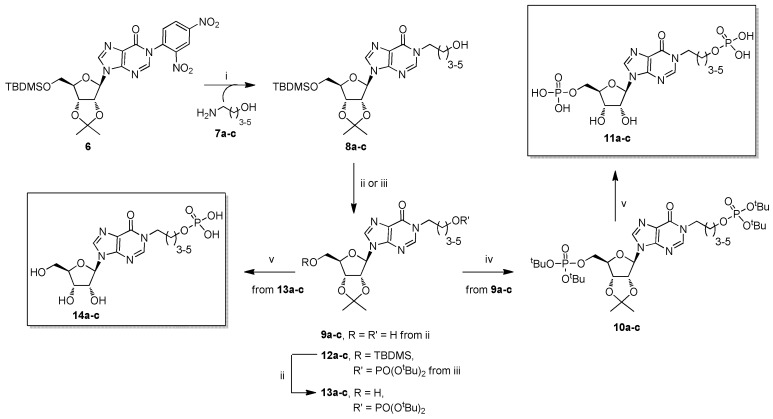
Reagents and conditions. (**i**) **7a**–**c**, *N*,*N*-dimethylformamide (DMF), 50 °C, 16 h; (**ii**) tetrabutylammonium fluoride (TBAF), tetrahydrofuran (THF), r.t., 1 h; (**iii**) (a) di-*tert*-butyl *N*,*N*-diisopropylphosphoramidite ((^t^BuO)_2_PN(^i^Pr)_2_), 1-*H*-tetrazole, THF, r.t., 6 h, (b) *tert*-butyl hydroperoxide (^t^BuOOH), THF, r.t., 1 h; (**iv**) 50% trifluoroacetic acid (TFA) in H_2_O, r.t., 4 h.

**Scheme 2:**
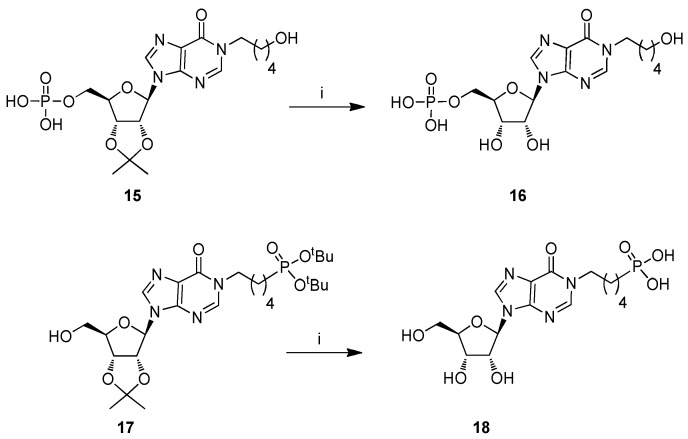
Reagents and conditions. (**i**) 50% TFA in H_2_O, r.t., 4 h.

The transient receptor potential melastatin 2 (TRPM2) is a non-selective cation channel embedded in plasma membrane and/or lysosomal compartments, that is involved in the response to oxidative stress and inflammation [38,39,40]. It has been found that ADPR, binding the zebrafish melastatin homology domain 1/2 (MHR1/2) or the human nudix hydrolase 9 homology domain (NUDT9H) of TRPM2, can open the channel favoring the Ca^2+^ ions entry [41]. These findings, together with the discovery that N1-ribose 5′’-monophosphate inosines could act as inhibitors of cADPR hydrolysis [42], are challenging and open the way to the design and synthesis of novel modulators/inhibitors structurally related to cADPR/cIDPR. The latter can be obtained more easily in respect to the cyclic counterparts, thanks to the lack of the last limiting macrocyclization step. In this frame, here, we report on the synthesis and the preliminary evaluation of Ca^2+^-mobilizing properties of a small set of N1-*ω*-alkyl phosphate 5′-phosphate and N1-*ω*-hydroxyalkyl 5′-phosphate inosines (**11** and **14**, Scheme 1, respectively) obtained by varying the length of the N1 purine alkyl chain from four to six carbon atoms. We also synthesized and tested the two monophosphate derivatives **16** and **18** (Scheme 2). Compound **16** is a useful intermediate for the obtainment of the biologically active cpIDP **4**, whereas compound **18** can be used for the synthesis of cpIPP **5**. These compounds enrich the box of linear precursors of cIDPR analogues, that, until now, have been mainly employed as synthetic intermediates.

## 2. Results and Discussion

### 2.1. Chemistry

Compounds **11** and **14** were synthesized starting from the versatile building block **6** (Scheme 1), readily obtained from inosine [36].

The strong 2,4-dinitrophenyl electron-withdrawing group makes the C2 purine atom very reactive towards amino-alcohols **7a**–**c**, affording high yields (70–84%) of N1 *ω*-hydroxyalkyl inosines (**8a-c**) [43,44,45,46], through a mechanism that we have studied in detail [47,48]. In particular, the nucleophilic addition of the amino group to the C2 purine atom leads to the pyrimidine ring opening; then, the same amino group attacks the imidazo 4-carboxamide functionality, reclosing the pyrimidine ring with the 2,4-dinitroaniline displacement. As a next step of the synthesis, the ribose *tert*-butyldimethylsilyl (TBDMS) group was quantitatively removed from derivatives **8a**–**c** with tetrabutylammonium fluoride (TBAF), yielding the compounds **9a**–**c**. Then, the phosphorylation of the pendant hydroxy functionalities in **9a**–**c** was studied. Our research group [37] and others [49,50] demonstrated that the mono-phosphorylation of a nucleoside could be performed in mild and good yielding conditions, exploiting the phosphoramidite chemistry. When compounds **9a**–**c** were reacted with di-*tert*-butyl *N*,*N*-diisopropylphosphoramidite ((^t^BuO)_2_PN(^i^Pr)_2_) and 1-*H*-tetrazole, followed by *tert*-butyl hydroperoxide (^t^BuOOH) oxidation of the phosphite to phosphate, compounds **10a**–**c** (58–62%) were obtained. Recent studies report on the use of the soluble 5-phenyl-1-*H*-tetrazole as activator for the phosphitylation step [49,50]; but, in our hands, it did not furnish satisfying yields of the phosphate-protected nucleotides. The deprotection of both phosphate and 2′,3′-*O*-ribose protecting groups at the final stage of the synthesis was carried out using an aqueous solution of trifluoroacetic acid (TFA). This process proceeded smoothly and could be easily monitored by TLC, allowing the recovery of quantitative yields of the nucleotides **11a**–**c**. Conversely, to obtain compounds **14a**–**c**, intermediates **8a**–**c** were phosphorylated to give the derivatives **12a**–**c** (57–60% over two steps). Desilylation with TBAF of **12a**–**c** to afford **13a**–**c**, followed by the usual acidic treatment, yielded compounds **14a**–**c** almost quantitatively. For the synthesis of nucleotide **16** (Scheme 2), we started from the intermediate **15**, useful for the synthesis of cpIMP **4** [36]. The acidic treatment on **15** afforded directly the target **16** (99% yield). As verified by reverse-phase HPLC [51], the derivatives **11a**–**c**, **14a**–**c**, and **16** were pure. Differently, the aqueous TFA treatment of the protected phosphonate **17** (Scheme 2), the precursor of cpIPP **5** [37], led to a complex mixture from which compound **18** was isolated in 50% yield. This low yield could be, therefore, attributable to the lower acidic resistance of the phosphonate compared with the phosphate moiety.

To evaluate the stability of the synthesized compounds, **11a**–**c**, **14a**–**c**, **16**, and **18** were dissolved in a phosphate buffered solution (pH = 7.4) and stored at room temperature. The comparison of the HPLC profiles obtained by injecting samples after 24, 48, and 72 h showed no appreciable decomposition of all tested compounds (data not shown).

### 2.2. Biology

**Figure 2:**
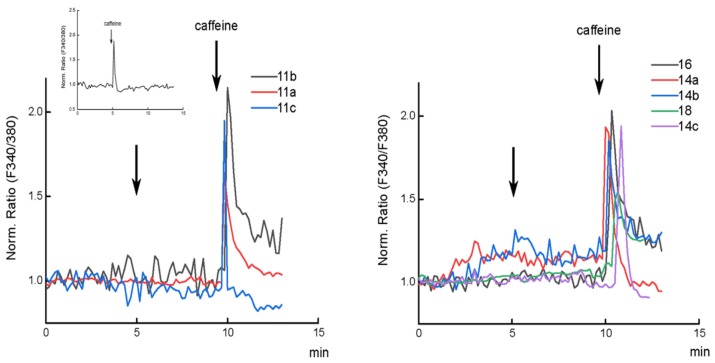
Effect of compounds **11a**–**c** (left), **14a**–**c**, **16**, and **18** (right) on cytosolic [Ca^2+^]. Representative traces of normalized Fura-2 fluorescence ratio for differentiated C2C12 cells. 1 µM of each compound was added, then 10 mM caffeine was added 5 min after the addition of each compound as a control for Ca^2+^ release from ryanodine receptors. The inset in the left panel shows 10 mM caffeine addition to untreated cells.

The compounds **11a**–**c**, **14a**–**c**, **16**, and **18** were tested to evaluate their ability to mobilize Ca^2+^ ions from ryanodine receptor expressing stores of C2C12 cells (Figure 2) [52]. At first, to verify that the system worked, differentiated C2C12 cells were loaded with Fura-2 and treated with caffeine. An increase of cytosolic [Ca^2+^] was observed, as verified by the increase of the 340/380 fluorescence ratio of cytosolic Fura-2. The molecules were tested, probing concentrations from 0.1 up to 20 µM, though no effect was observed on the cytosolic [Ca^2+^].

Compounds **11b** and **16,** at the concentration of 1 µM, appear to enhance the effect of caffeine on the Ca^2+^ release from the stores (see Supplementary Materials, Figure S3). This effect could be ascribed to a stimulus of Ca^2+^ release from the ER, increasing the Ca^2+^ sensitivity of ryanodine receptors [53,54]. The Ca^2+^-induced Ca^2+^ release (CICR) mediated by ryanodine receptors is a very important mechanism for the amplification of the Ca^2+^ signal in several mammalian cells, such as neurons, astrocytes, and pancreatic *β*-cells [55]. It could be not excluded that the amplification of the Ca^2+^ signal depends on CICR through other channels located either in the plasma or in intracellular store membranes, as a consequence of reticulum emptying.

## 3. Materials and Methods

### 3.1. General Experimental Procedures

All the reagents and solvents for the chemical syntheses were obtained from commercial sources and used without further purification. The ^1^H- and ^13^C-NMR experiments were performed using the Varian Mercury Plus 400 MHz and ^UNITY^INOVA 500 MHz spectrometers and CDCl_3_, CD_3_OD, C_6_D_6_, and D_2_O as solvents. The NMR chemical shifts are reported in parts per million (*δ*) relative to residual solvents signals: CHCl_3_ 7.27, CD_2_HOD 3.31, C_6_D_5_H 7.15, HOD 4.80 for ^1^H-NMR and CDCl_3_ 77.0, CD_3_OD 49.0, C_6_D_6_ 128.1 for ^13^C NMR. The ^1^H-decoupled ^31^P-NMR experiments were carried out on a Varian ^UNITY^INOVA 500 MHz instrument in CDCl_3_, D_2_O, and C_6_D_6_ solvents using 85% H_3_PO_4_ as an external standard (0 ppm). The ^1^H-NMR chemical shifts were assigned through 2D NMR experiments. Electrospray ionization (ESI) mass spectra were recorded on an AB SCIEX QTRAP 4000 spectrometer. All NMR spectra were processed using the iNMR software package (http://www.inmr.net; release 6.2.1). Column chromatography was carried out on silica gel-60 with particle size of 0.063–0.200 mm (Merck, Darmstatd, Germany). Analytical TLC analyses were performed using F254 silica gel plates of 0.2 mm thick (Merck, Darmstatd, Germany). The TLC spots were detected under UV light (254 nm). High-performance liquid chromatography (HPLC) was performed on a Jasco UP-2075 Plus pump equipped with a Jasco UV-2075 Plus UV detector using a 4.8 × 150 mm C-18 reverse-phase column (particle size 5 µm) eluted with a linear gradient of CH_3_CN in 0.1 M triethylammonium bicarbonate (TEAB) buffer (from 0 to 100% in 60 min, flow 2.0 mL/min). The C2C12 cell line was cultured in DMEM medium (Merck, Darmstatd, Germany) supplemented with 10% fetal bovine serum, 2 mM L-glutamine, and incubated at 37 °C in a controlled atmosphere with 5% CO_2_. For Ca^2+^ measurements, the cells were plated on 24 mm glass coverslip treated with 2% gelatin and differentiated by culturing in DMEM supplemented with 5% horse serum and 2 mM L-glutamine for 5–7 days. Statistical analyses (Mann–Whitney non-parametric test) were performed on Origin 2019b software package (www.originlab.com).

### 3.2. Chemistry

#### 3.2.1. General Procedure for the Preparation of Compounds **8a**–**c**

In a typical experiment, compound **6** [36] (0.20 g, 0.34 mmol) was dissolved in DMF (1.0 mL) and treated with the amino alcohol **7a** (0.16 mL, 1.7 mmol). The reaction was stirred at 50 °C for 16 h and then evaporated under reduced pressure (TLC monitoring: CHCl_3_/MeOH; 95:5). The crude containing compound **8a** was purified through a column of silica gel eluted with increasing amounts of MeOH in CHCl_3_ (up to 2%). The fractions containing the product were collected and evaporated to afford the pure **8a**.

**8a:** Oil (70% yield). ^1^H NMR (400 MHz, CDCl_3_) δ 8.03 (s, 2H, 2-H and 8-H), 6.10 (d, *J* = 2.3 Hz, 1H, 1’-H), 5.07 (dd, *J* = 5.9, 2.4 Hz, 1H, 2’-H), 4.89 (dd, *J* = 5.9, 1.9 Hz, 1H, 3’-H), 4.44–4.40 (m, 1H, 4’-H), 4.18–4.04 (m, 2H, CH_2_N), 3.86 (dd, *J* = 11.3, 3.2 Hz, 1H, 5’-H_a_), 3.77 (dd, *J* = 11.4, 3.5 Hz, 1H, 5’-H_b_), 3.69 (t, *J* = 6.1 Hz, 2H, CH_2_O), 2.63 (bs, 1H, OH, exchange with D_2_O), 1.91–1.84 (m, 2H, CH_2_), 1.65–1.56 (complex signal, 5H, CH_3_ and CH_2_), 1.38 (s, 3H, CH_3_), 0.83 (s, 9H, ^t^Bu), 0.027 (s, 3H, SiCH_3_), 0.01 (s, 3H, SiCH_3_). ^13^C NMR (100 MHz, CDCl_3_) δ 156.6, 147.3, 147.0, 138.4, 124.8, 114.2, 91.4, 87.1, 85.4, 81.3, 63.5, 61.8, 46.7, 29.1, 27.2, 26.5, 25.8, 25.3, 18.3, −5.40, −5.60. ESI-MS *m/z* 495 ([M + H]^+^, C_23_H_39_N_4_O_6_Si, requires 495).

**8b:** Oil (85% yield).^1^H NMR (400 MHz, CDCl_3_) δ 8.02 (s, 1H, 2-H), 7.98 (s, 1H, 8-H), 6.09 (d, *J* = 2.0 Hz, 1H, 1’-H), 5.06 (dd, *J* = 5.6, 2.4 Hz, 1H, 2’-H), 4.91–4.85 (m, 1H, 3’-H), 4.43–4.38 (m, 1H, 4’-H), 4.12–3.99 (m, 2H, CH_2_N), 3.84 (dd, *J* = 11.2, 2.7 Hz, 1H, 5’-H_a_), 3.76 (dd, *J* = 11.3, 3.1 Hz, 1H, 5’-H_b_), 3.61 (t, *J* = 5.9 Hz, 2H, CH_2_O), 2.52 (bs, 1H, OH, exchange with D_2_O), 1.84–1.73 (m, 2H, CH_2_), 1.64–1.52 (complex signal, 5H, CH_3_ and CH_2_), 1.46–1.34 (complex signal, 5H, CH_3_ and CH_2_), 0.82 (s, 9H, ^t^Bu), 0.02 (s, 3H, SiCH_3_), 0.00 (s, 3H, SiCH_3_). ^13^C NMR (100 MHz, CDCl_3_) δ 156.5, 147.2, 146.9, 138.3, 124.8, 114.1, 91.4, 87.0, 85.4, 81.3, 63.5, 62.1, 46.9, 32.0, 29.6, 27.2, 25.8, 25.3, 22.7, 18.3, −5.40, −5.60. ESI-MS *m/z* 509 ([M + H]^+^, C_24_H_41_N_4_O_6_Si, requires 509).

**8c:** Oil (84% yield).^1^H NMR (400 MHz, CDCl_3_) δ 8.02 (s, 1H, 2-H), 7.96 (s, 1H, 8-H), 6.10 (d, *J* = 2.4 Hz, 1H, 1’-H), 5.06 (dd, *J* = 6.0, 2.5 Hz, 1H, 2’-H), 4.88 (dd, *J* = 5.9, 2.1 Hz, 1H, 3’-H), 4.45–4.39 (m, 1H, 4’-H), 4.12–3.96 (m, 2H, CH_2_N), 3.85 (dd, *J* = 11.3, 3.2 Hz, 1H, 5’-H_a_), 3.76 (dd, *J* = 11.3, 3.5 Hz, 1H, 5’-H_b_), 3.61 (t, *J* = 6.5 Hz, 2H, CH_2_O), 2.01 (bs, 1H, OH, exchange with D_2_O), 1.82–1.71 (m, 2H, CH_2_), 1.61 (s, 3H, CH_3_), 1.58–1.49 (m, 2H, CH_2_), 1.41–1.34 (complex signal, 7H, CH_3_ and 2 × CH_2_), 0.82 (s, 9H, ^t^Bu), 0.02 (s, 3H, SiCH_3_), 0.01 (s, 3H, SiCH_3_). ^13^C NMR (100 MHz, CDCl_3_) δ 156.5, 147.2, 146.9, 138.3, 124.9, 114.2, 91.4, 87.0, 85.4, 81.3, 63.5, 62.5, 46.8, 32.4, 29.8, 27.2, 26.2, 25.3, 25.2, 18.3, −5.40, −5.50. ESI-MS *m/z* 523 ([M + H]^+^, C_25_H_43_N_4_O_6_Si, requires 523).

#### 3.2.2. General Procedure for the Preparation of Compounds **9a**–**c**

In a typical experiment, to a solution of compound **8a** (0.12 g, 0.24 mmol) in dry THF (1.0 mL), TBAF (0.29 mL of a 1.0 M solution in dry THF, 0.29 mmol) was added dropwise. The reaction mixture was stirred at room temperature for 1 h (TLC monitoring: AcOEt/MeOH; 9:1) and then evaporated under reduced pressure. The residue was purified over a silica gel column eluted with increasing amounts of MeOH in AcOEt (up to 5%) to afford the pure **9a**.

**9a:** Oil (99% yield). ^1^H NMR (400 MHz, CD_3_OD) δ 8.32 (s, 1H, 2-H), 8.30 (s, 1H, 8-H), 6.15 (d, *J* = 3.0 Hz, 1H, 1’-H), 5.25 (dd, *J* = 6.1, 3.0 Hz, 1H, 2’-H), 5.00 (dd, *J* = 6.1, 2.5 Hz, 1H, 3’-H), 4.36–4.30 (m, 1H, 4’-H), 4.12 (t, *J* = 7.3 Hz, 2H, CH_2_N), 3.75 (dd, *J* = 11.9, 2.8 Hz, 1H, 5’-H_a_), 3.70 (dd, *J* = 11.9, 4.4 Hz, 1H, 5’-H_b_), 3.59 (t, *J* = 6.4 Hz, 2H, CH_2_O), 1.89–1.78 (m, 2H, CH_2_), 1.63–1.53 (complex signal, 5H, CH_3_ and CH_2_), 1.36 (s, 3H, CH_3_). ^13^C NMR (100 MHz, CD_3_OD) δ 158.1, 149.7, 148.5, 141.3, 125.2, 115.2, 92.3, 88.4, 85.8, 82.8, 63.3, 62.3, 47.8, 30.4, 27.5, 27.3, 25.5. ESI-MS *m/z* 381 ([M + H]^+^, C_17_H_25_N_4_O_6_, requires 381).

**9b:** Oil (99% yield). ^1^H NMR (400 MHz, CD_3_OD) δ 8.32 (s, 1H, 2-H), 8.31 (s, 1H, 8-H), 6.15 (d, *J* = 3.0 Hz, 1H, 1’-H), 5.24 (dd, *J* = 6.0, 3.0 Hz, 1H, 2’-H), 4.99 (dd, *J* = 6.0, 2.5 Hz, 1H, 3’-H), 4.37–4.31 (m, 1H, 4’-H), 4.09 (t, *J* = 7.3 Hz, 2H, CH_2_N), 3.72 (dd, *J* = 12.0, 3.8 Hz, 1H, 5’-H_a_), 3.70 (dd, *J* = 12.0, 4.4 Hz, 1H, 5’-H_b_), 3.55 (t, *J* = 6.4 Hz, 2H, CH_2_O), 1.84–1.74 (m, 2H, CH_2_), 1.62–1.53 (complex signal, 5H, CH_3_ and CH_2_), 1.48–1.38 (m, 2H, CH_2_), 1.36 (s, 3H, CH_3_). ^13^C NMR (100 MHz, CD_3_OD) δ 158.1, 149.6, 148.5, 141.2, 125.3, 115.2, 92.3, 88.4, 85.8, 82.8, 63.3, 62.5, 47.9, 33.1, 30.4, 27.5, 25.5, 23.9. ESI-MS *m/z* 395 ([M + H]^+^, C_18_H_27_N_4_O_6_, requires 395).

**9c:** Oil (99% yield). ^1^H NMR (400 MHz, CD_3_OD) δ 8.31 (s, 1H, 2-H), 8.30 (s, 1H, 8-H), 6.15 (d, *J* = 2.9 Hz, 1H, 1’-H), 5.25 (dd, *J* = 6.0, 3.0 Hz, 1H, 2’-H), 5.00 (dd, *J* = 6.0, 2.5 Hz, 1H, 3’-H), 4.37–4.30 (m, 1H, 4’-H), 4.09 (t, *J* = 7.3 Hz, 2H, CH_2_N), 3.75 (dd, *J* = 11.9, 3.8 Hz, 1H, 5’-H_a_), 3.70 (dd, *J* = 11.9, 4.4 Hz, 1H, 5’-H_b_), 3.53 (t, *J* = 6.5 Hz, 2H, CH_2_O), 1.83–1.72 (m, 2H, CH_2_), 1.58 (s, 3H, CH_3_), 1.56–1.49 (m, 2H, CH_2_), 1.45–1.38 (m, 4H, 2 × CH_2_), 1.36 (s, 3H, CH_3_). ^13^C NMR (100 MHz, CD_3_OD) δ 158.1, 149.6, 148.5, 141.2, 125.2, 115.2, 92.3, 88.4, 85.8, 82.8, 63.3, 62.7, 47.9, 33.4, 30.7, 27.5, 27.3, 26.5, 25.5. ESI-MS *m/z* 409 ([M + H]^+^, C_19_H_29_N_4_O_6_, requires 409).

#### 3.2.3. General Procedure for the Preparation of Compounds **10a**–**c**

In a typical experiment, to a solution of compound **9a** (0.050 g, 0.13 mmol) in dry THF (2.0 mL), 1-*H*-tetrazole (0.065 g, 0.91 mmol) and then ^i^Pr_2_NP(O^t^Bu)_2_ (0.57 mL, 1.8 mmol) were added under nitrogen atmosphere. The reaction mixture was stirred at room temperature for 6 h (TLC monitoring: AcOEt/MeOH, 9:1) and then ^t^BuOOH (0.24 mL of a solution 5.5 M in decane, 1.3 mmol) was added at room temperature. After 1 h (TLC monitoring: AcOEt/MeOH, 9:1), the reaction mixture was evaporated under reduced pressure, diluted with AcOEt (10 mL), and washed with brine (10 mL). The organic layer was dried over anhydrous Na_2_SO_4_, filtered, and evaporated under reduced pressure. The residue was purified over a silica gel column eluted with increasing amounts of MeOH in AcOEt (up to 5%) to afford pure **10a**.

**10a:** Oil (60% yield over two steps). ^1^H NMR (400 MHz, CDCl_3_) δ 8.02 (s, 1H, 2-H), 7.98 (s, 1H, 8-H), 6.11 (d, *J* = 2.7 Hz, 1H, 1’-H), 5.19 (dd, *J* = 6.1, 2.8 Hz, 1H, 2’-H), 5.02 (dd, *J* = 6.1, 2.6 Hz, 1H, 3’-H), 4.50–4.46 (m, 1H, 4’-H), 4.20–4.05 (complex signal, 4H, CH_2_OP, 5’-H_a,b_), 4.03–3.95 (m, 2H, CH_2_N), 1.95–1.85 (m, 2H, CH_2_), 1.78–1.68 (m, 2H, CH_2_), 1.63 (s, 3H, CH_3_), 1.47 (s, 27H, 3 × O^t^Bu), 1.44 (s, 9H, O^t^Bu), 1.38 (s, 3H, CH_3_). ^13^C NMR (100 MHz, CDCl_3_) δ 156.4, 147.3, 147.0, 138.5, 125.0, 114.6, 90.6, 84.8 (d, *J* = 8.3 Hz), 84.6, 83.1 (d, *J* = 7.4 Hz), 83.0 (d, *J* = 7.2 Hz), 82.3 (d, *J* = 7.3 Hz), 81.3, 66.0 (d, *J* = 6.1 Hz), 65.9 (d, *J* = 6.5 Hz), 46.3, 29.9, 29.8, 29.7, 27.2, 26.2, 25.3. ^31^P NMR (202 MHz, CDCl_3_) δ −9.80 (s). ESI-MS *m/z* 765 ([M + H]^+^, C_33_H_59_N_4_O_12_P_2_, requires 765).

**10b:** Oil (58% yield over two steps). ^1^H NMR (400 MHz, CDCl_3_) δ 7.97 (s, 1H, 2-H), 7.96 (s, 1H, 8-H), 6.09 (d, *J* = 2.8 Hz, 1H, 1’-H), 5.18 (dd, *J* = 6.2, 2.9 Hz, 1H, 2’-H), 5.01 (dd, *J* = 6.2, 2.8 Hz, 1H, 3’-H), 4.49–4.43 (m, 1H, 4’-H), 4.18–4.10 (m, 2H, 5’-H_a,b_), 4.08–3.97 (m, 2H, CH_2_OP), 3.96–3.89 (m, 2H, CH_2_N), 1.85–1.75 (m, 2H, CH_2_), 1.73–1.65 (m, 2H, CH_2_), 1.61 (s, 3H, CH_3_), 1.45 (s, 29H, 3 × O^t^Bu and CH_2_), 1.42 (s, 9H, O^t^Bu), 1.36 (s, 3H, CH_3_). ^13^C NMR (100 MHz, CDCl_3_) δ 156.4, 147.2, 147.0, 138.5, 125.0, 114.6, 90.6, 84.8 (d, *J* = 8.4), 84.6, 83.1 (d, *J* = 7.4), 83.0 (d, *J* = 7.3), 82.1 (d, *J* = 7.3), 81.3, 66.3 (d, *J* = 6.4), 66.0 (d, *J* = 5.5), 46.8, 29.3, 29.8, 29.7, 29.4, 27.2, 25.3, 22.8. ^31^P NMR (202 MHz, CDCl_3_) δ –9.70 (s), −9.80 (s). ESI-MS *m/z* 779 ([M + H]^+^, C_34_H_61_N_4_O_12_P_2_, requires 779).

**10c:** Oil (62% yield over two steps). ^1^H NMR (400 MHz, CDCl_3_) δ 7.98 (s, 1H, 2-H), 7.97 (s, 1H, 8-H), 6.11 (d, *J* = 2.8 Hz, 1H, 1’-H), 5.20 (dd, *J* = 6.2, 2.8 Hz, 1H, 2’-H), 5.02 (dd, *J* = 6.2, 2.7 Hz, 1H, 3’-H), 4.50–4.46 (m, 1H, 4’-H), 4.19–4.10 (m, 2H, 5’-H_a,b_), 4.08–4.00 (m, 2H, CH_2_OP), 3.97–3.90 (m, 2H, CH_2_N), 1.83–1.74 (m, 2H, CH_2_), 1.70–1.61 (complex signal, 5H, CH_2_ and CH_3_), 1.47 (s, 31H, 3 × O^t^Bu and 2 × CH_2_), 1.43 (s, 9H, O^t^Bu), 1.38 (s, 3H, CH_3_). ^13^C NMR (100 MHz, CDCl_3_) δ 156.4, 147.2, 147.0, 138.5, 125.0, 114.6, 90.6, 84.8 (d, *J* = 8.4), 84.6, 83.1 (d, *J* = 7.4), 83.0 (d, *J* = 7.3), 82.0 (d, *J* = 7.4), 81.3, 66.5 (d, *J* = 6.4), 66.0 (d, *J* = 5.6), 46.9, 30.0, 29.9, 29.8, 29.7, 27.2, 26.2, 25.3, 25.2. ^31^P NMR (202 MHz, CDCl_3_) δ −9.70 (s), −9.80 (s). ESI-MS *m/z* 793 ([M + H]^+^, C_35_H_63_N_4_O_12_P_2_, requires 793).

#### 3.2.4. General Procedure for the Preparation of Compounds **11a**–**c**

In a typical experiment, compound **10a** (0.020 g, 0.026 mmol) was dissolved in a 1:1 (v/v) solution of TFA in H_2_O (1.0 mL) at 0 °C [56]. The reaction mixture was warmed to room temperature, stirred for 4 h (TLC monitoring: *^i^*PrOH/NH_3_(aq)/H_2_O, 6:3:1) and then lyophilized.

**11a**: White foam (99% yield). ^1^H NMR (500 MHz, D_2_O) δ 9.08 (s, 1H, 2-H), 8.54 (s, 1H, 8-H), 6.23 (d, *J* = 3.8 Hz, 1H, 1’-H), 4.76–4.72 (m, 1H, 2’-H), 4.52–4.47 (m, 1H, 3’-H), 4.45–4.40 (m, 1H, 4’-H), 4.30–4.24 (m, 1H, 5’-H_a_), 4.23–4.13 (complex signal, 3H, CH_2_OP, 5’-H_b_), 4.01–3.95 (m, 2H, CH_2_N), 1.96–1.86 (m, 2H, CH_2_), 1.77–1.68 (m, 2H, CH_2_). ^13^C NMR (100 MHz, D_2_O) δ 155.1, 150.9, 146.3, 138.5, 118.2, 89.6, 83.9 (d, *J* = 8.5 Hz), 74.7, 69.4, 65.9 (d, *J* = 5.3 Hz), 63.9 (d, *J* = 4.5 Hz), 47.2, 26.5 (d, *J* = 6.8 Hz), 25.0. ^31^P NMR (202 MHz, D_2_O) δ 0.00 (s), −0.08 (s). ESI-MS *m/z* 499 ([M − H]^–^, C_14_H_21_N_4_O_12_P_2_, requires 499).

**11b**: White foam (99% yield). ^1^H NMR (500 MHz, D_2_O) δ 9.06 (s, 1H, 2-H), 8.47 (s, 1H, 8-H), 6.17 (d, *J* = 3.7 Hz, 1H, 1’-H), 4.68–4.65 (m, 1H, 2’-H), 5.51–4.47 (m, 1H, 3’-H), 4.37–4.33 (m, 1H, 4’-H), 4.21 (ddd, *J* = 11.9, 4.4, 2.4 Hz, 1H, 5’-H_a_), 4.13–4.07 (complex signal, 3H, CH_2_OP, 5’-H_b_), 3.91–3.86 (m, 2H, CH_2_N), 1.80–1.72 (m, 2H, CH_2_), 1.67–1.58 (m, 2H, CH_2_), 1.42–1.33 (m, 2H, CH_2_). ^13^C NMR (100 MHz, D_2_O) δ 154.9, 150.9, 146.1, 138.3, 117.9, 89.5, 83.8 (d, *J* = 8.6 Hz), 74.6, 69.2, 66.2 (d, *J* = 5.2 Hz), 63.7 (d, *J* = 3.8 Hz), 47.4, 28.9 (d, *J* = 6.8 Hz), 27.9, 21.6. ^31^P NMR (202 MHz, D_2_O) δ −0.03 (s), −0.14 (s). ESI-MS *m/z* 513 ([M − H]^–^, C_15_H_23_N_4_O_12_P_2_, requires 513).

**11c**: White foam (99% yield). ^1^H NMR (500 MHz, D_2_O) δ 9.05 (s, 1H, 2-H), 8.46 (s, 1H, 8-H), 6.17 (d, *J* = 3.7 Hz, 1H, 1’-H), 4.69–4.65 (m, 1H, 2’-H), 4.44–4.40 (m, 1H, 3’-H), 4.37–4.33 (m, 1H, 4’-H), 4.21 (ddd, *J* = 11.9, 4.0, 2.5 Hz, 1H, 5’-H_a_), 4.13–4.06 (complex signal, 3H, CH_2_OP, 5’-H_b_), 3.90–3.84 (m, 2H, CH_2_N), 1.78–1.68 (m, 2H, CH_2_), 1.62–1.54 (m, 2H, CH_2_), 1.40–1.28 (m, 4H, 2 × CH_2_). ^13^C-NMR (101 MHz; D_2_O): δ 156.0, 150.4, 146.8, 138.8, 117.5, 88.9, 84.0 (d, *J* = 8.8 Hz), 74.7, 69.7, 66.3 (d, *J* = 5.5 Hz), 64.0 (d, *J* = 4.6 Hz), 47.6, 29.4 (d, *J* = 6.6 Hz), 28.4, 25.1, 24.4. ^31^P NMR (202 MHz, D_2_O) δ −0.03 (s), −0.14 (s). ESI-MS *m/z* 527 ([M − H]^–^, C_16_H_25_N_4_O_12_P_2_, requires 527).

#### 3.2.5. General Procedure for the Preparation of Compounds **12a**–**c**

In a typical experiment, to a solution of compound **8a** (0.050 g, 0.13 mmol) in dry THF (2.0 mL), 1-*H*-tetrazole (0.028 g, 0.40 mmol) and then ^i^Pr_2_NP(O^t^Bu)_2_ (0.29 mL, 0.91 mmol) were added under nitrogen atmosphere. The reaction mixture was stirred at room temperature for 6 h (TLC monitoring: AcOEt/MeOH, 95:5) and then ^t^BuOOH (0.24 mL of a solution 5.5 M in decane, 1.3 mmol) was added at room temperature. After 1 h (TLC monitoring: AcOEt/MeOH, 95:5), the reaction mixture was evaporated under reduced pressure, diluted with AcOEt (10 mL), and washed with brine (10 mL). The organic layer was dried over anhydrous Na_2_SO_4_, filtered, and evaporated under reduced pressure. The residue was purified over a silica gel column eluted with increasing amounts of AcOEt in CH_2_Cl_2_ (up to 50%) to afford pure **12a**.

**12a:** Oil (57% yield over two steps). ^1^H NMR (400 MHz, C_6_D_6_) δ 7.80 (s, 1H, 2-H), 7.37 (s, 1H, 8-H), 6.15 (d, *J* = 2.1 Hz, 1H, 1’-H), 5.08 (dd, *J* = 6.2, 2.2 Hz, 1H, 2’-H), 4.93 (dd, *J* = 6.1, 3.0 Hz, 1H, 3’-H), 4.36–4.32 (m, 1H, 4’-H), 3.88–3.80 (m, 2H, CH_2_N), 3.65 (dd, *J* = 11.1, 4.7 Hz, 1H, 5’-H_a_), 3.60–3.49 (complex signal, 3H, CH_2_OP, 5’-H_b_), 1.63–1.53 (m, 2H, CH_2_), 1.50 (s, 3H, CH_3_), 1.41 (s, 18H, 2 × O^t^Bu), 1.37–1.28 (m, 2H, CH_2_), 1.21 (s, 3H, CH_3_), 0.87 (s, 9H, ^t^Bu), −0.04 (s, 3H, SiCH_3_), −0.06 (s, 3H, SiCH_3_). ^13^C NMR (100 MHz, C_6_D_6_) δ 155.9, 147.0, 138.2, 125.5, 113.6, 90.4, 87.1, 85.1, 81.5, 81.1, 81.0, 65.6 (d, *J* = 6.1 Hz), 63.3, 45.5, 29.8, 29.6, 29.5, 27.0, 25.9, 25.6, 25.1, 18.1, −5.70, −5.80. ^31^P NMR (202 MHz, C_6_D_6_) δ –8.50 (s). ESI-MS *m/z* 687 ([M + H]^+^, C_31_H_56_N_4_O_9_PSi, requires 687).

**12b:** Oil (61% yield over two steps). ^1^H NMR (400 MHz, C_6_D_6_) δ 7.81 (s, 1H, 2-H), 7.28 (s, 1H, 8-H), 6.16 (d, *J* = 2.2 Hz, 1H, 1’-H), 5.07 (dd, *J* = 6.2, 2.2 Hz, 1H, 2’-H), 4.92 (dd, *J* = 6.1, 2.9 Hz, 1H, 3’-H), 4.35–4.32 (m, 1H, 4’-H), 3.90–3.84 (m, 2H, CH_2_N), 3.65 (dd, *J* = 11.1, 4.7 Hz, 1H, 5’-H_a_), 3.52 (dd, *J* = 11.1, 4.4 Hz, 1H, 5’-H_b_), 3.50–3.38 (m, 2H, CH_2_OP), 1.50 (s, 3H, CH_3_), 1.43 (s, 18H, 2 × O^t^Bu), 1.42–1.32 (m, 4H, 2 × CH_2_), 1.21 (s, 3H, CH_3_), 1.13–1.04 (m, 2H, CH_2_), 0.86 (s, 9H, ^t^Bu), −0.04 (s, 3H, SiCH_3_), −0.06 (s, 3H, SiCH_3_). ^13^C NMR (100 MHz, C_6_D_6_) δ 156.8, 147.9, 147.8, 139.1, 126.5, 114.6, 91.4, 88.1, 86.0, 82.4, 81.9 (d, *J* = 6.7 Hz), 66.9 (d, *J* = 6.1 Hz), 64.3, 47.0, 30.6, 30.5, 30.4, 29.9, 28.0, 26.6, 26.1, 23.4, 19.1, −4.70, −4.80. ^31^P NMR (202 MHz, C_6_D_6_) δ −8.50 (s). ESI-MS *m/z* 701 ([M + H]^+^, C_32_H_58_N_4_O_9_PSi, requires 701).

**12c:** Oil (60% yield over two steps). ^1^H NMR (400 MHz, C_6_D_6_) δ 7.77 (s, 1H, 2-H), 7.24 (s, 1H, 8-H), 6.16 (d, *J* = 1.8 Hz, 1H, 1’-H), 5.06 (dd, *J* = 6.1, 1.9 Hz, 1H, 2’-H), 4.91 (dd, *J* = 5.9, 2.7 Hz, 1H, 3’-H), 4.36–4.31 (m, 1H, 4’-H), 3.96–3.88 (m, 2H, CH_2_N), 3.64 (dd, *J* = 11.0, 4.6 Hz, 1H, 5’-H_a_), 3.51 (dd, *J* = 11.1, 4.4 Hz, 1H, 5’-H_b_), 3.48–3.36 (m, 2H, CH_2_OP), 1.50 (s, 3H, CH_3_), 1.45 (s, 18H, 2 × O^t^Bu), 1.40–1.30 (m, 4H, 2 × CH_2_), 1.20 (s, 3H, CH_3_), 1.15–1.09 (m, 2H, CH_2_), 1.00–0.90 (m, 2H, CH_2_), 0.86 (s, 9H, ^t^Bu), −0.04 (s, 3H, SiCH_3_), −0.07 (s, 3H, SiCH_3_). ^13^C NMR (100 MHz, C_6_D_6_) δ 156.8, 147.9, 147.8, 126.6, 114.6, 91.4, 88.1, 86.1, 82.4, 81.8 (d, *J* = 6.6 Hz), 67.1 (d, *J* = 6.1 Hz), 64.3, 47.0, 31.0, 30.1, 30.6, 30.5, 30.4, 28.0, 26.8, 26.6, 26.0, 19.0, −4.70, −4.80. ^31^P NMR (202 MHz, C_6_D_6_) δ −8.4 (s). ESI-MS *m/z* 715 ([M + H]^+^, C_33_H_60_N_4_O_9_PSi, requires 715).

#### 3.2.6. General Procedure for the Reparation of Compounds **13a**–**c**

In a typical experiment, to a solution of compound **12a** (0.051 g, 0.074 mmol) in dry THF (1.0 mL), TBAF (0.089 mL of a 1.0 M solution in dry THF, 0.089 mmol) was added dropwise [57]. The reaction mixture was stirred at room temperature for 1 h (TLC monitoring: AcOEt/MeOH; 9:1) and then evaporated under reduced pressure. The residue was purified over a silica gel column eluted with increasing amounts of MeOH in AcOEt (up to 5%) to afford the pure **13a**.

**13a:** Glassy solid (99% yield). ^1^H NMR (500 MHz, C_6_D_6_) δ 8.04 (s, 1H, 2-H), 7.28 (s, 1H, 8-H), 6.15 (d, *J* = 2.2 Hz, 1H, 1’-H), 5.10–5.05 (complex signal, 2H, 2’-H, 3’-H), 4.40–4.36 (m, 1H, 4’-H), 4.07 (d, *J* = 12.0 Hz, 1H, 5’-H_a_), 3.87–3.80 (m, 2H, CH_2_N), 3.75 (d, *J* = 11.9 Hz, 1H, 5’-H_b_), 3.48 (t, *J* = 7.3 Hz, 2H, CH_2_OP), 1.55–1.48 (complex signal, 5H, CH_2_ and CH_3_), 1.40 (s, 18H, 2 × O^t^Bu), 1.37–1.29 (m, 2H, CH_2_), 1.18 (s, 3H, CH_3_). ^13^C NMR (100 MHz, C_6_D_6_) δ 156.9, 148.1, 147.7, 140.2, 126.3, 114.3, 93.2, 87.8, 85.7, 82.9, 82.2 (d, *J* = 6.7 Hz), 66.6 (d, *J* = 6.0 Hz), 63.7, 46.6, 30.5, 30.4, 28.1 (d, *J* = 7.1 Hz), 27.9, 26.8, 25.9. ^31^P NMR (202 MHz, C_6_D_6_) δ −8.70 (s). ESI-MS *m/z* 573 ([M + H]^+^, C_25_H_42_N_4_O_9_P, requires 573).

**13b:** Amorphous white solid (99% yield). ^1^H NMR (500 MHz, C_6_D_6_, 60 °C) δ 7.75 (s, 1H, 2-H), 7.20 (s, 1H, 8-H), 5.92 (d, *J* = 3.3 Hz, 1H, 1’-H), 5.13–5.08 (complex signal, 2H, 2’-H, 3’-H), 4.47–4.44 (m, 1H, 4’-H), 3.89–3.93 (m, 1H, 5’-H_a_), 3.89–3.83 (m, 2H, CH_2_N), 3.67 (d, *J* = 10.7 Hz, 1H, 5’-H_b_), 3.49–3.38 (m, 2H, CH_2_OP), 1.49 (s, 3H, CH_3_), 1.46–1.38 (complex signal, 20H, CH_2_, 2 × O^t^Bu), 1.37–1.31 (m, 2H, CH_2_), 1.18 (s, 3H, CH_3_), 1.15–1.07 (m, 2H, CH_2_). ^13^C NMR (100 MHz, C_6_D_6_, 60 °C) δ 156.8, 148.0, 147.7, 140.2, 126.3, 114.3, 93.2, 87.8, 85.7, 82.9, 82.0, 66.9, 63.8, 47.1, 30.6, 30.5, 30.4, 29.8, 28.2, 25.9, 23.4. ^31^P NMR (202 MHz, C_6_D_6_, 60 °C) δ −8.70 (s). ESI-MS *m/z* 587 ([M + H]^+^, C_26_H_44_N_4_O_9_P, requires 587).

**13c:** Amorphous white solid (99% yield). ^1^H NMR (500 MHz, C_6_D_6_, 60 °C) δ 7.70 (s, 1H, 2-H), 7.18 (s, 1H, 8-H), 5.90 (d, *J* = 3.5 Hz, 1H, 1’-H), 5.09–5.04 (complex signal, 2H, 2’-H, 3’-H), 4.39–4.35 (m, 1H, 4’-H), 3.97–3.88 (complex signal, 3H, 5’-H_a_, CH_2_N), 3.67 (d, *J* = 12.0 Hz, 1H, 5’-H_b_), 3.43 (t, *J* = 7.3 Hz, 2H, CH_2_OP), 1.49 (s, 3H, CH_3_), 1.44 (s, 18H, 2 × O^t^Bu), 1.40–1.29 (m, 4H, 2 × CH_2_), 1.22–1.12 (complex signal, 5H, CH_2_, CH_3_), 1.03–0.95 (m, 2H, CH_2_). ^13^C NMR (100 MHz, C_6_D_6_, 60 °C) δ 156.8, 148.0, 147.7, 140.2, 126.3, 114.3, 93.2, 87.8, 85.7, 82.9, 82.0, 66.9, 63.8, 47.1, 30.6, 30.5, 30.4, 29.8, 28.2, 25.9, 23.4. ^31^P NMR (202 MHz, C_6_D_6_, 60 °C) δ −8.70 (s). ESI-MS *m/z* 601 ([M + H]^+^, C_27_H_46_N_4_O_9_P, requires 601).

#### 3.2.7. General Procedure for the Preparation of Compounds **14a**–**c**

In a typical experiment, compound **13a** (0.020 g, 0.035 mmol) was dissolved in a 1:1 (v/v) solution of TFA in H_2_O (1.0 mL) at 0 °C. The reaction mixture was warmed to room temperature, stirred for 4 h (TLC monitoring: *^i^*PrOH/NH_3_(aq)/H_2_O, 6:3:1) and then lyophilized.

**14a**: White foam (99% yield). ^1^H NMR (500 MHz, D_2_O) δ 9.09 (s, 1H, 2-H), 8.51 (s, 1H, 8-H), 6.16 (d, *J* = 4.2 Hz, 1H, 1’-H), 4.72–4.68 (m, 1H, 2’-H), 4.39–4.35 (m, 1H, 3’-H), 4.28–4.24 (m, 1H, 4’-H), 4.17 (t, *J* = 7.2 Hz, 2H, CH_2_OP), 4.00–3.95 (m, 2H, CH_2_N), 3.92 (dd, *J* = 13.0, 2.0 Hz, 1H, 5’-H_a_), 3.82 (d, *J* = 12.8, 3.5 Hz, 1H, 5’-H_b_), 1.91–1.83 (m, 2H, CH_2_), 1.74–1.66 (m, 2H, CH_2_). ^13^C NMR (175 MHz, D_2_O) δ 154.8, 151.0, 146.0, 138.8, 118.1, 89.8, 85.3, 74.1, 69.4, 66.0, 60.4, 47.1, 26.4, 24.9. ^31^P NMR (202 MHz, D_2_O) δ −0.14 (s). ESI-MS *m/z* 419 ([M − H]^–^, C_14_H_20_N_4_O_9_P, requires 419).

**14b**: White foam (99% yield). ^1^H NMR (500 MHz, D_2_O) δ 9.01 (s, 1H, 2-H), 8.50 (s, 1H, 8-H), 6.16 (d, *J* = 4.4 Hz, 1H, 1’-H), 4.75–4.71 (m, 1H, 2’-H), 4.42–4.38 (m, 1H, 3’-H), 4.30–4.26 (m, 1H, 4’-H), 4.15 (t, *J* = 7.2 Hz, 2H, CH_2_OP), 4.00–3.92 (complex signal, 3H, CH_2_N, 5’-H_a_), 3.84 (d, *J* = 12.8, 3.9 Hz, 1H, 5’-H_b_), 1.86–1.78 (m, 2H, CH_2_), 1.73–1.65 (m, 2H, CH_2_), 1.48–1.39 (m, 2H, CH_2_). ^13^C NMR (175 MHz, D_2_O) δ 155.2, 150.7, 146.2, 139.0, 118.9, 89.6, 85.3, 74.1, 69.6, 66.3, 60.5, 47.4, 28.9, 27.9, 21.6. ^31^P NMR (202 MHz, D_2_O) δ −0.04 (s). ESI-MS *m/z* 433 ([M − H]^–^, C_15_H_22_N_4_O_9_P, requires 433).

**14c**: White foam (99% yield). ^1^H NMR (500 MHz, D_2_O) δ 8.92 (s, 1H, 2-H), 8.49 (s, 1H, 8-H), 6.16 (d, *J* = 4.4 Hz, 1H, 1’-H), 4.76–4.72 (m, 1H, 2’-H), 4.44–4.39 (m, 1H, 3’-H), 4.31–4.27 (m, 1H, 4’-H), 4.15 (t, *J* = 7.2 Hz, 2H, CH_2_OP), 3.97–3.91 (complex signal, 3H, CH_2_N, 5’-H_a_), 3.84 (d, *J* = 12.8, 3.9 Hz, 1H, 5’-H_b_), 1.84–1.76 (m, 2H, CH_2_), 1.69–1.61 (m, 2H, CH_2_), 1.46–1.34 (m, 4H, 2 × CH_2_). ^13^C NMR (175 MHz, D_2_O) δ 155.6, 150.2, 146.3, 139.1, 119.5, 89.4, 85.3, 74.1, 69.5, 66.6, 60.6, 47.5, 29.2, 28.3, 25.0, 24.2. ^31^P NMR (202 MHz, D_2_O) δ 0.04 (s). ESI-MS *m/z* 447 ([M − H]^–^, C_16_H_24_N_4_O_9_P, requires 447).

#### 3.2.8. Procedure for the Preparation of Compound **16**

Compound **15** (0.020 g, 0.042 mmol) was dissolved in a 1:1 (v/v) solution of TFA in H_2_O (0.5 mL) at 0 °C. The reaction mixture was warmed to room temperature, stirred for 4 h (TLC monitoring: *^i^*PrOH/NH_3_(aq)/H_2_O, 6:3:1) and then lyophilized. Colorless syrup (99% yield). ^1^H-NMR (500 MHz; D_2_O): δ 8.53 (s, 1H, 8-H), 8.42 (s, 1H, 2-H), 6.15 (d, *J* = 5.9 Hz, 1H, 1’-H), 4.84–4.78 (m, 1H, 2’-H, partially covered by residual solvent signal), 4.54 (dd, *J* = 4.9, 3.7 Hz, 1H, 3’-H), 4.41–4.40 (m, 1H, 4’-H), 4.18 (t, *J* = 7.3 Hz, 2H, CH_2_N), 4.12–4.08 (m, 2H, 5’-H_a,b_), 3.63 (t, *J* = 6.5 Hz, 2H, CH_2_O), 1.88–1.82 (m, 2H, CH_2_), 1.66–1.60 (m, 2H, CH_2_), 1.47–1.40 (m, 2H, CH_2_). ^13^C-NMR (101 MHz; D_2_O): δ 158.1, 148.9, 147.9, 139.9, 123.4, 87.2, 84.2 (d, *J* = 8.8 Hz), 74.4, 70.4, 64.3 (d, *J* = 4.8 Hz), 61.4, 47.3, 30.8, 28.5, 22.0. ^31^P NMR (202 MHz, D_2_O) δ 1.87 (s). ESI-MS *m/z* 433 ([M − H]^–^, C_15_H_22_N_4_O_9_P, requires 433).

#### 3.2.9. Procedure for the Preparation of Compound **18**

Compound **17** (0.040 g, 0.070 mmol) was dissolved in a 1:1 (v/v) solution of TFA in H_2_O (0.5 mL) at 0 °C. The reaction mixture was warmed to room temperature, stirred for 4 h (TLC monitoring: *^i^*PrOH/NH_3_(aq)/H_2_O, 6:3:1), and then lyophilized. The crude was dissolved in TEAB 0.1 M buffer, passed over a PVDF 0.45 μm filter and purified by HPLC (see Section 3.1). The fractions containing the triethylammonium salt of compound **18** were collected and lyophilized. The salt was dissolved in water and treated with Dowex D50 (H^+^ form). Then, the resin was removed by filtration and the solution lyophilized. Colorless syrup (50% yield). ^1^H-NMR (400 MHz; D_2_O): δ 8.41 (s, 1H, 8-H), 8.33 (s, 1H, 2-H), 6.08 (d, *J* = 5.7 Hz, 1H, 1’-H), 4.80 (m, 1H, 2’-H, covered by residual solvent signal), 4.47–4.42 (m, 1H, 3’-H), 4.31–4.26 (m, 1H, 4’-H), 4.15 (t, *J* = 7.2 Hz, 2H, CH_2_N), 3.92 (dd, *J* = 12.7, 2.8 Hz, 1H, 5’-H_a_), 3.85 (dd, *J* = 12.8, 4.1 Hz, 1H, 5’-H_b_), 1.86–1.77 (m, 2H), 1.65–1.53 (m, 4H), 1.48–1.41 (m, 2H). ^13^C NMR (101 MHz; D_2_O): δ 155.3, 150.6, 146.3, 139.2, 119.0, 89.7, 85.5, 74.3, 69.6, 60.6, 47.4, 28.0, 26.4 (*J* = 3.6 Hz), 25.7 (*J* = 121.3 Hz), 21.5 (*J* = 4.8 Hz). ^31^P NMR (202 MHz; D_2_O): δ 31.9. ESI-MS *m/z* 417 ([M − H]^–^, C_15_H_22_N_4_O_8_P, requires 417).

### 3.3. Biology

#### Cytosolic Ca^2+^ Imaging

Differentiated C2C12 (days in vitro [DIV] 7) cells were incubated with 1 μM Fura-2/AM, 0.02% pluronic F-127, and 200 μM sulfinpyrazone for 40 minutes at 37 °C in an extracellular-like medium 135 mM NaCl, 5 mM KCl, 0.4 mM KH_2_PO_4_, 1 mM MgCl_2_, 1 mM MgSO_4_, 1 mM CaCl_2_, 10 mM 4-[2-hydroxyethyl]-1-piperazineethanesulfonic acid, and 10 mM glucose at pH 7.4. Cells were mounted into an open-topped chamber and incubated for 10 minutes on the stage of the microscope, in the same saline without Fura-2/AM. Experiments were performed at 37 °C. Fura-2 fluorescence was visualized on an inverted microscope (Zeiss Axiovert 100, Jena, Germany) mounting a 20× ultraviolet permeable objective (Olympus Biosystems GmbH, Planegg, Germany). Alternating excitation wavelengths of 340 and 380 nm were obtained with a monochromator (polychrome V; TILL Photonics, Kaufbeuren, Germany) controlled by a custom-made software package, Roboscope (developed by Catalin Dacian Ciubotaru, CNR Neuroscience Institute, Padua, Italy). A neutral density filter, UVND 0.6 (Chroma Technology Corp., Bellows Falls, VT, USA), was used in the excitation pathway. The emitted fluorescence was measured at 500–530 nm. Images were acquired every 10 s, with 200 ms exposure time for each wavelength, by a TILL-Imago camera controlled by the same software. Regions of interest, corresponding to the entire soma, were selected for Ca^2+^ imaging. The ratio of the emitted fluorescence intensities (F340/F380) was normalized to the average value measured during the first 2 min of acquisition. The cells were challenged with different concentrations of target compounds diluted in the experimental medium; addition of 10 mM caffeine was used as a control for Ca^2+^ release from ryanodine receptors.

## 4. Conclusions

Cellular Ca^2+^ mobilization is involved in several physio-pathological processes, and the discovery of molecules that could act as agonist or antagonist of cADPR is an appealing goal for medicinal chemistry. cADPR has a unique structure, but its lability has strongly limited the comprehension of cellular mechanisms regulated by Ca^2+^ mobilization. Synthetic chemistry partially remedied this problem, generating more stable analogues whose preparation is very often laborious and low yielding. In our laboratories, we synthesized many cIDPR derivatives as stable mimics of cADPR, discovering that a pentyl chain replacing the “northern” ribose and one phosphate group could be sufficient to retain the biological activity in PC12 cells differentiated in neurons. In this paper, we probed if the cyclic structure was strictly necessary for the Ca^2+^ release from the intracellular stores, synthesizing a small collection of linear precursors of cIDPR analogues. By varying the length of the N1 purine alkyl chain and the position of the phosphate/phosphonate moieties, we obtained eight derivatives that were tested for the Ca^2+^ mobilizing activity on C2C12 cells that express ryanodine receptors during differentiation [52].

Unfortunately, we have not been able yet to detect any biological effect immediately after the drug addition to the cell culture. We also cannot establish if the lack of activity can be attributed to the absence of cyclic structure, to an inefficacious interaction with the cellular receptor or to a reduced cellular permeability. Furthermore, the reinforcement of the effect of caffeine on the Ca^2+^ release from the stores by some compounds is intriguing and may be ascribed to a stimulus of Ca^2+^ release from the ER, increasing the Ca^2+^ sensitivity of ryanodine receptors. However, further studies are ongoing in our laboratories to check this assumption and to understand how the reinforcement mentioned above could take place. The most pronounced effect has been found in the linear compounds **14** and **16** characterized by a pentyl chain attached at the N1 position of inosine. These preliminary data reinforce our precedent findings [36,37] on the importance of the five carbon atoms alkyl chain for the design of novel linear and cyclic cADPR/cIDPR analogues to be employed as Ca^2+^ modulators.

## Supplementary Materials

The following are available online at https://www.mdpi.com/1660-3397/17/8/476/s1, S1–S23: copies of ^1^H-NMR and ^31^P-NMR spectra of compounds **8a**–**c**, **9a**–**c**, **10a**–**c**, **11a**–**c**, **12a**–**c**, **13a**–**c**, **14a**–**c**, **16**, and **18**; S24–S46: copies of ^13^C-NMR spectra of compounds **8a**–**c**, **9a**–**c**, **10a**–**c**, **11a**–**c**, **12a**–**c**, **13a**–**c**, **14a**–**c**, **16**, and **18**; S-47: Figure S3.
