# Supplementary material for: New Linear Precursors of cIDPR Derivatives as Stable Analogs of cADPR: A Potent Second Messenger with Ca2+-Modulating Activity Isolated from Sea Urchin Eggs
Source: Mar Drugs. 2019 Aug 17;17(8):476. doi: 10.3390/md17080476 (PMC6723567; doi:10.3390/md17080476)
Supplement: Supplementary file 1 [file marinedrugs-17-00476-s001.pdf]

New Linear Precursors of cIDPR Derivatives as Stable Analogs of cADPR: A Potent Second Messenger with Ca^{2+}-Modulating Activity Isolated from Sea Urchin Eggs

Stefano D'Errico [1,2], Emy Basso [3,4], Andrea Patrizia Falanga [5], Maria Marzano [1], Tullio Pozzan [3,4,6], Vincenzo Piccialli [7], Gennaro Piccialli [1,2], Giorgia Oliviero [5,*] and Nicola Borbone [1]

[1] Dipartimento di Farmacia, Università degli Studi di Napoli Federico II, via Domenico Montesano 49, 80131 Napoli, Italy
[2] ISBE Italy/SYSBIO Centro di System Biology, Università di Milano-Bicocca, piazza delle Scienze 2, 20126 Milano, Italy
[3] Consiglio Nazionale delle Ricerche, Dipartimento di Scienze Biomediche, Istituto di Neuroscienze (Sezione di Padova), viale Giuseppe Colombo 3, 35131 Padova, Italy
[4] Dipartimento di Scienze Biomediche, Università degli Studi di Padova, via Ugo Bassi 58/b, 35131 Padova, Italy
[5] Dipartimento di Medicina Molecolare e Biotecnologie Mediche, Università degli Studi di Napoli Federico II, via Sergio Pansini 5, 80131 Napoli, Italy
[6] Istituto Veneto di Medicina Molecolare, via Orus 2, 35129 Padova, Italy
[7] Dipartimento di Scienze Chimiche, Università degli Studi di Napoli Federico II, via Cintia 26, 80126 Napoli, Italy

* Correspondence: golivier@unina.it (G.O.); Tel.: +39-081-679896

^{1}H- and ^{31}P-NMR spectra of compound **8a** S1

^{1}H- and ^{31}P-NMR spectra of compound **8b** S2

^{1}H- and ^{31}P-NMR spectra of compound **8c** S3

^{1}H- and ^{31}P-NMR spectra of compound **9a** S4

^{1}H- and ^{31}P-NMR spectra of compound **9b** S5

^{1}H- and ^{31}P-NMR spectra of compound **9c** S6

^{1}H- and ^{31}P-NMR spectra of compound **10a** S7

^{1}H- and ^{31}P-NMR spectra of compound **10b** S8

^{1}H- and ^{31}P-NMR spectra of compound **10c** S9

^{1}H- and ^{31}P-NMR spectra of compound **11a** S10

^{1}H- and ^{31}P-NMR spectra of compound **11b** S11

^{1}H- and ^{31}P-NMR spectra of compound **11c** S12

^{1}H- and ^{31}P-NMR spectra of compound **12a** S13

^{1}H- and ^{31}P-NMR spectra of compound **12b** S14

^{1}H- and ^{31}P-NMR spectra of compound **12c** S15

^{1}H- and ^{31}P-NMR spectra of compound **13a** S16

^{1}H- and ^{31}P-NMR spectra of compound **13b** S17

^{1}H- and ^{31}P-NMR spectra of compound **13c** S18

^{1}H- and ^{31}P-NMR spectra of compound **14a** S19

^{1}H- and ^{31}P-NMR spectra of compound **14b** S20

^{1}H- and ^{31}P-NMR spectra of compound **14c** S21

^{1}H- and ^{31}P-NMR spectra of compound **16** S22

^{1}H- and ^{31}P-NMR spectra of compound **18** S23

^{13}C-NMR spectrum of compound **8a** S24

^{13}C-NMR spectrum of compound **8b** S25

^{13}C-NMR spectrum of compound **8c** S26

^{13}C-NMR spectrum of compound **9a** S27

^{13}C-NMR spectrum of compound **9b** S28

^{13}C-NMR spectrum of compound **9c** S29

^{13}C-NMR spectrum of compound **10a** S30

^{13}C-NMR spectrum of compound **10b** S31

^{13}C-NMR spectrum of compound **10c** S32

^{13}C-NMR spectrum of compound **11a** S33

^{13}C-NMR spectrum of compound **11b** S34

^{13}C-NMR spectrum of compound **11c** S35

^{13}C-NMR spectrum of compound **12a** S36

^{13}C-NMR spectrum of compound **12b** S37

^{13}C-NMR spectrum of compound **12c** S38

^{13}C-NMR spectrum of compound **13a** S39

^{13}C-NMR spectrum of compound **13b** S40

^{13}C-NMR spectrum of compound **13c** S41

^{13}C-NMR spectrum of compound **14a** S42

^{13}C-NMR spectrum of compound **14b** S43

^{13}C-NMR spectrum of compound **14c** S44

^{13}C-NMR spectrum of compound **16** S45

^{13}C-NMR spectrum of compound **18** S46

Figure 3 S47

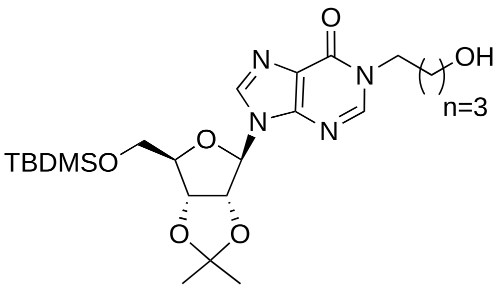
O
N
N
N
N
OH
n=3
O
TBDMSO
O
O

8a

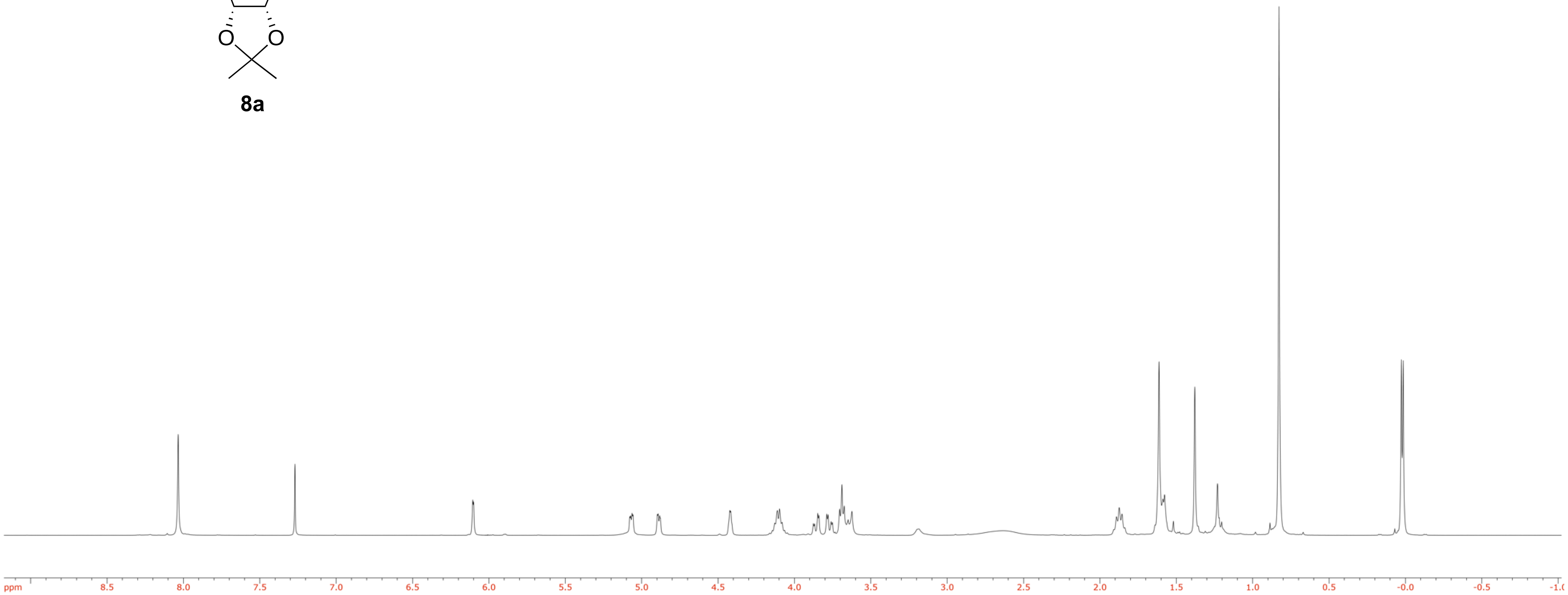
ppm
8.5
8.0
7.5
7.0
6.5
6.0
5.5
5.0
4.5
4.0
3.5
3.0
2.5
2.0
1.5
1.0
0.5
-0.0
-0.5

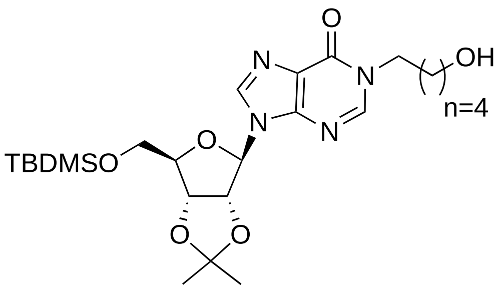
O
N
N
N
N
OH
n=4
TBDMSO
O
O
O

8b

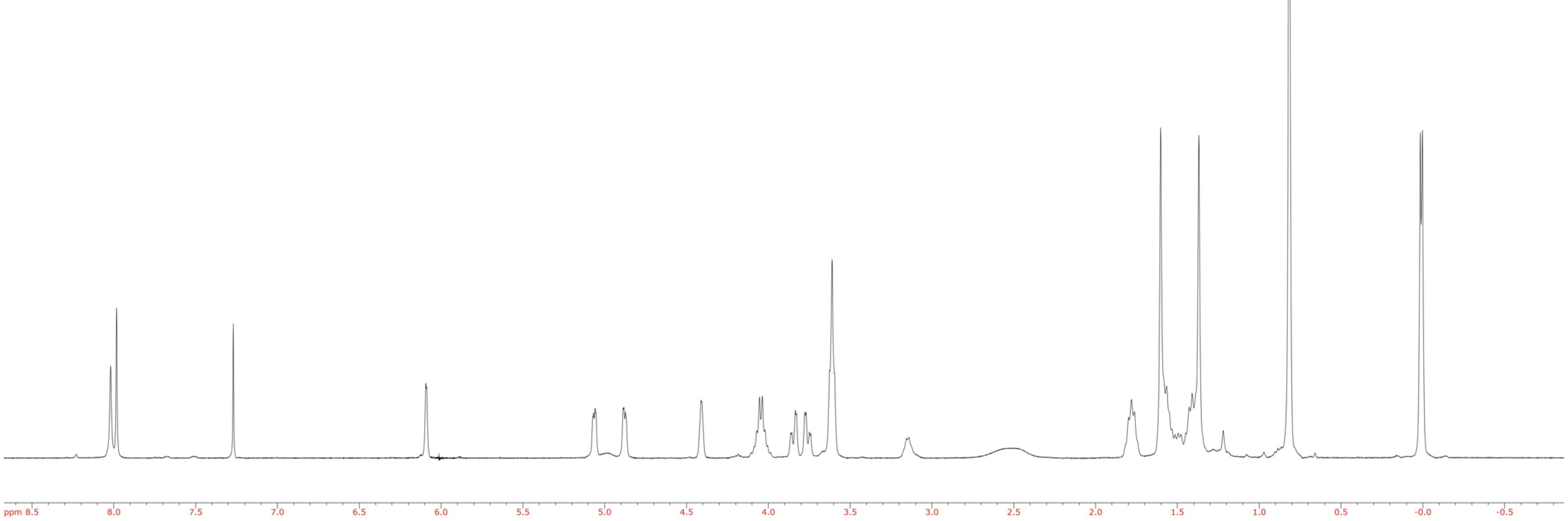
ppm 8.5
8.0
7.5
7.0
6.5
6.0
5.5
5.0
4.5
4.0
3.5
3.0
2.5
2.0
1.5
1.0
0.5
-0.0
-0.5

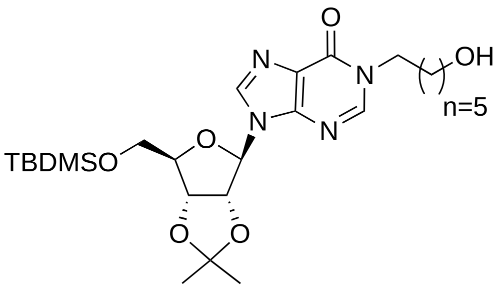

O
N
N
OH
n=5
N
N
O
TBDMSO
O
O

8c

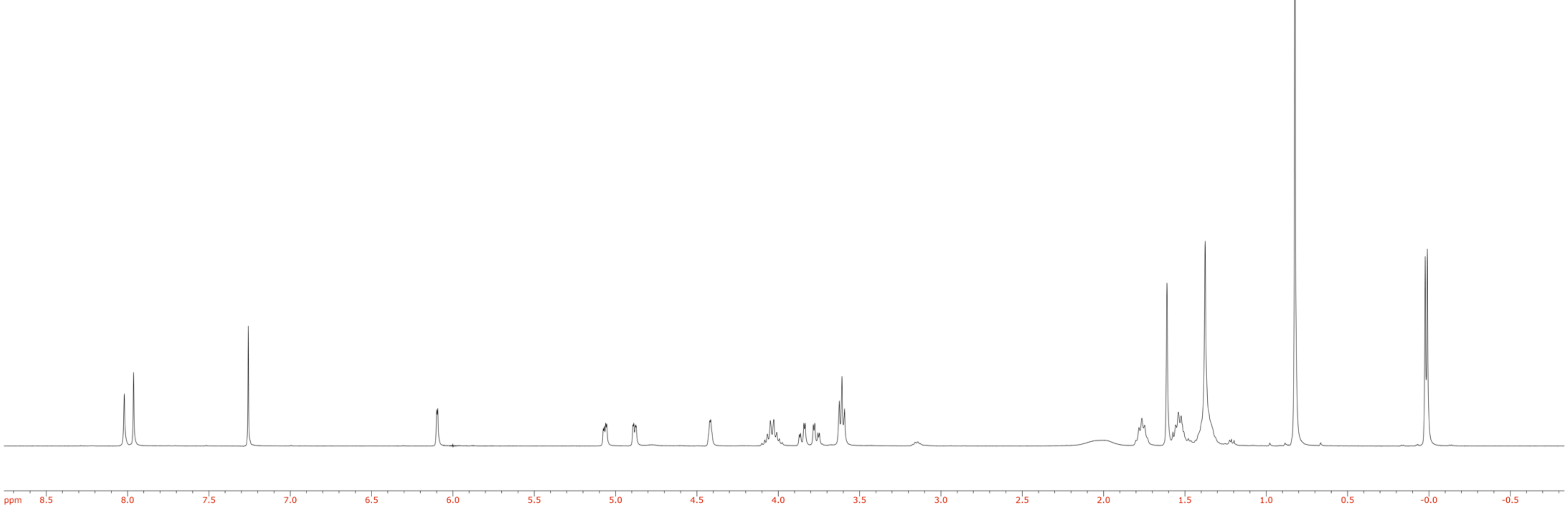

ppm 8.5 8.0 7.5 7.0 6.5 6.0 5.5 5.0 4.5 4.0 3.5 3.0 2.5 2.0 1.5 1.0 0.5 -0.0 -0.5

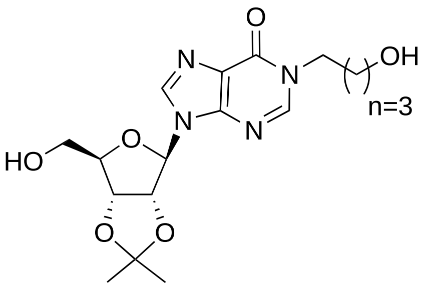

9a

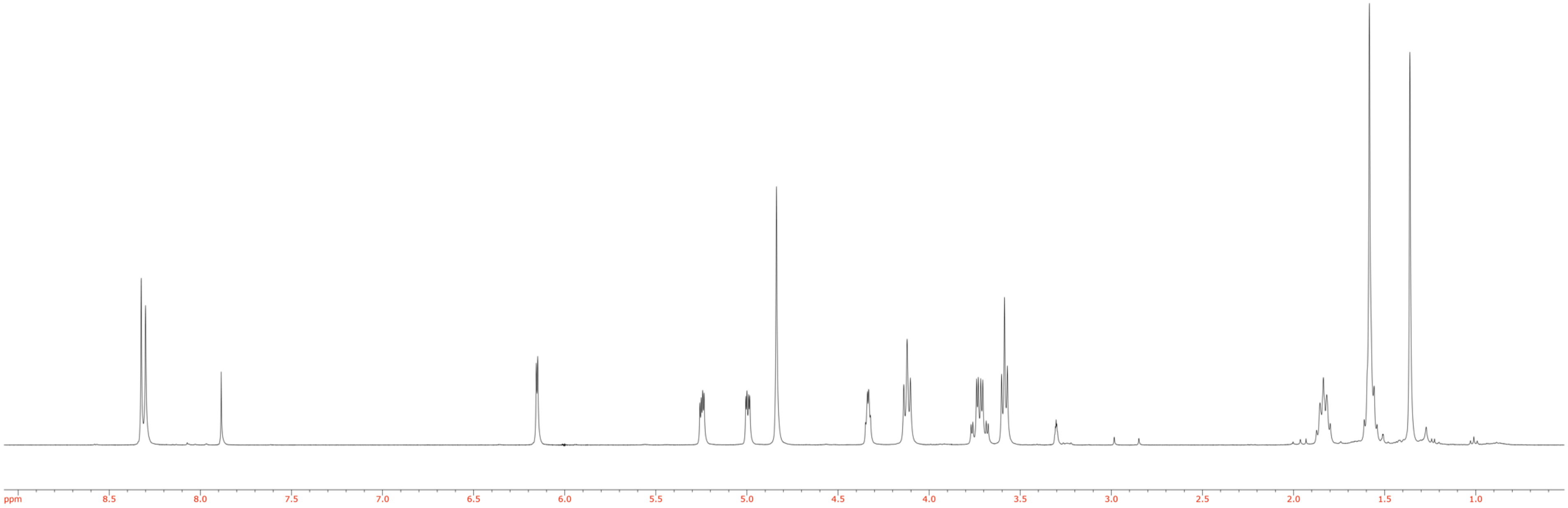

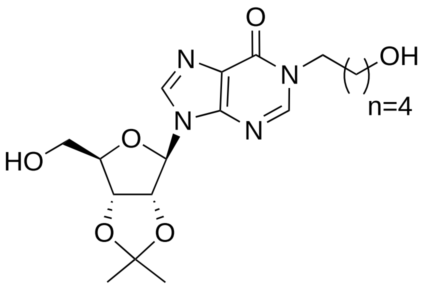
O
N
N
OH
n=4
N
N
O
HO
O
O

9b

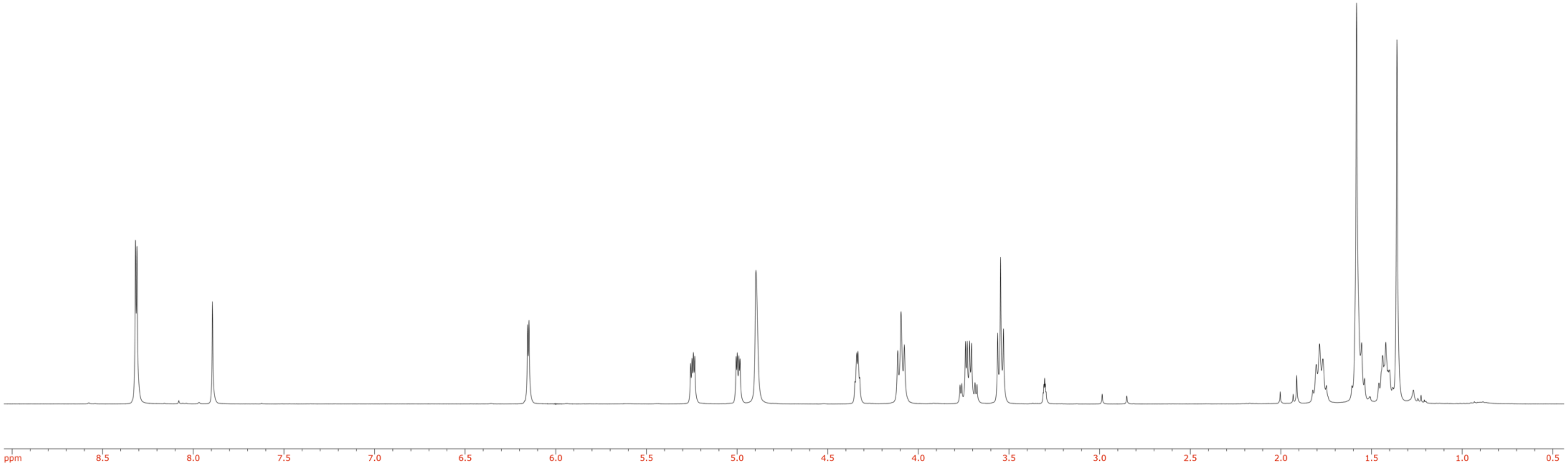
ppm
8.5
8.0
7.5
7.0
6.5
6.0
5.5
5.0
4.5
4.0
3.5
3.0
2.5
2.0
1.5
1.0
0.5

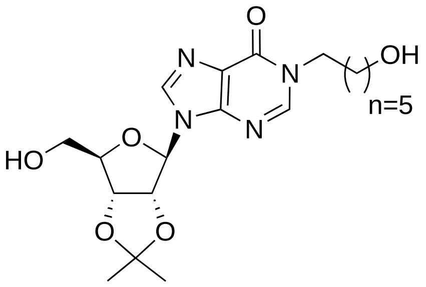

9c

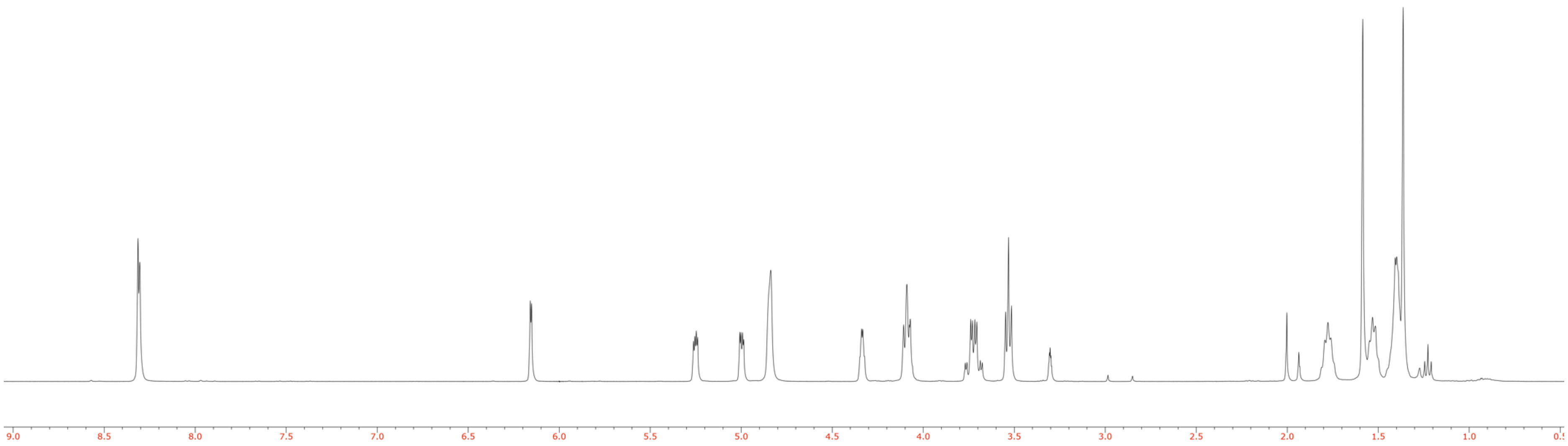

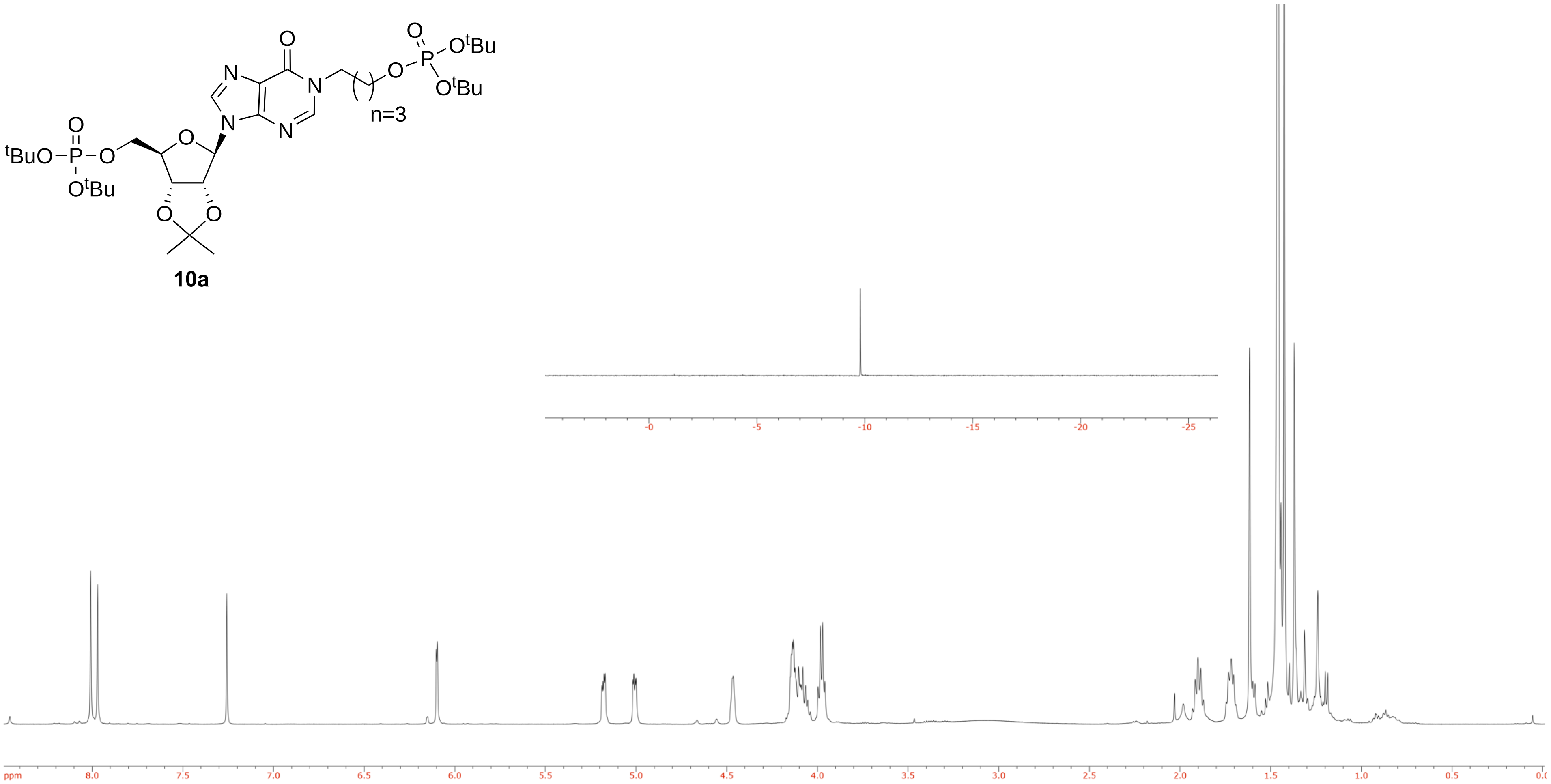

10a

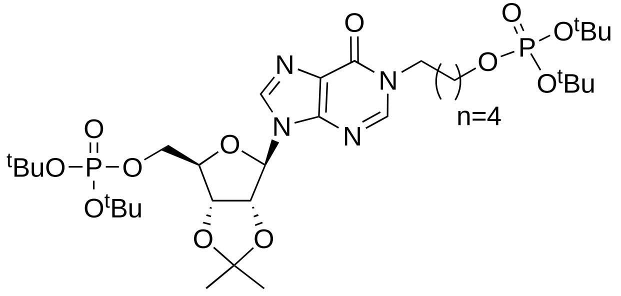
O
N
N
N
N
n=4
O
P
O^{t}Bu
O^{t}Bu
^{t}BuO
P
O
O^{t}Bu
O
O
O

10b

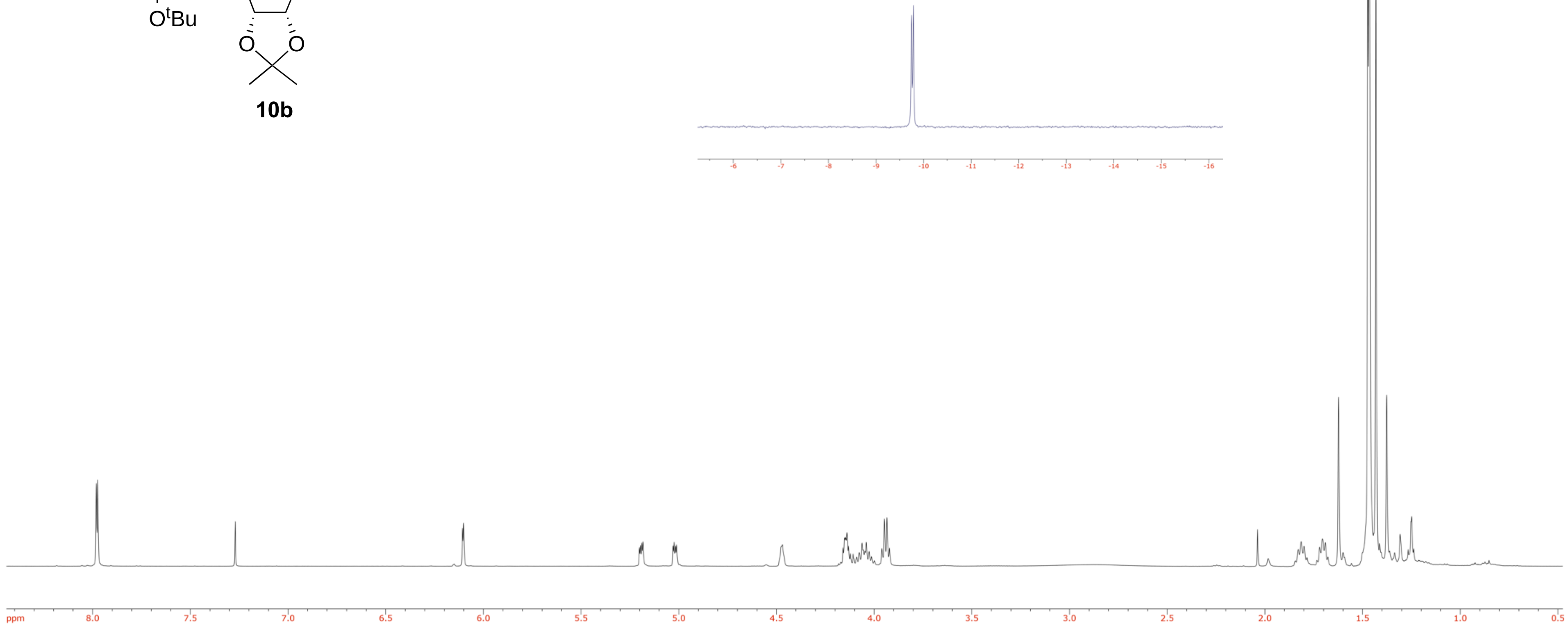
-6 -7 -8 -9 -10 -11 -12 -13 -14 -15 -16
ppm 8.0 7.5 7.0 6.5 6.0 5.5 5.0 4.5 4.0 3.5 3.0 2.5 2.0 1.5 1.0 0.5

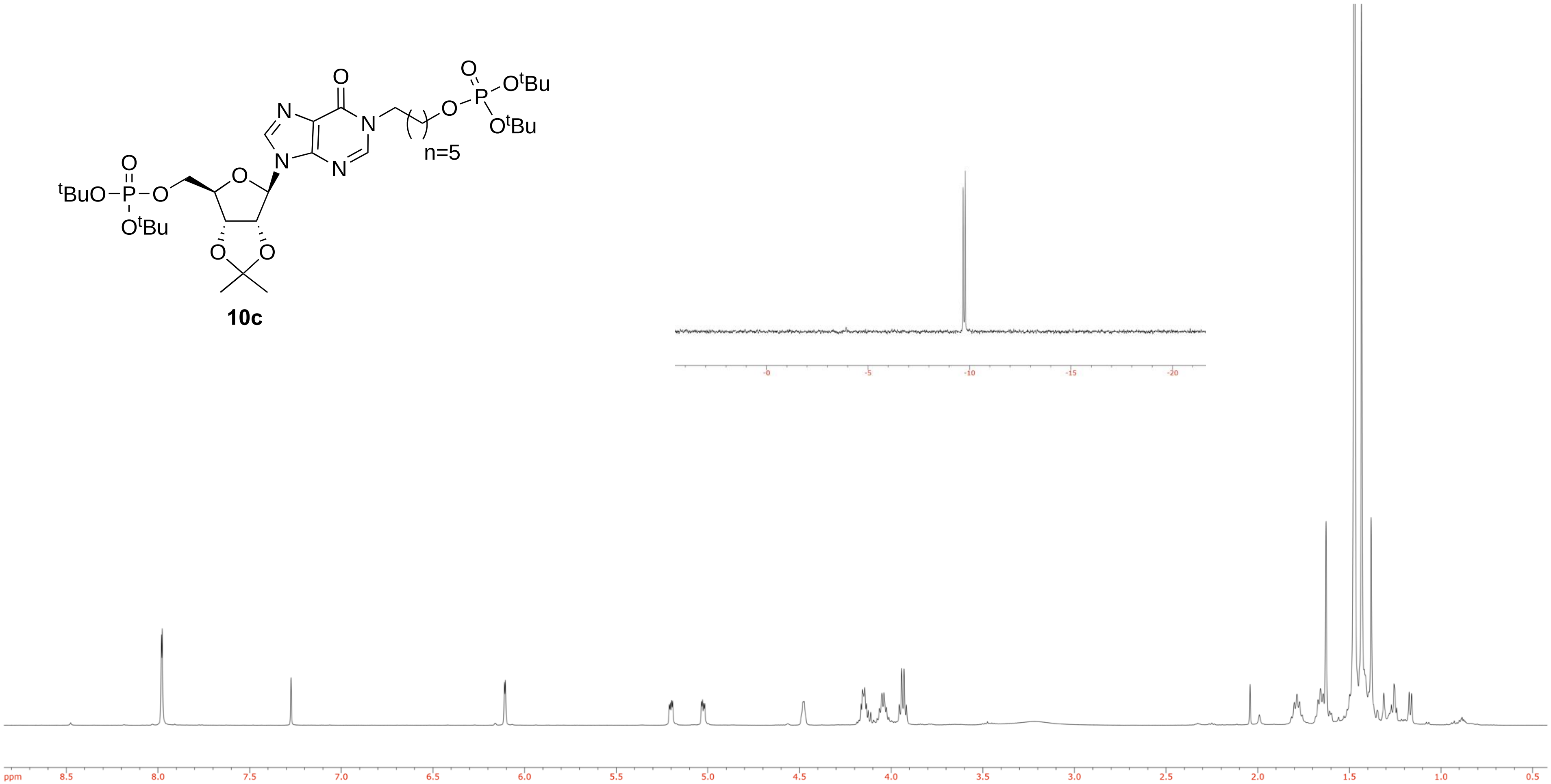
n=5
10c
-0
-5
-10
-15
-20
ppm
8.5
8.0
7.5
7.0
6.5
6.0
5.5
5.0
4.5
4.0
3.5
3.0
2.5
2.0
1.5
1.0
0.5

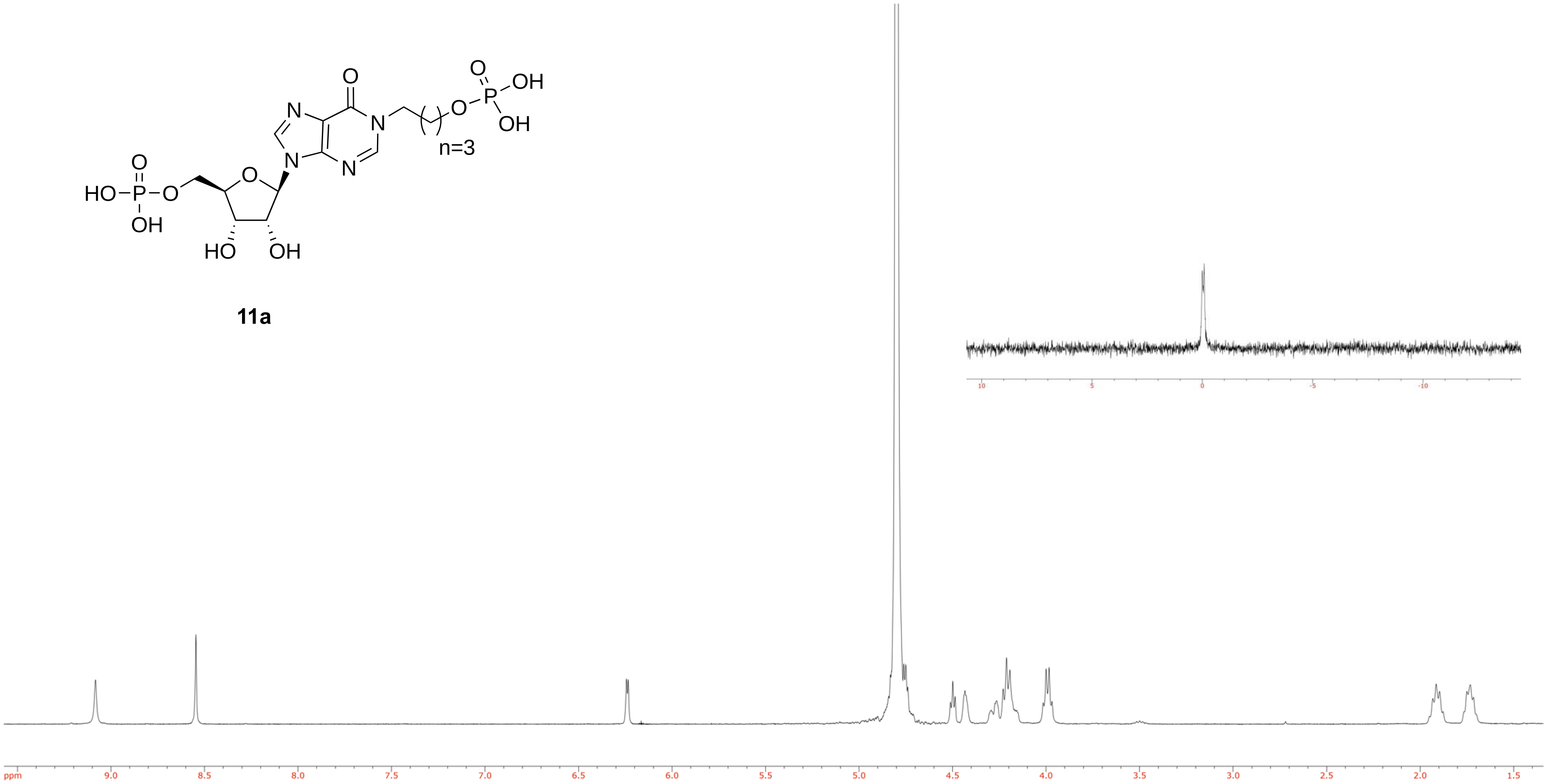

11a

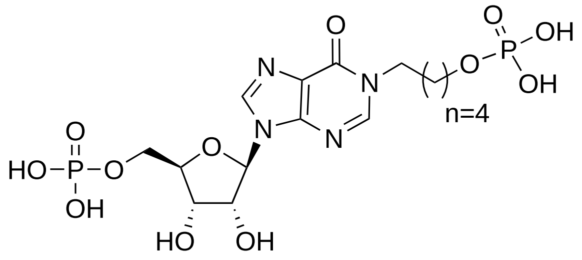

11b

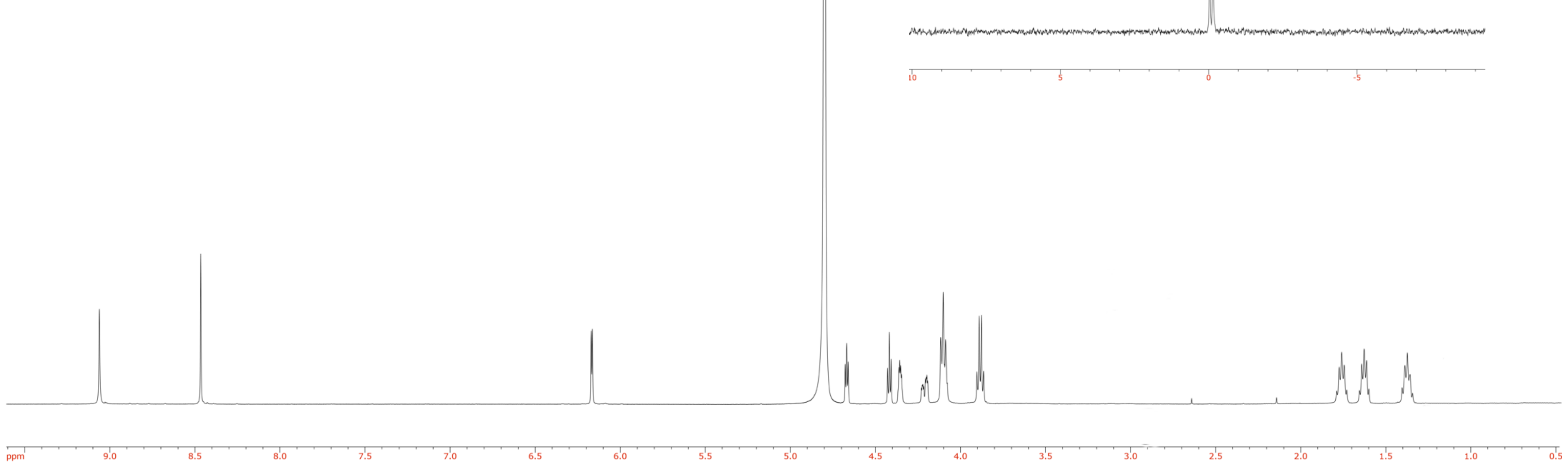

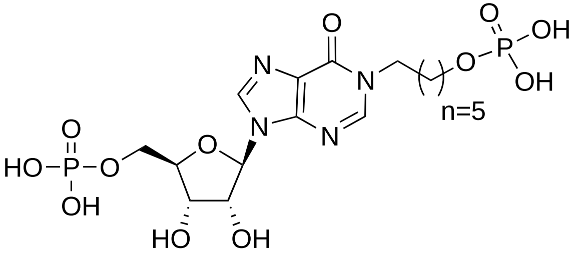

11c

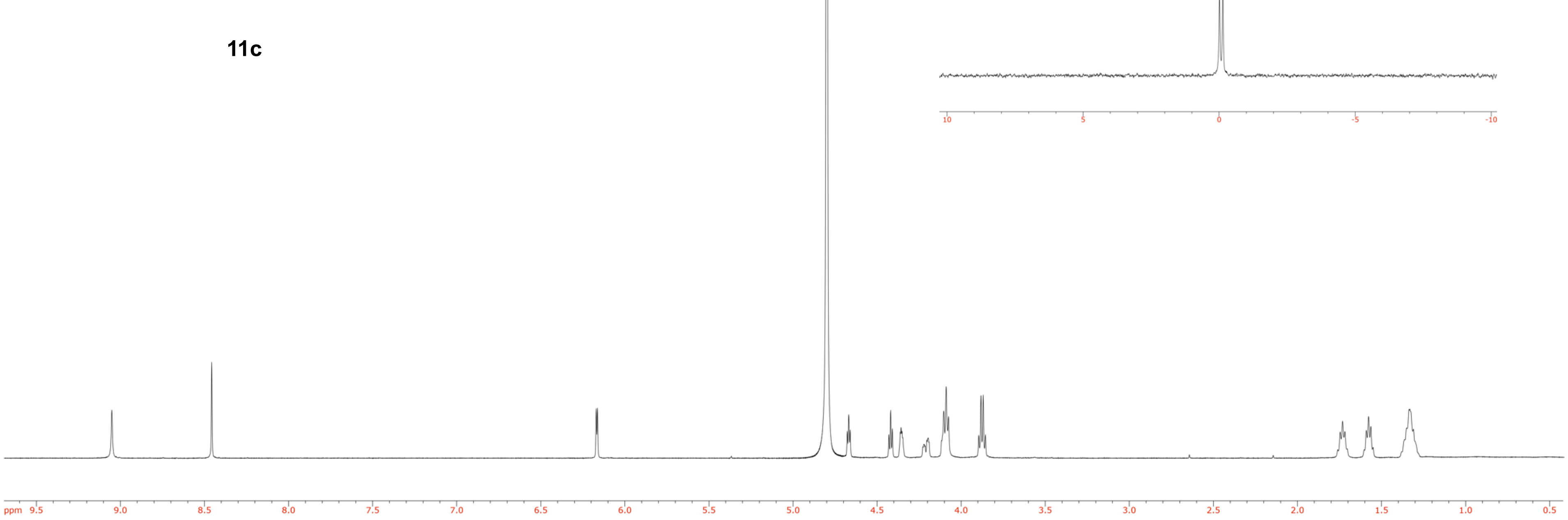

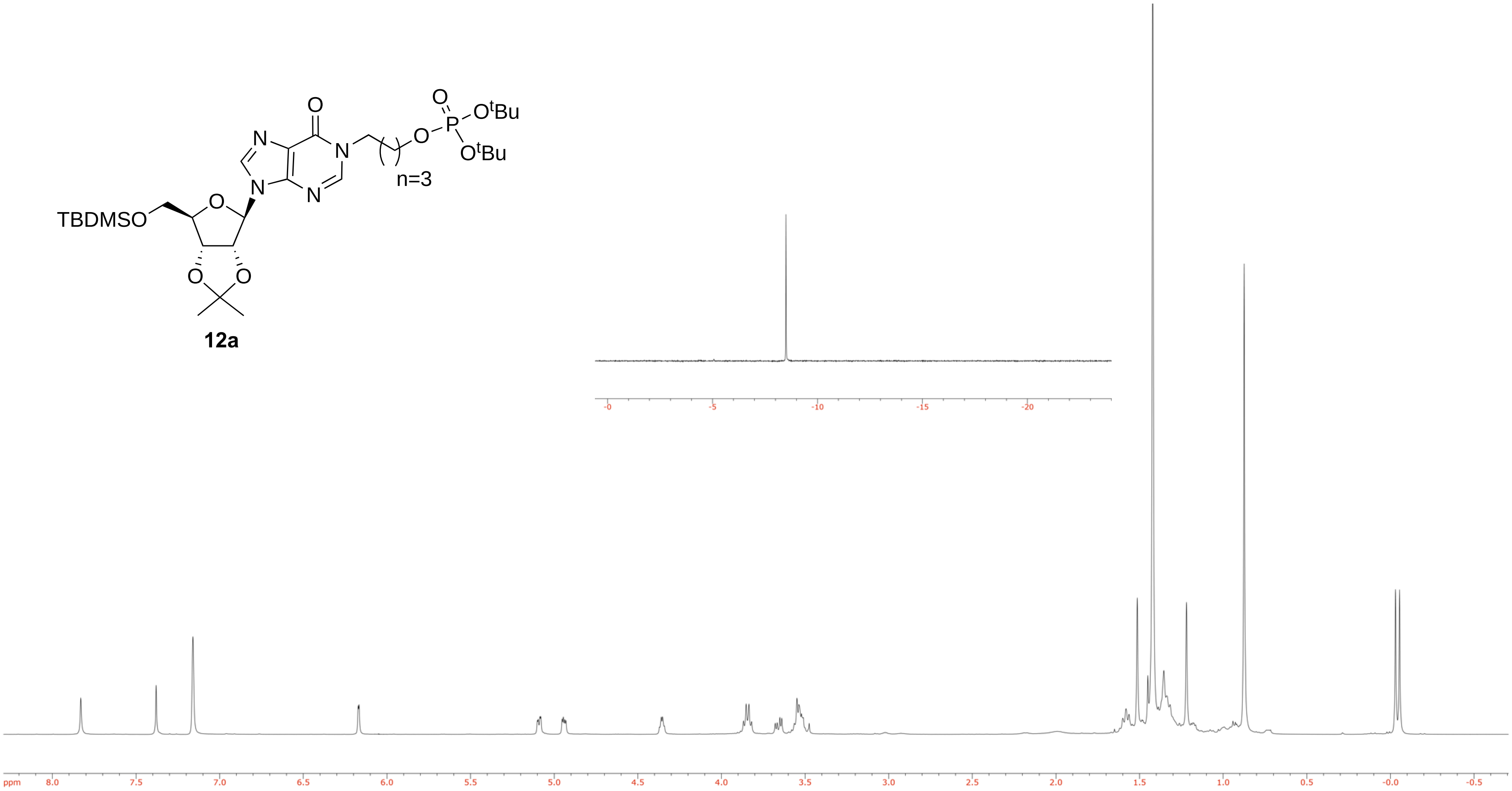
O
N
N
N
N
O
O
P
O^{t}Bu
O^{t}Bu
n=3
TBDMSO
O
O
O
12a
-0
-5
-10
-15
-20
ppm
8.0
7.5
7.0
6.5
6.0
5.5
5.0
4.5
4.0
3.5
3.0
2.5
2.0
1.5
1.0
0.5
-0.0
-0.5

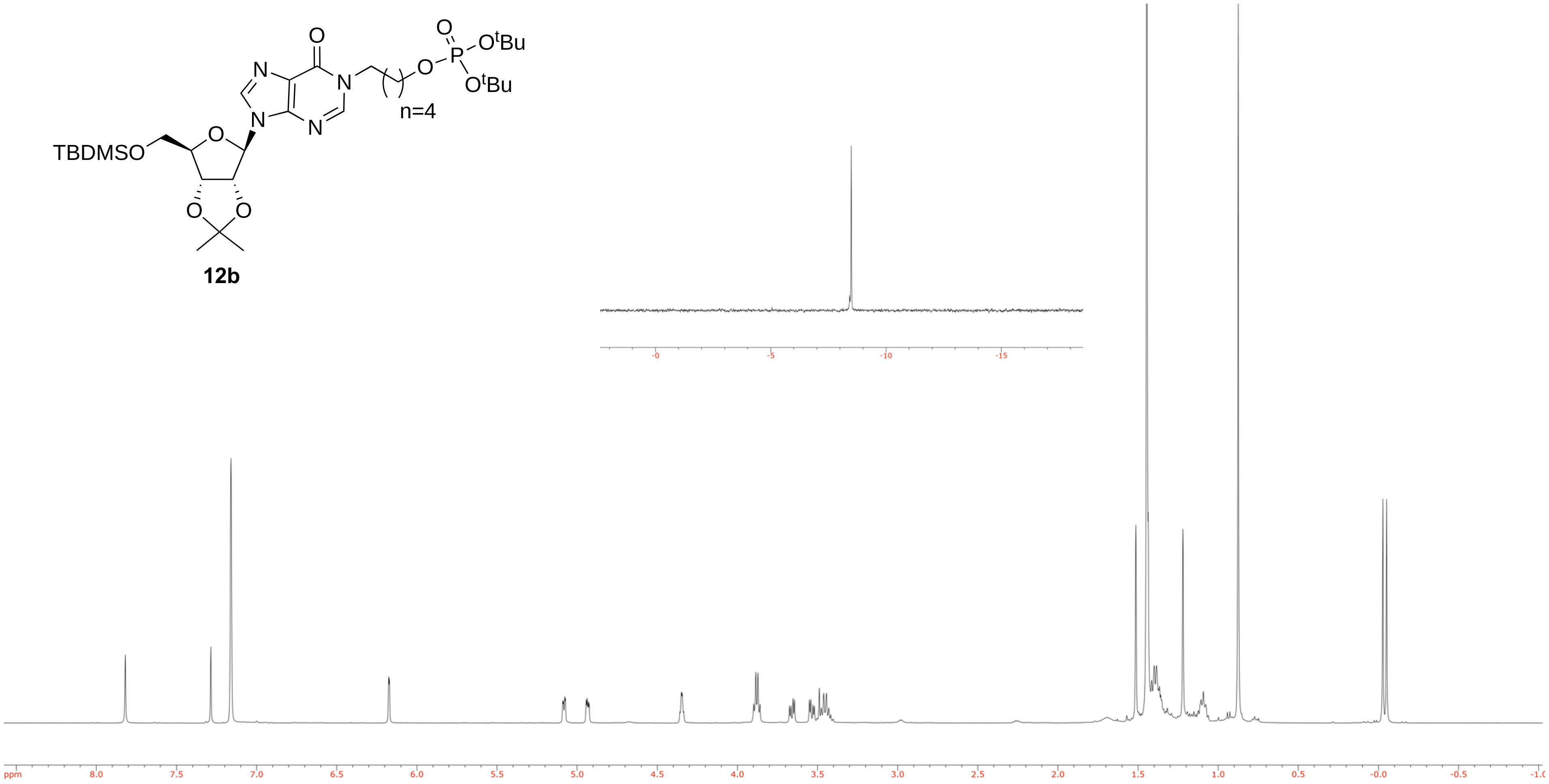
O
O
OtBu
OtBu
P
N
N
N
N
n=4
TBDMSO
O
O
O
12b
-0
-5
-10
-15
ppm
8.0
7.5
7.0
6.5
6.0
5.5
5.0
4.5
4.0
3.5
3.0
2.5
2.0
1.5
1.0
0.5
-0.0
-0.5

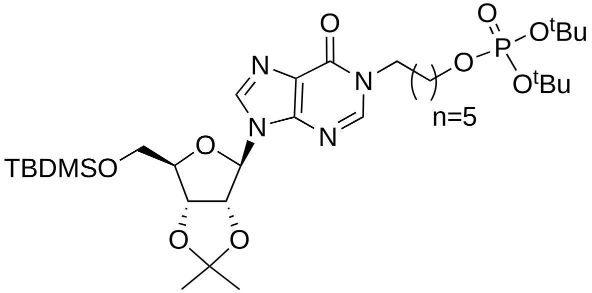

12c

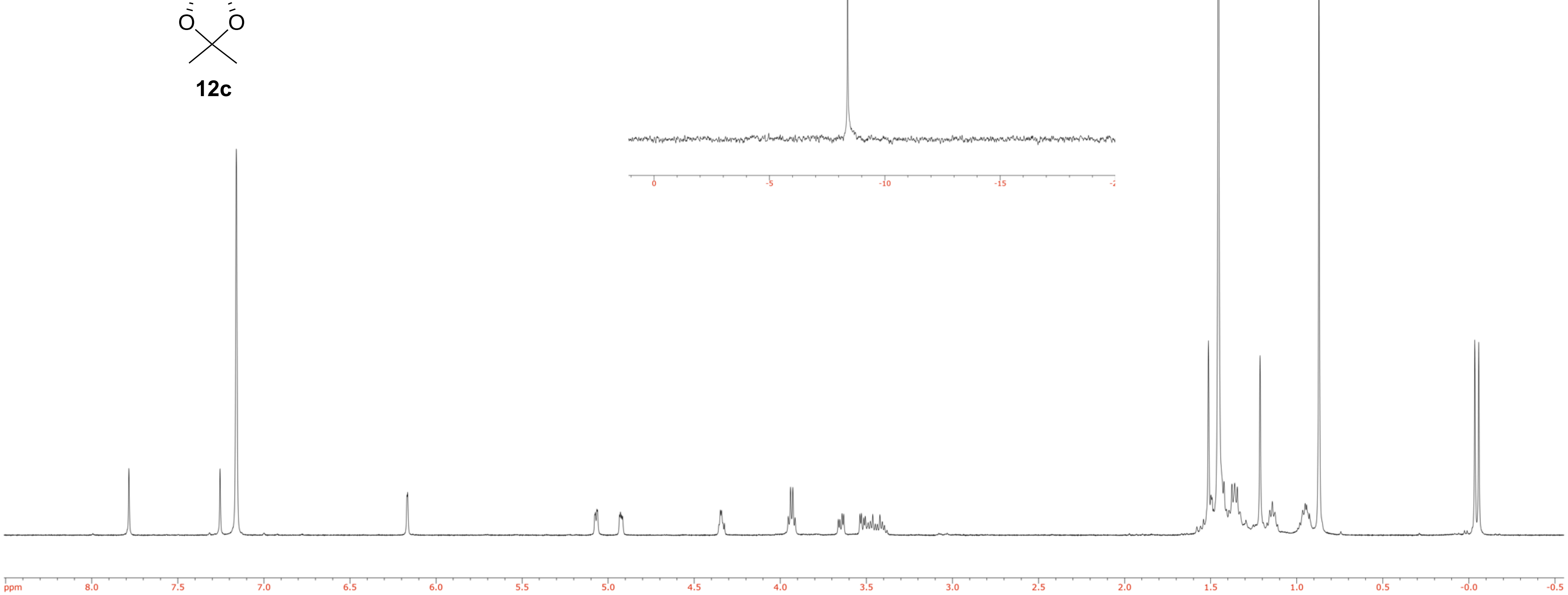

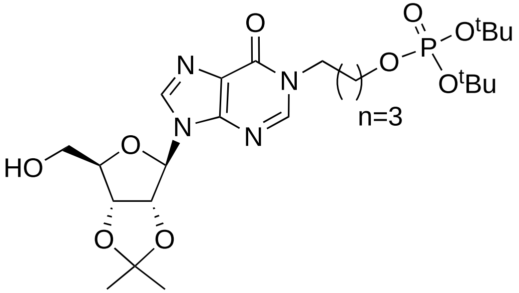
O
N
N
N
N
O
P
O
O^{t}Bu
O^{t}Bu
n=3
HO
O
O
O

13a

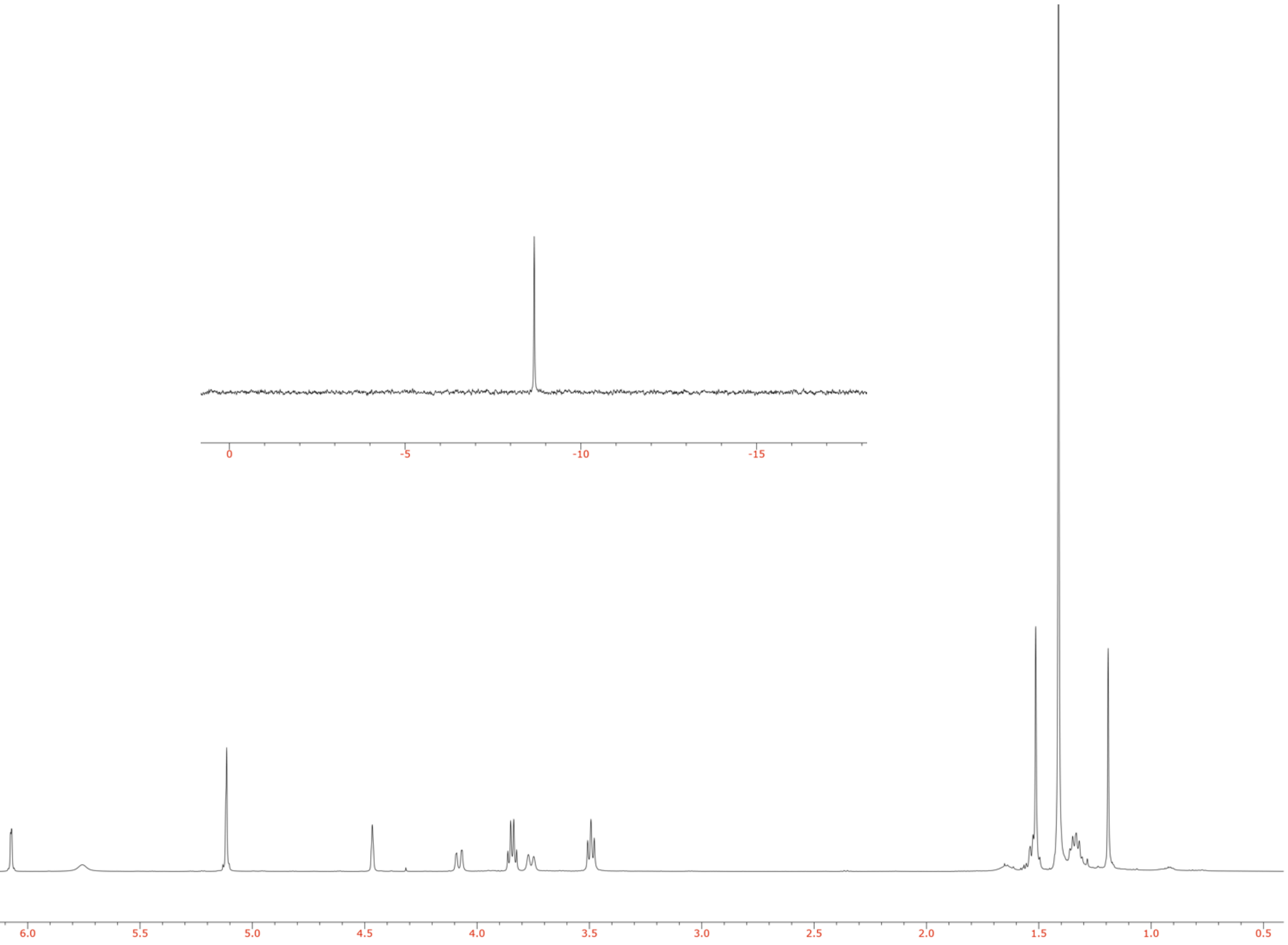
0
-5
-10
-15
ppm 8.5
8.0
7.5
7.0
6.5
6.0
5.5
5.0
4.5
4.0
3.5
3.0
2.5
2.0
1.5
1.0
0.5

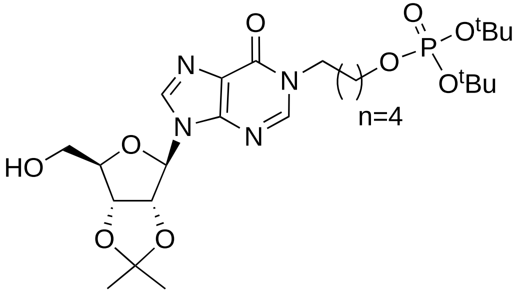

13b

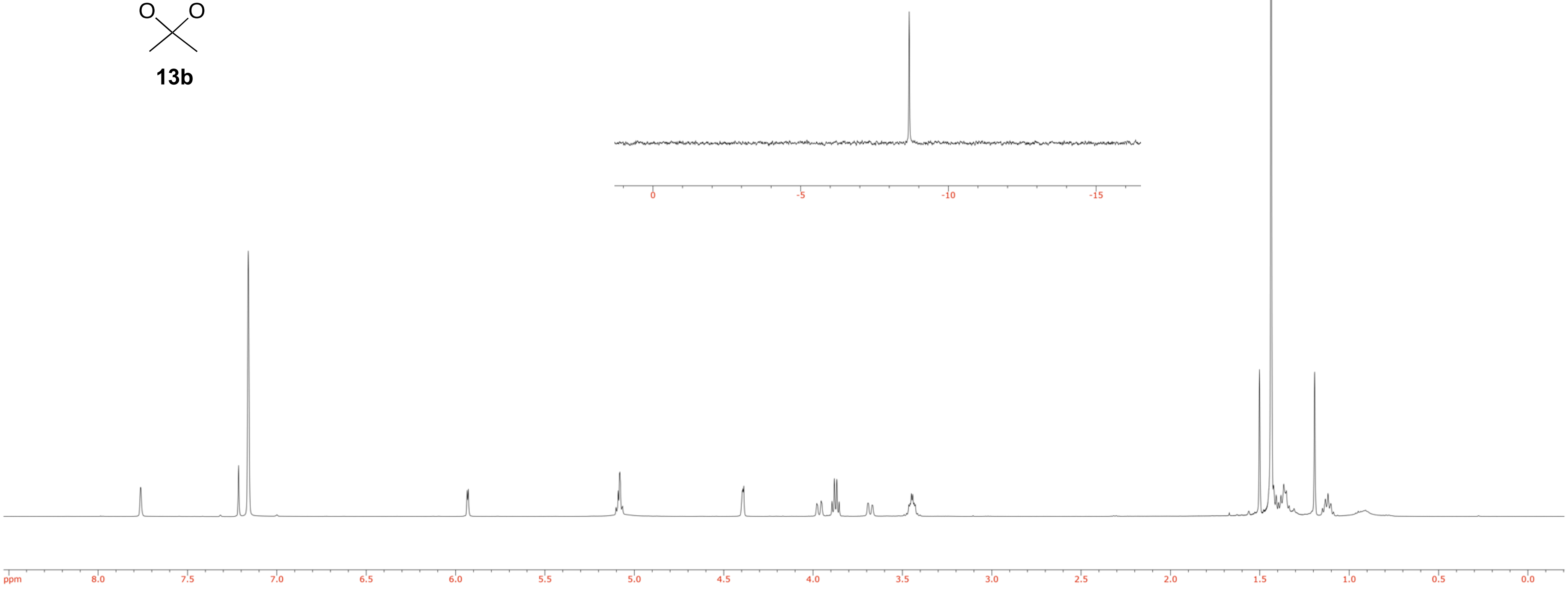

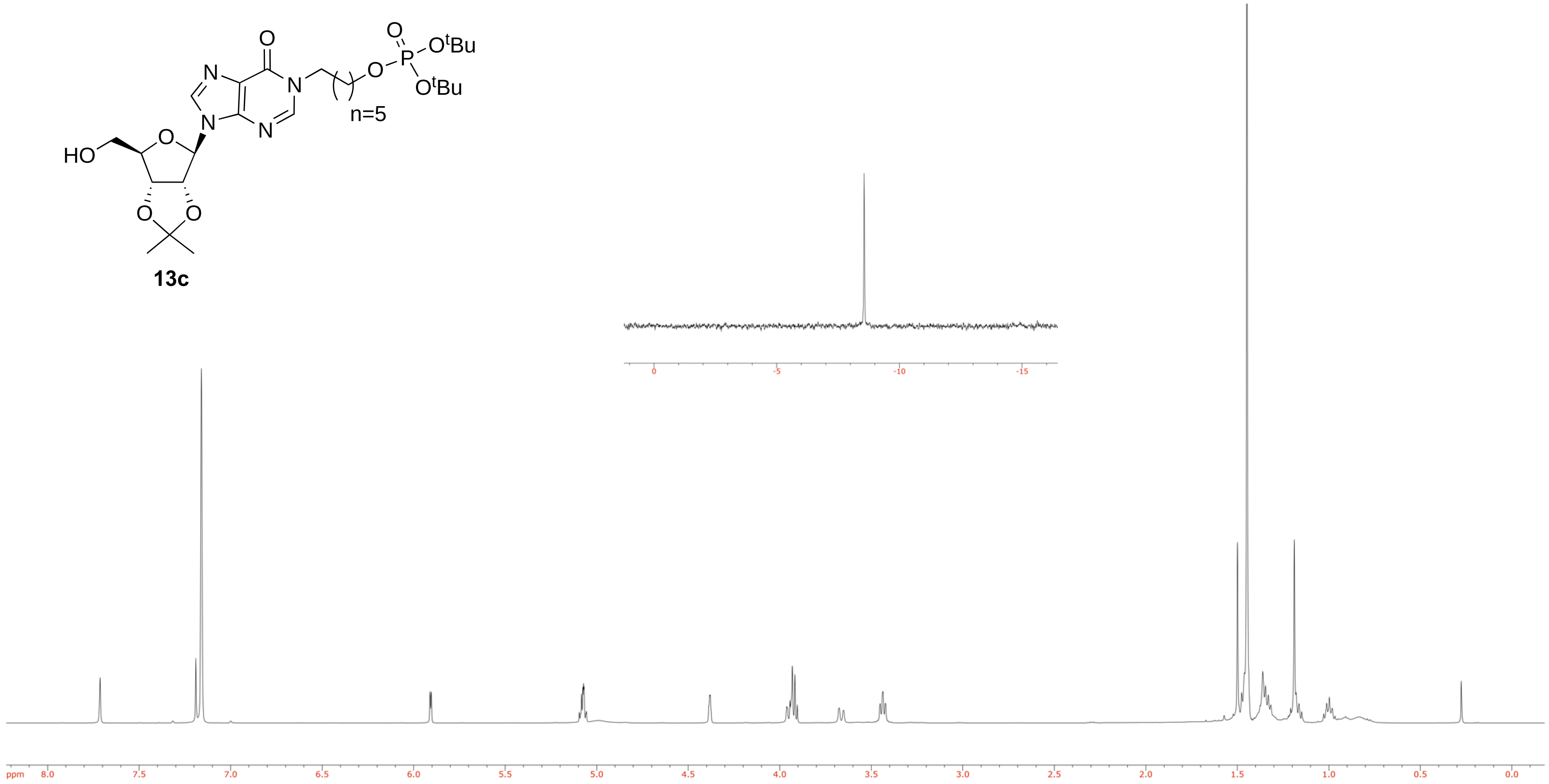

13c

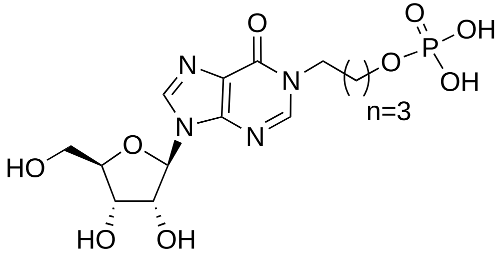
O
O
P
OH
N
N
O
OH
N
n=3
HO
O
N
N
HO
OH

14a

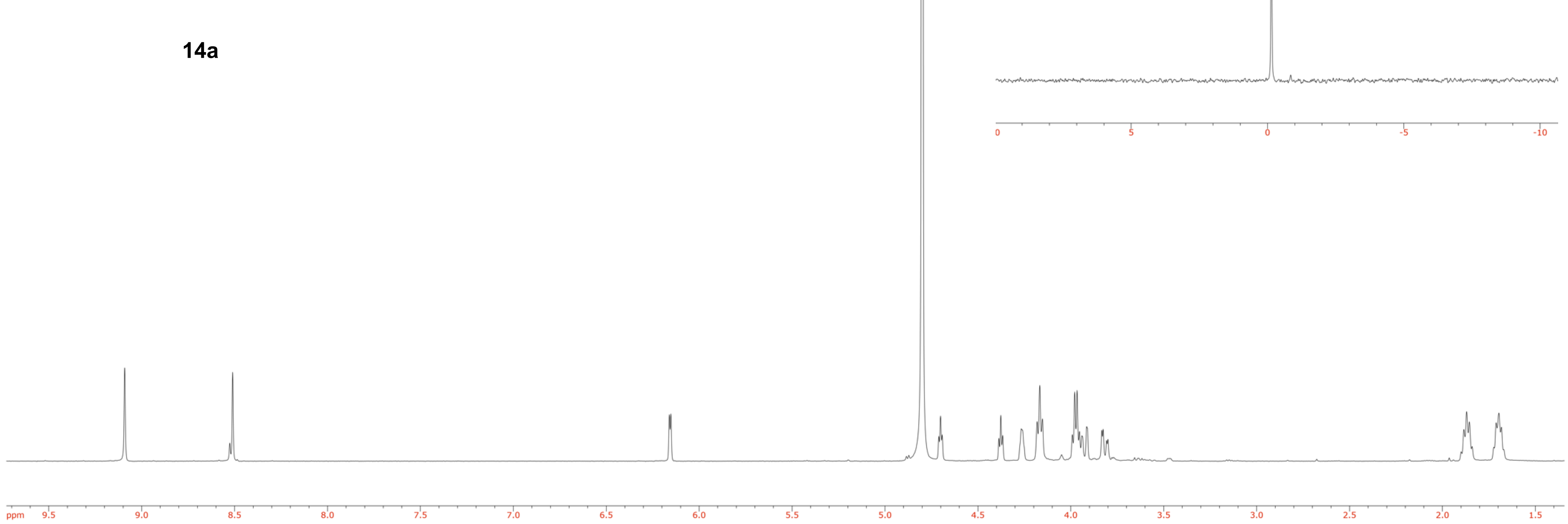
0
5
0
-5
-10
ppm
9.5
9.0
8.5
8.0
7.5
7.0
6.5
6.0
5.5
5.0
4.5
4.0
3.5
3.0
2.5
2.0
1.5

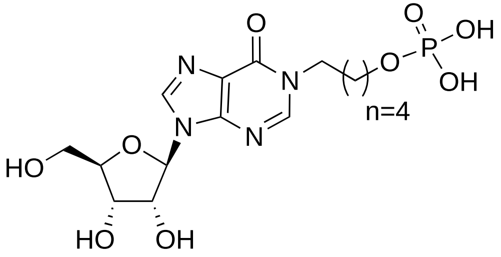

14b

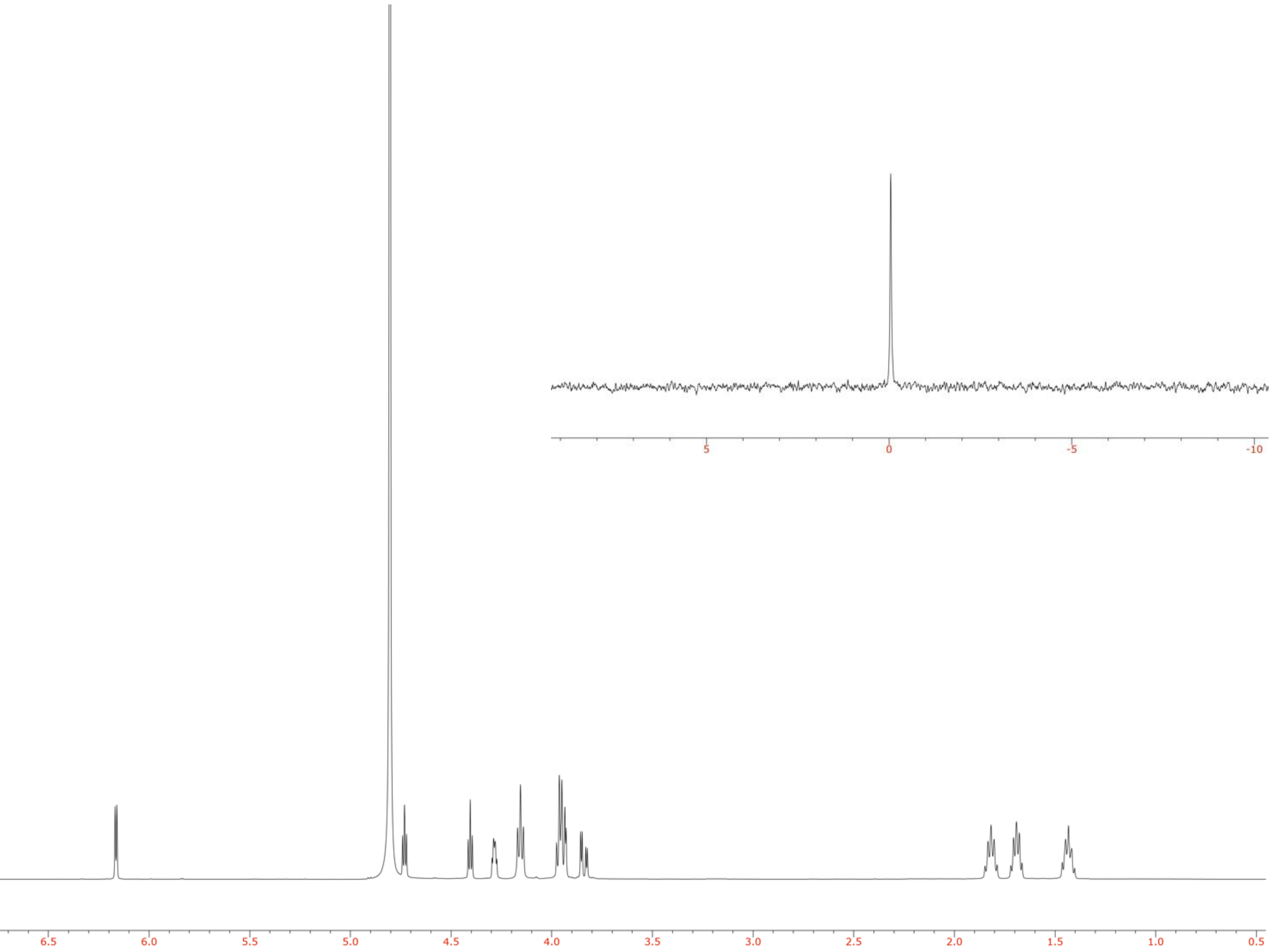

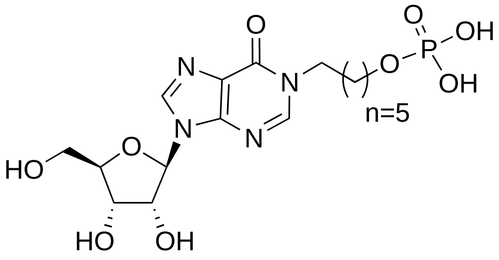

14c

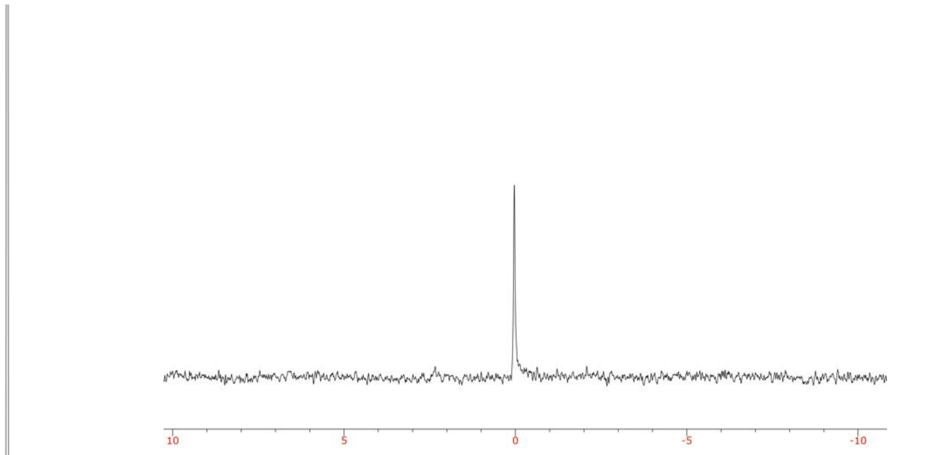

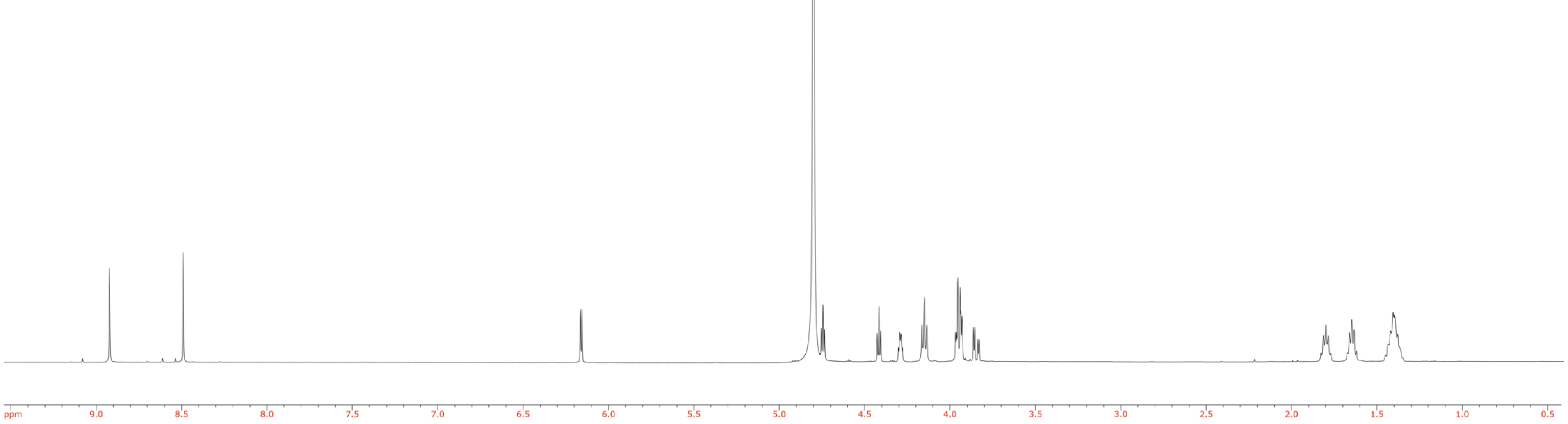

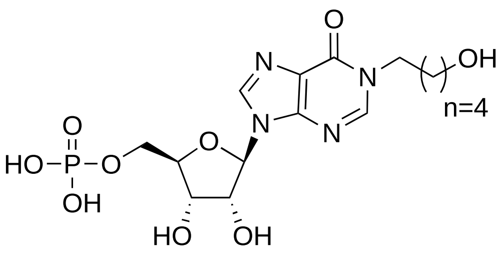

16

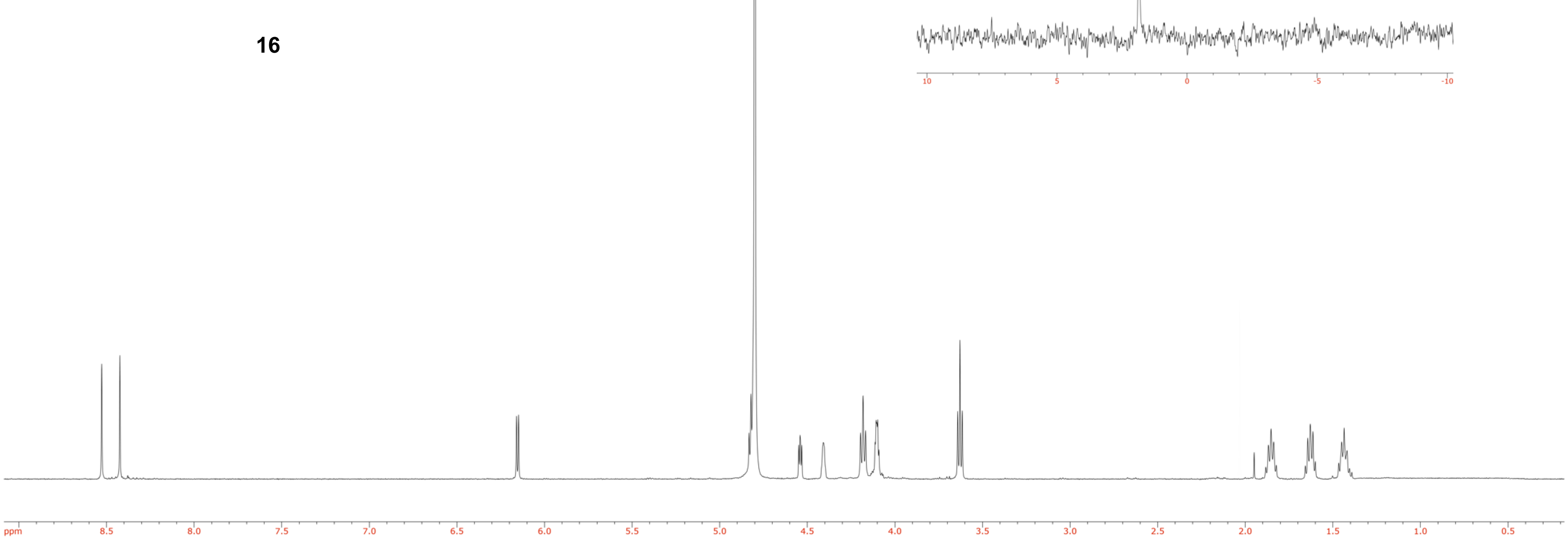

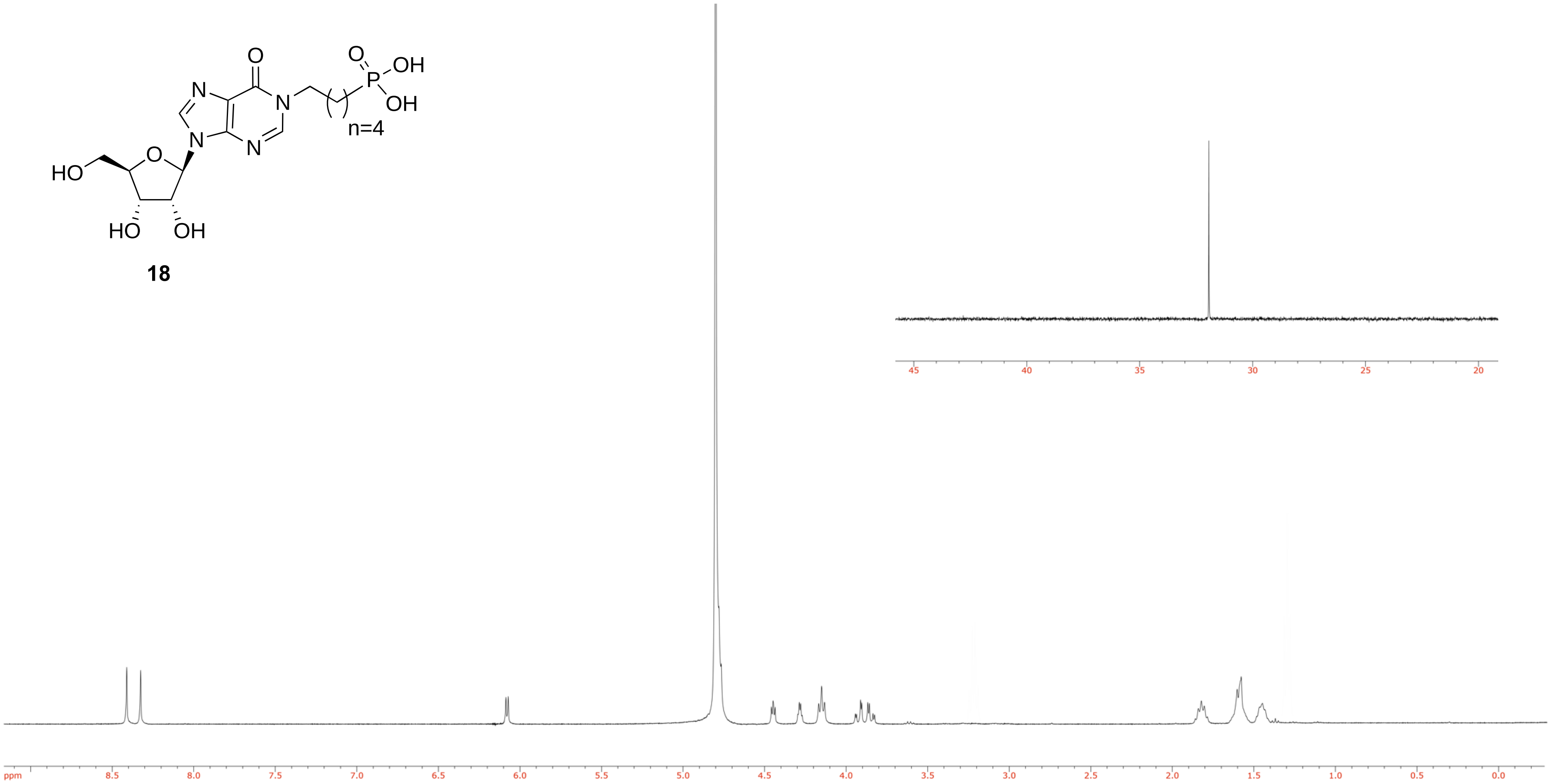
O
N
N
N
N
O
P
OH
OH
n=4
HO
HO
OH

18

45
40
35
30
25
20
ppm
8.5
8.0
7.5
7.0
6.5
6.0
5.5
5.0
4.5
4.0
3.5
3.0
2.5
2.0
1.5
1.0
0.5
0.0

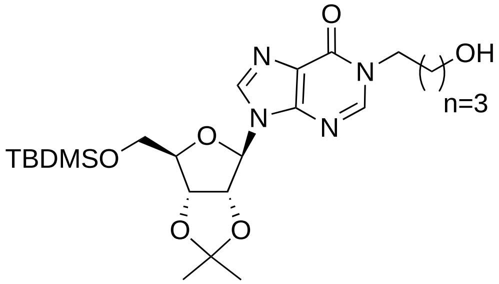
TBDMSO
OH
n=3

8a

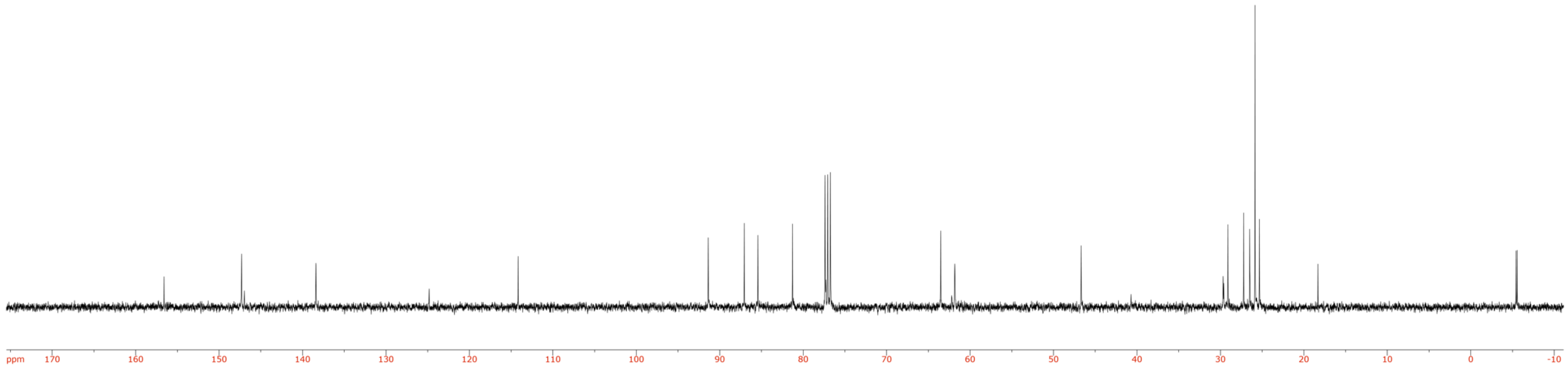
ppm 170 160 150 140 130 120 110 100 90 80 70 60 50 40 30 20 10 0 -10

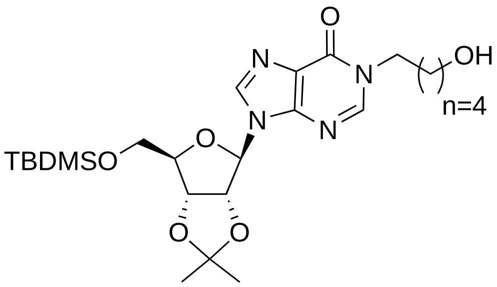
TBDMSO
O
N
OH
n=4

8b

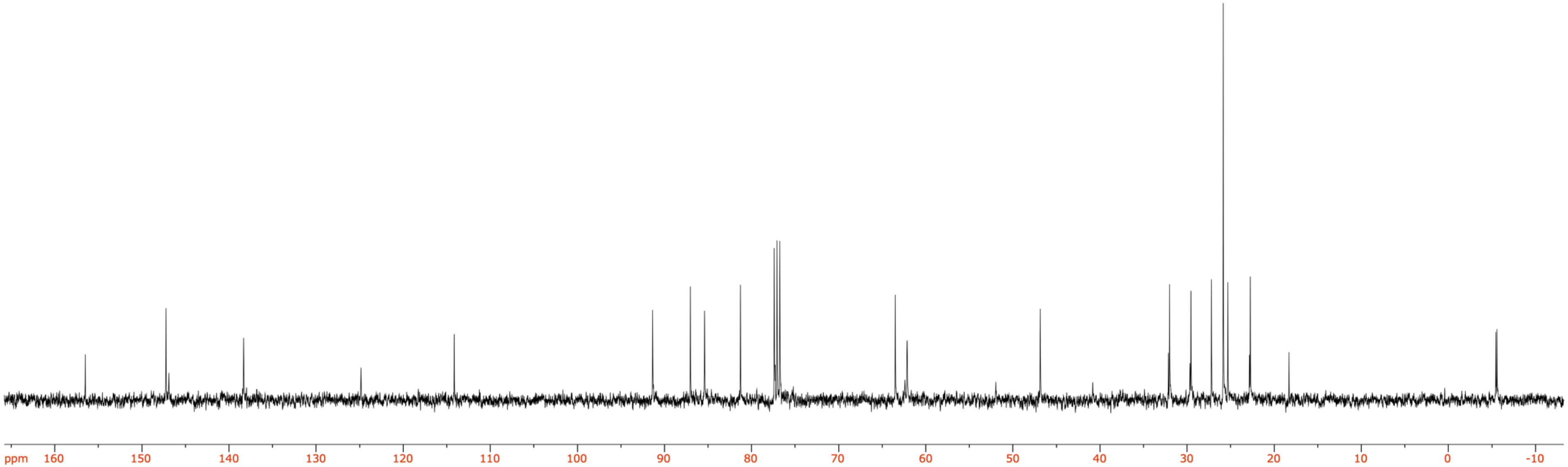
ppm 160 150 140 130 120 110 100 90 80 70 60 50 40 30 20 10 0 -10

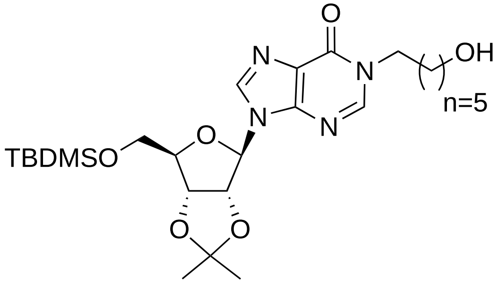
O
N
N
OH
N
N
n=5
TBDMSO
O
O
O

8c

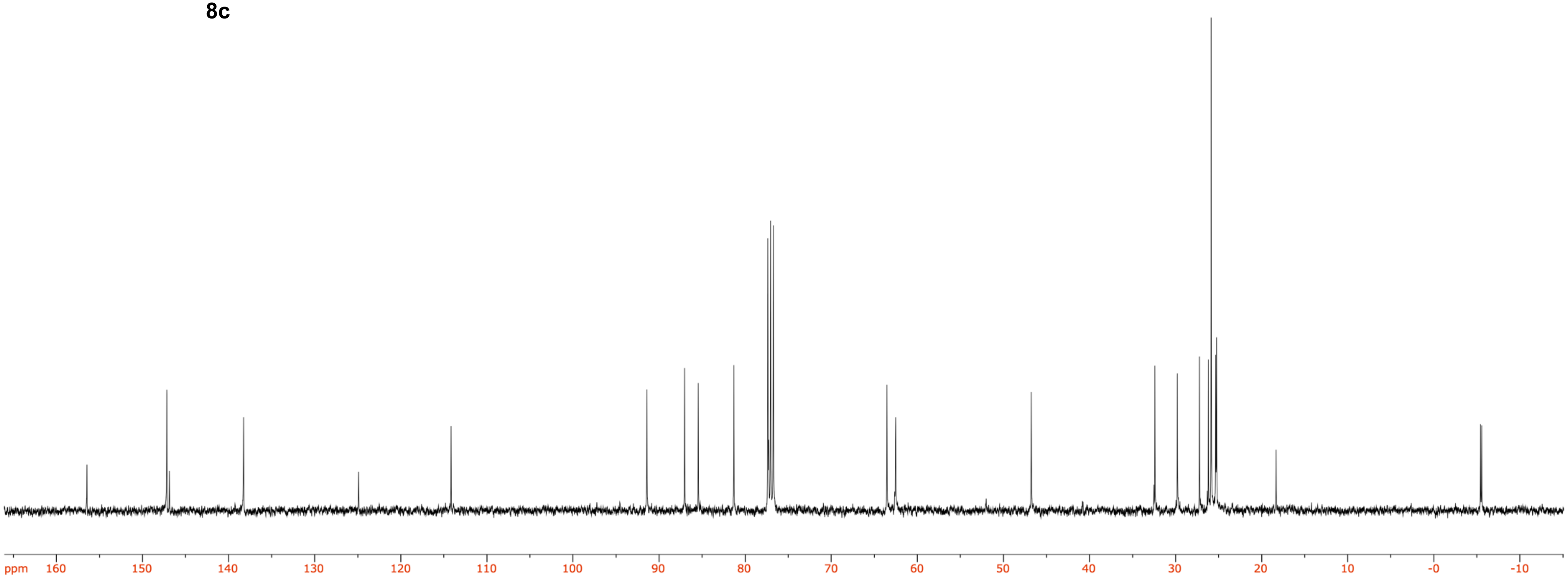
ppm 160 150 140 130 120 110 100 90 80 70 60 50 40 30 20 10 -0 -10

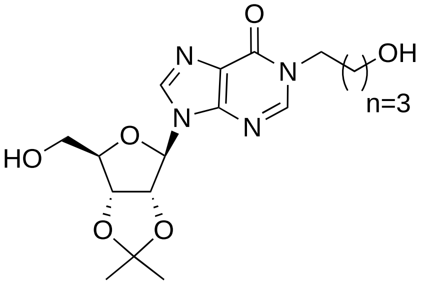
O
N
N
OH
n=3
N
N
O
HO
O
O

9a

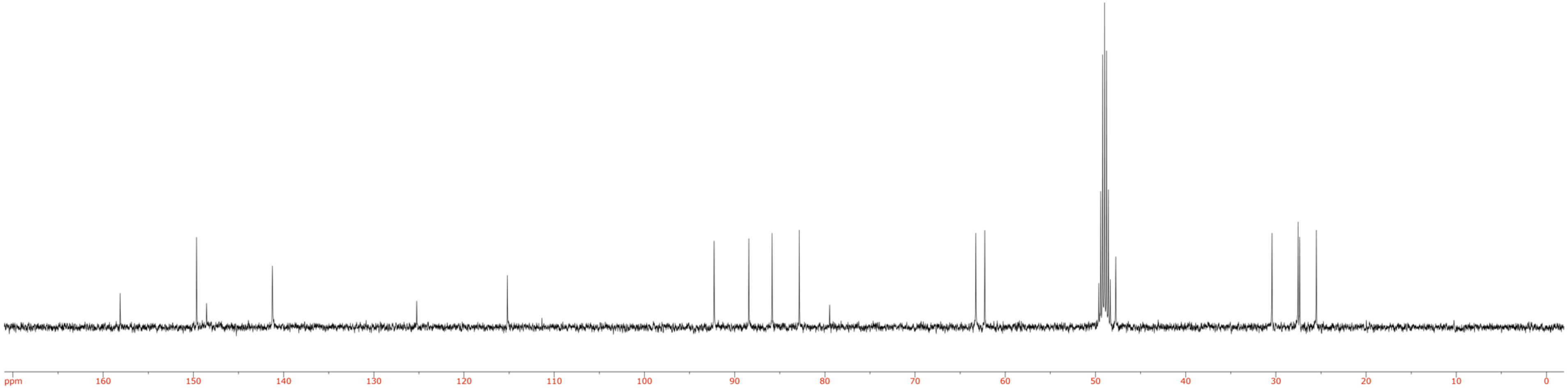
ppm
160
150
140
130
120
110
100
90
80
70
60
50
40
30
20
10
0

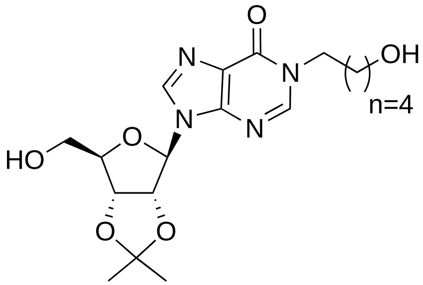

9b

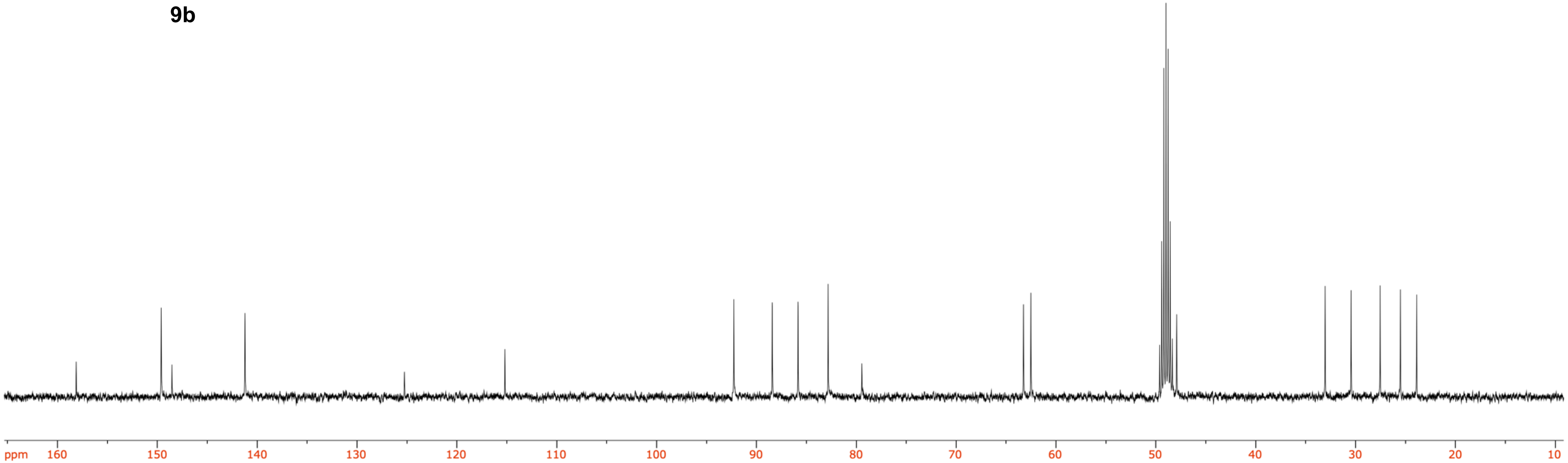

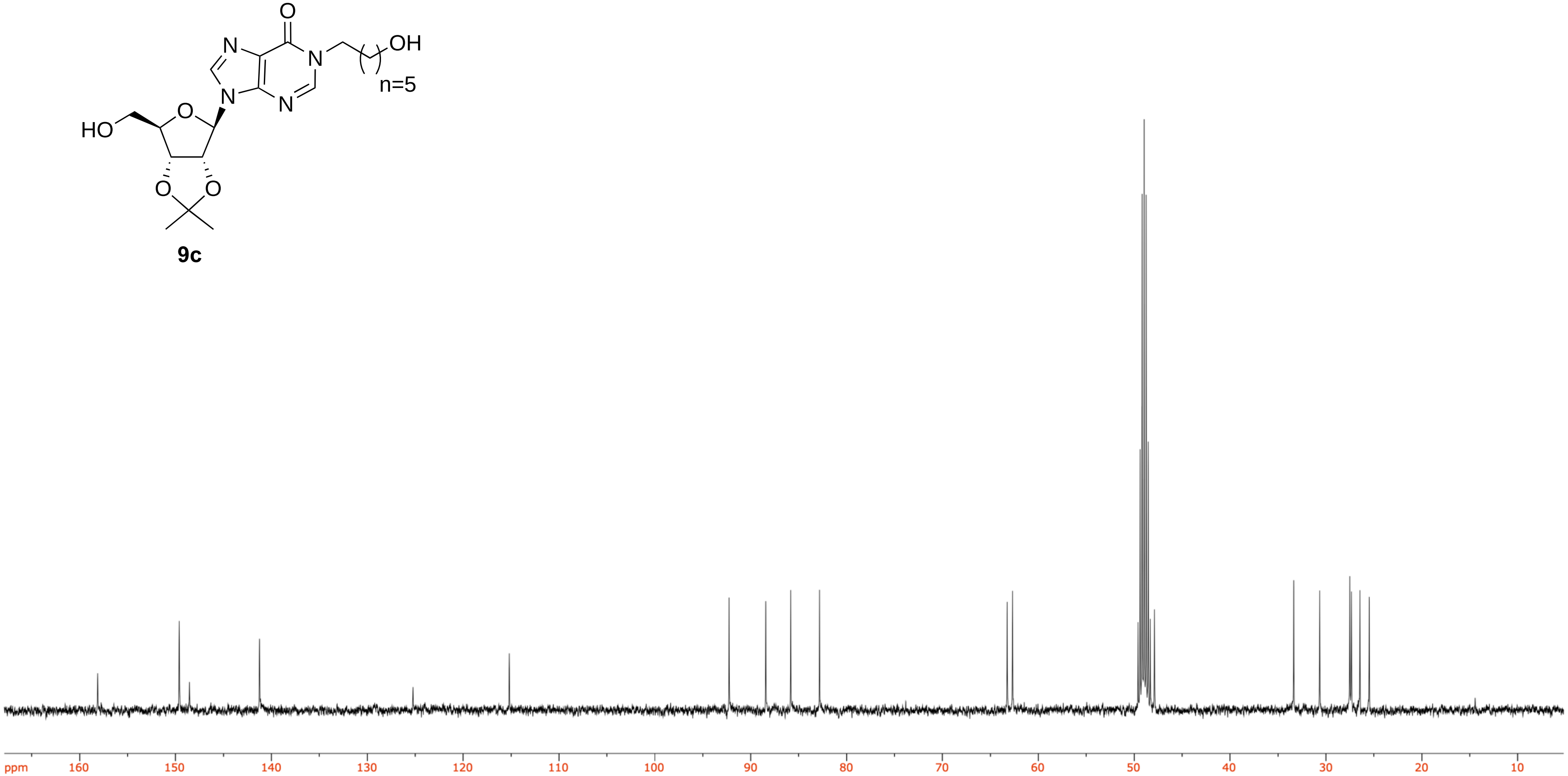
O
N
N
N
N
OH
n=5
HO
O
O
O
9c
ppm
160
150
140
130
120
110
100
90
80
70
60
50
40
30
20
10

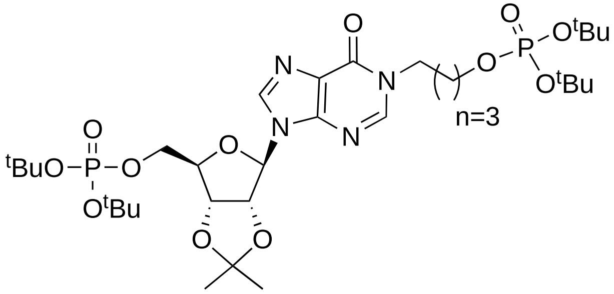
O
N
N
N
N
O
O
O
O
P
O
O
OᵗBu
OᵗBu
ᵗBuO
OᵗBu
P
O
n=3

10a

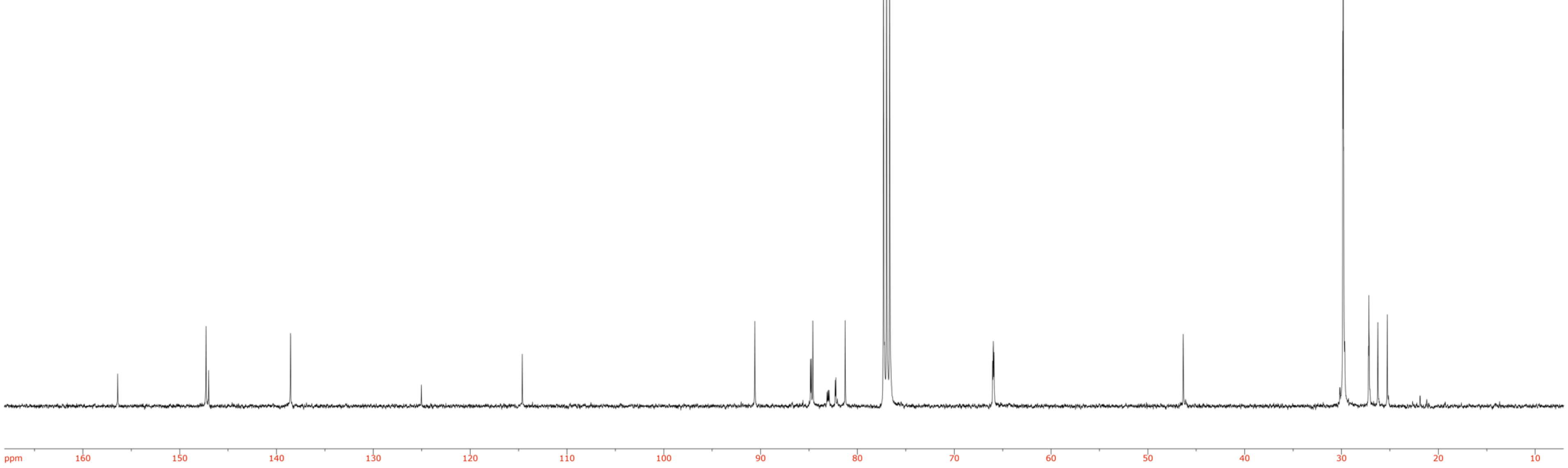
ppm
160
150
140
130
120
110
100
90
80
70
60
50
40
30
20
10

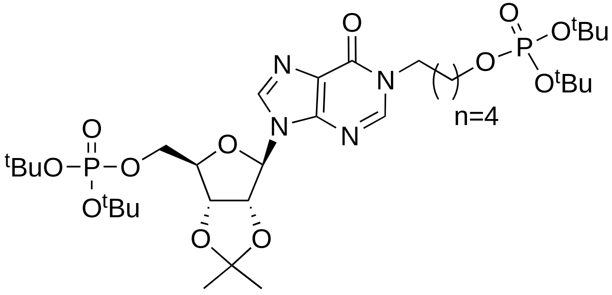
O
N
N
N
N
O
n=4
O
P
O^{t}Bu
O^{t}Bu
^{t}BuO
P
O
O
O^{t}Bu
O
O
O

10b

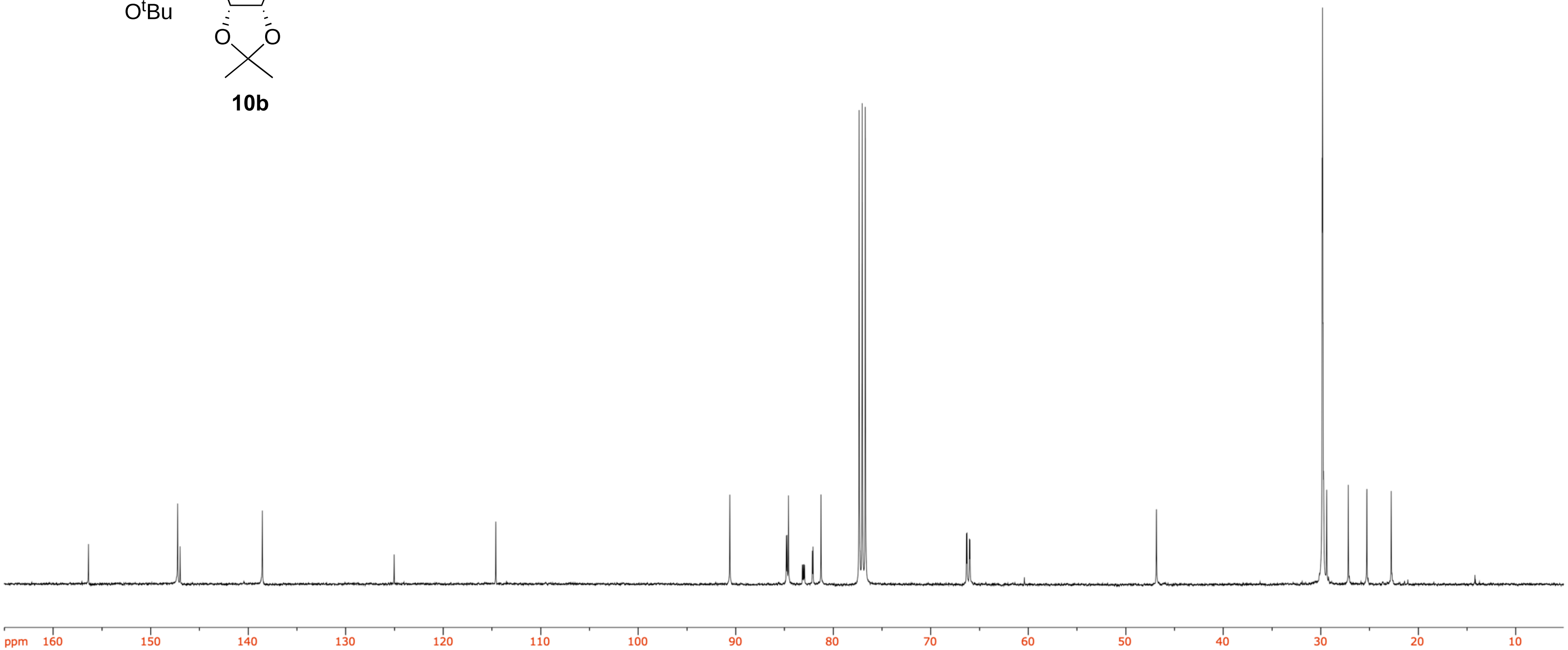
ppm 160 150 140 130 120 110 100 90 80 70 60 50 40 30 20 10

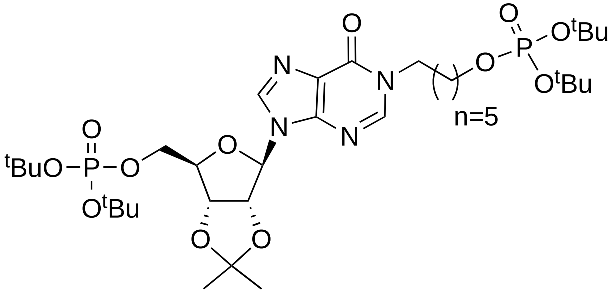
O
N
N
N
N
O
P
O^tBu
O^tBu
n=5
^tBuO
P
O
O
O^tBu
O
O
O

10c

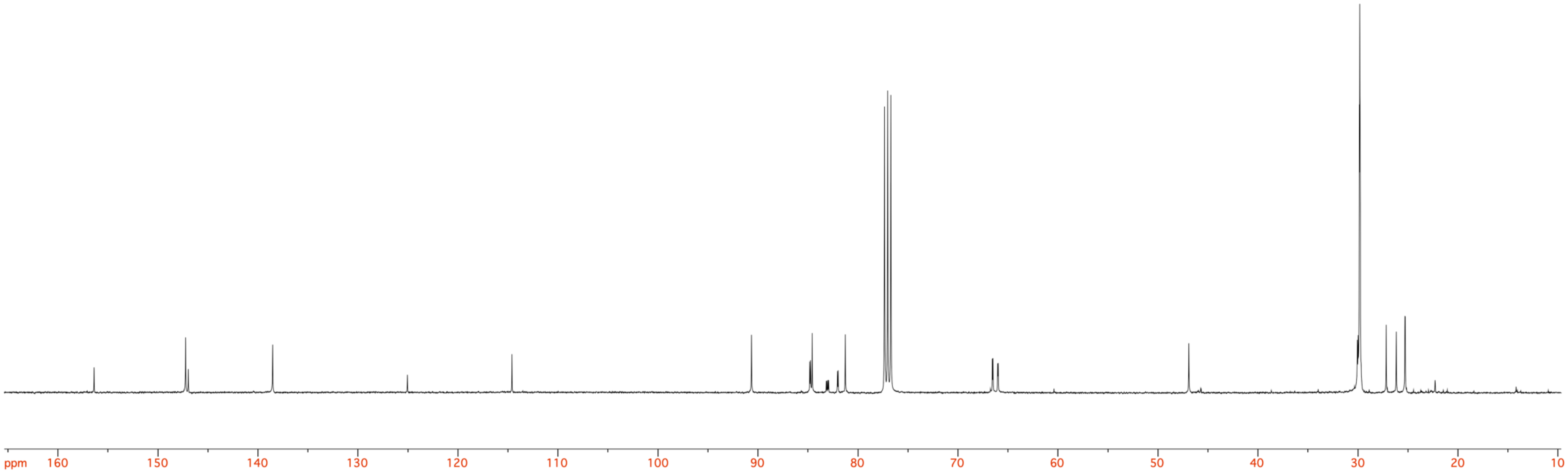
ppm 160 150 140 130 120 110 100 90 80 70 60 50 40 30 20 10

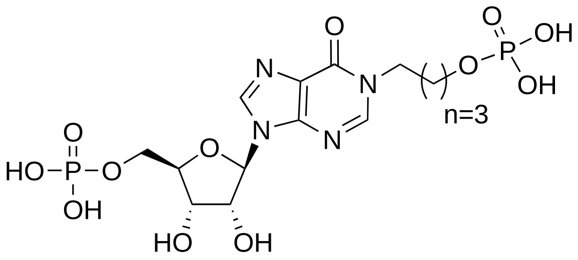

HO
OH
O
O
N
N
N
N
O
n=3
OH

11a

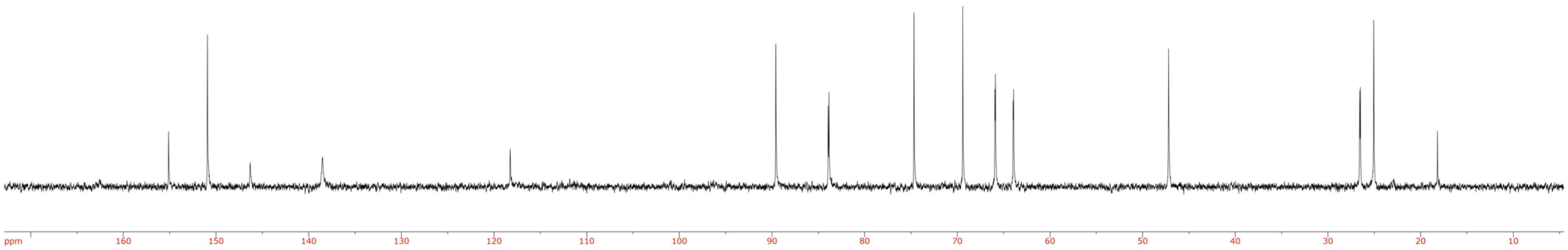

ppm
160
150
140
130
120
110
100
90
80
70
60
50
40
30
20
10

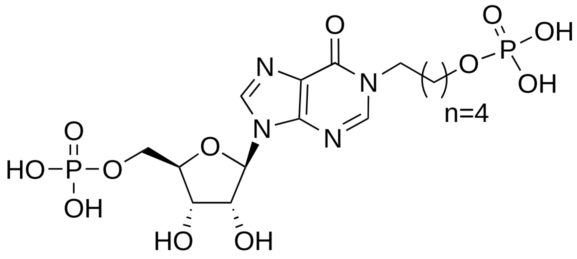

11b

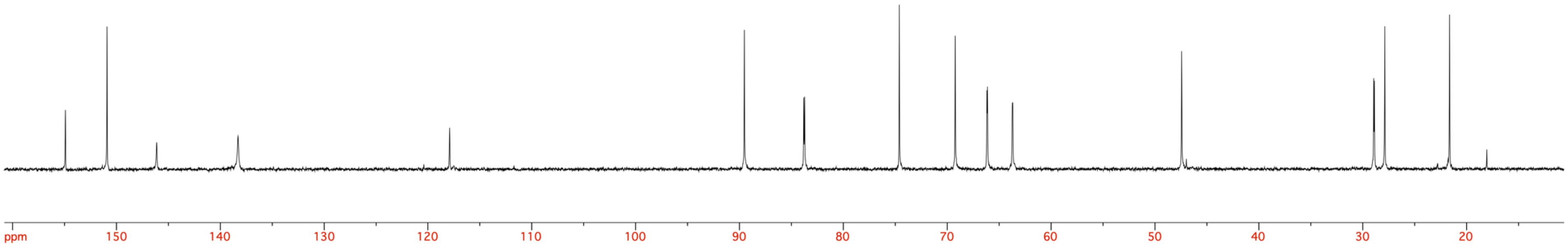

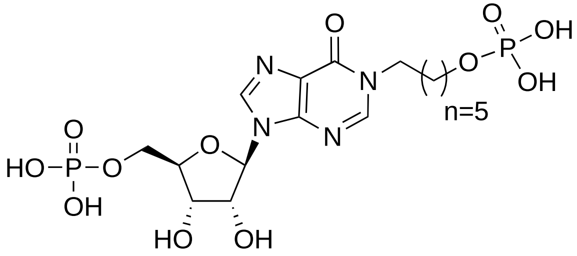

O
OH
N
O
P
N
OH
n=5
O
N
N
HO
P
O
O
OH
HO
OH

11c

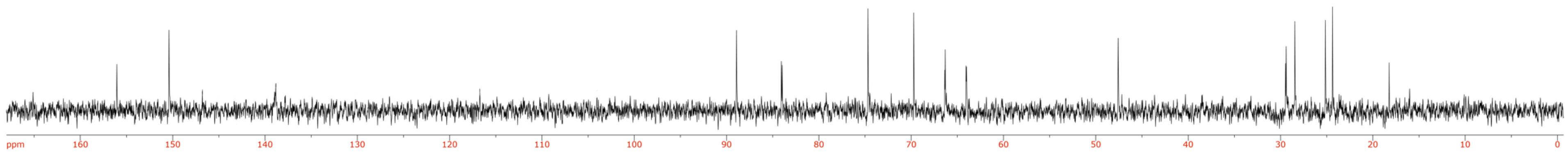

ppm
160
150
140
130
120
110
100
90
80
70
60
50
40
30
20
10
0

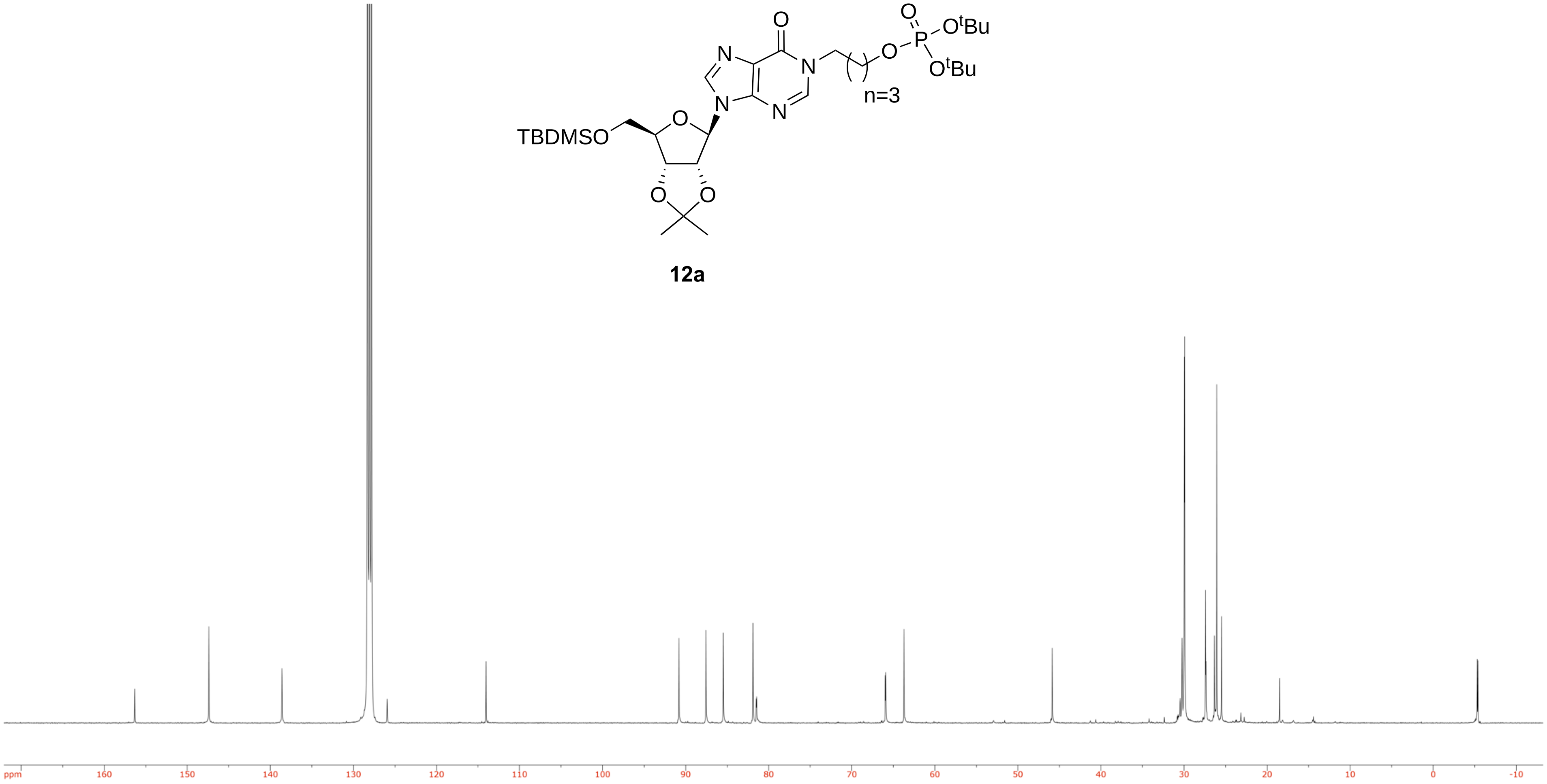
TBDMSO
n=3
OtBu
OtBu
12a
ppm 160 150 140 130 120 110 100 90 80 70 60 50 40 30 20 10 0 -10

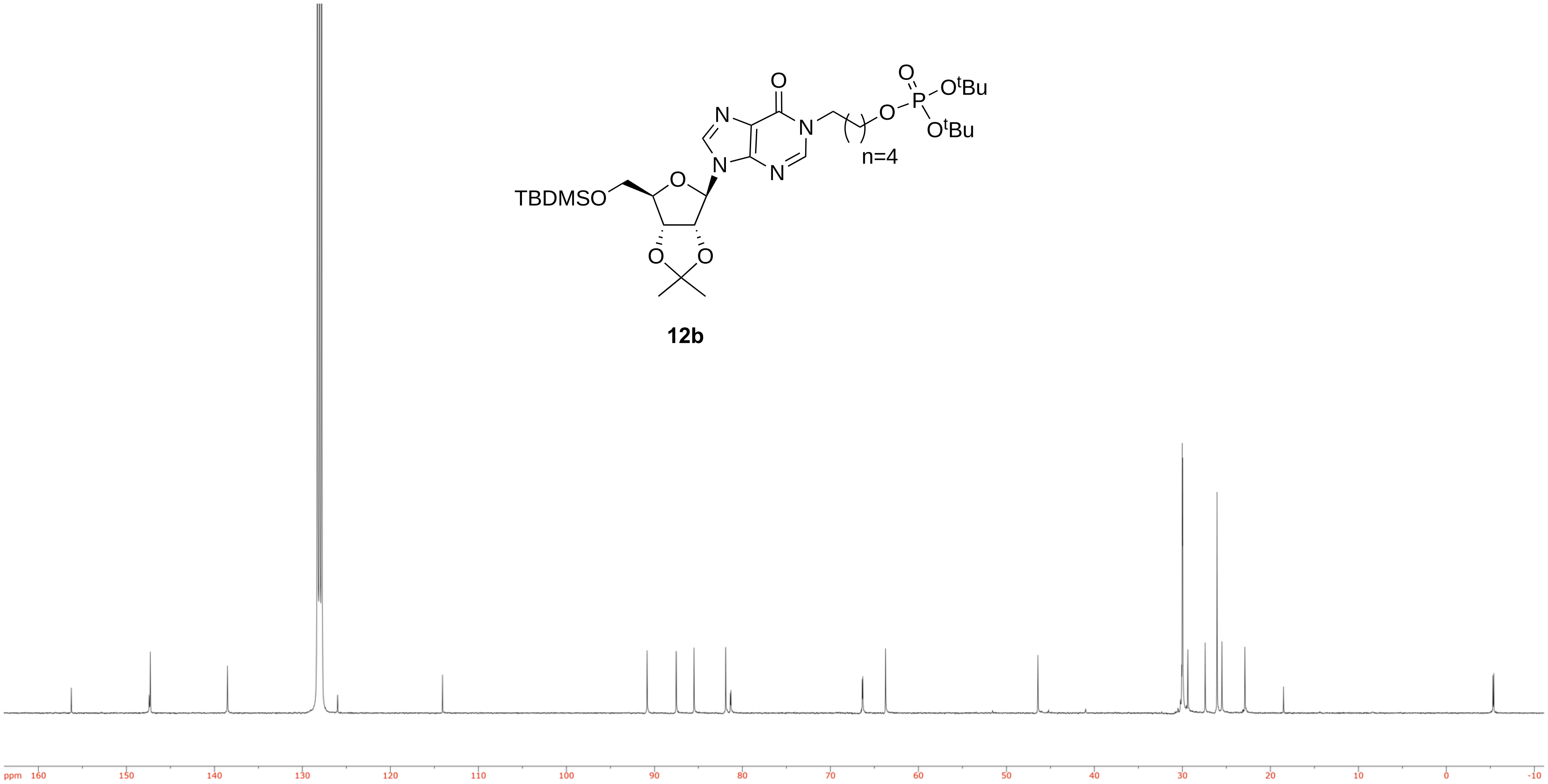
O
N
N
N
N
O
O
O
TBDMSO
O
O
P
O^{t}Bu
O^{t}Bu
n=4
ppm 160 150 140 130 120 110 100 90 80 70 60 50 40 30 20 10 0 -10

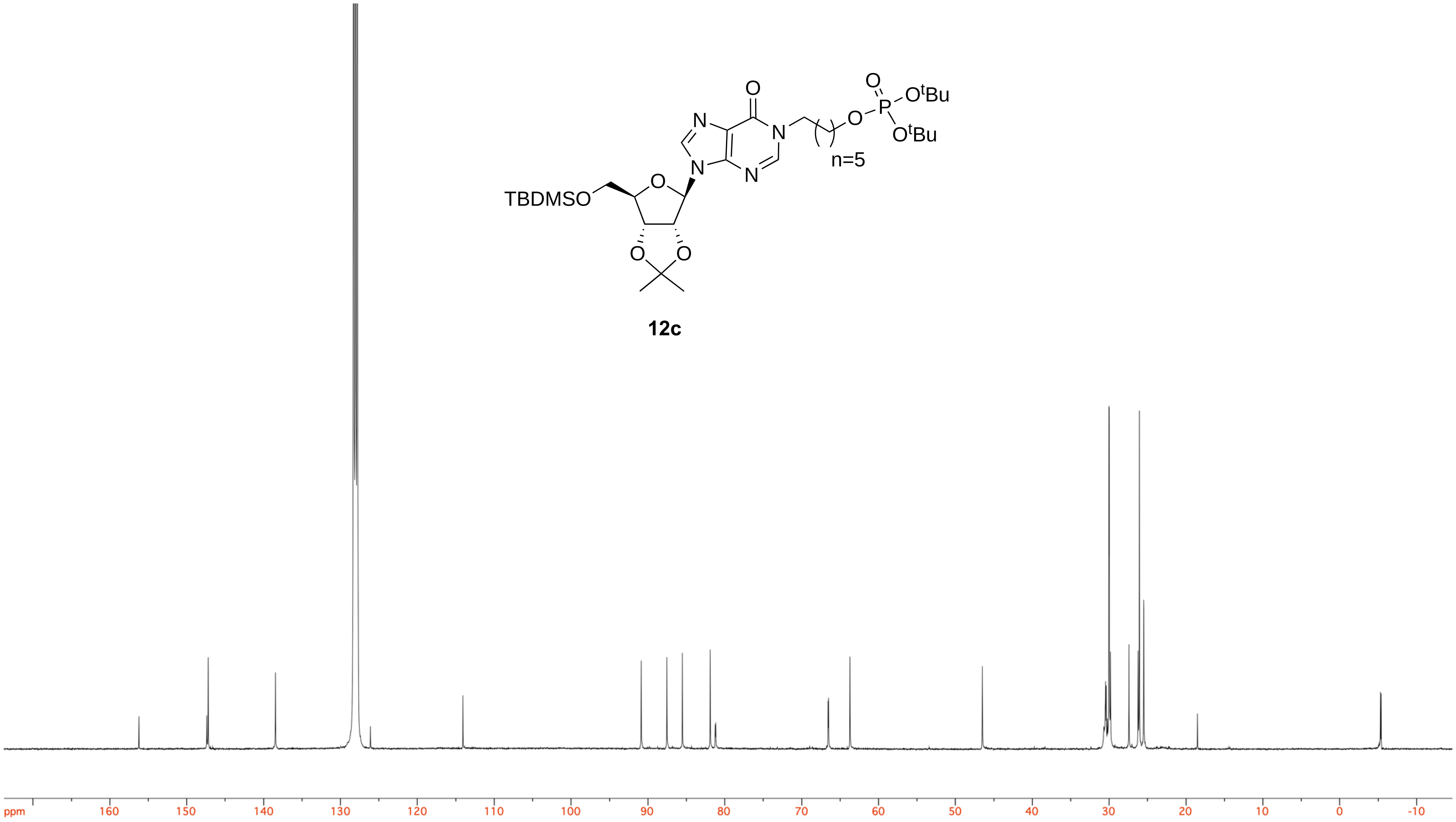
O
N
N
N
N
O
P
O
OtBu
OtBu
n=5
TBDMSO
O
O
O
12c
ppm
160
150
140
130
120
110
100
90
80
70
60
50
40
30
20
10
0
-10

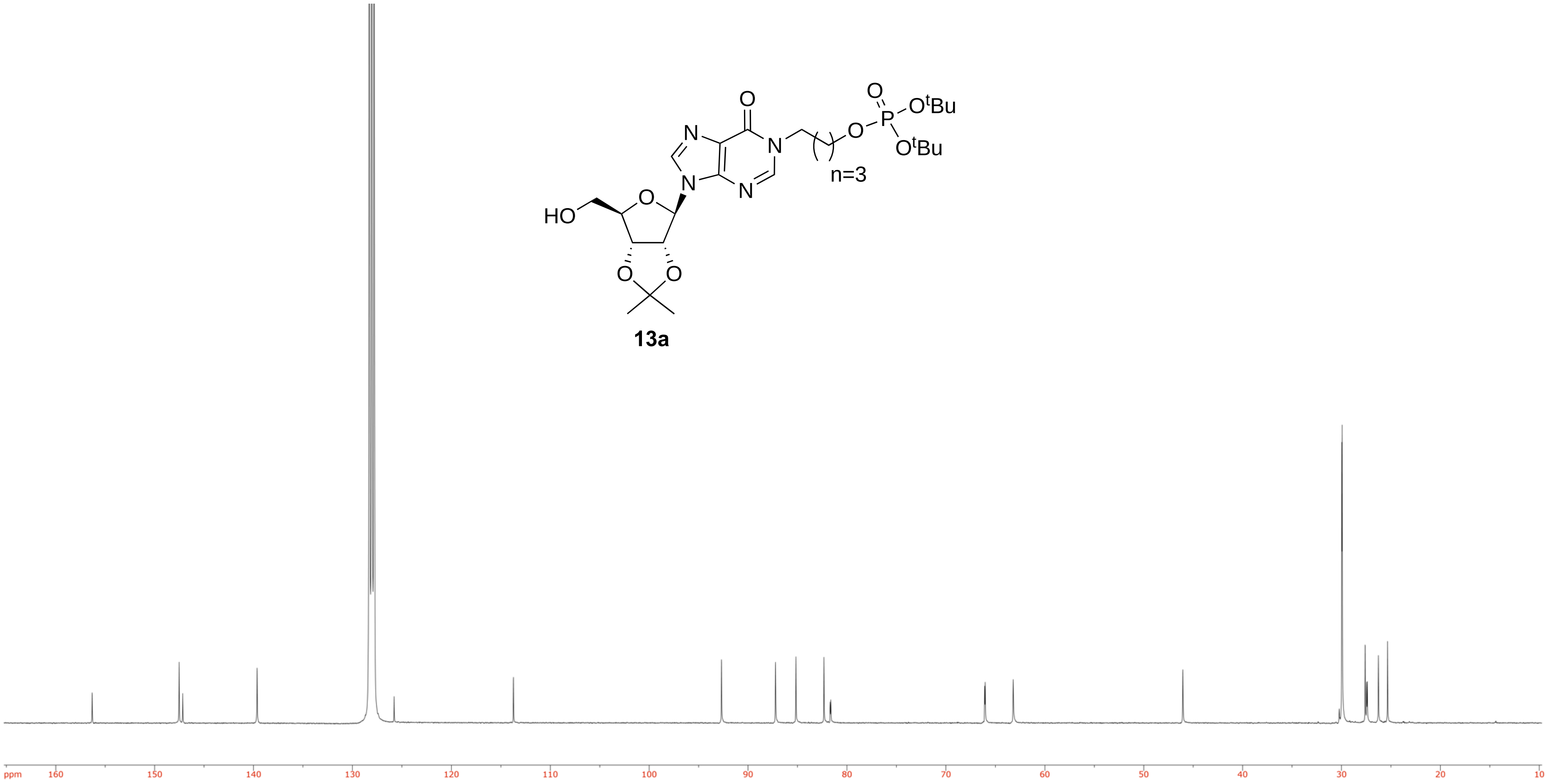

O
N
N
N
N
HO
O
O
O
O
P
O
O^{t}Bu
O^{t}Bu
n=3
13a
ppm 160 150 140 130 120 110 100 90 80 70 60 50 40 30 20 10

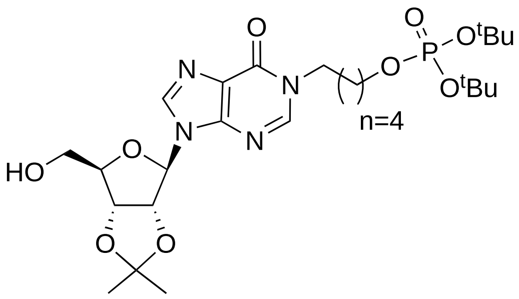

O
N
N
N
N
HO
O
O
O
O
P
O
O^{t}Bu
O^{t}Bu
n=4

13b

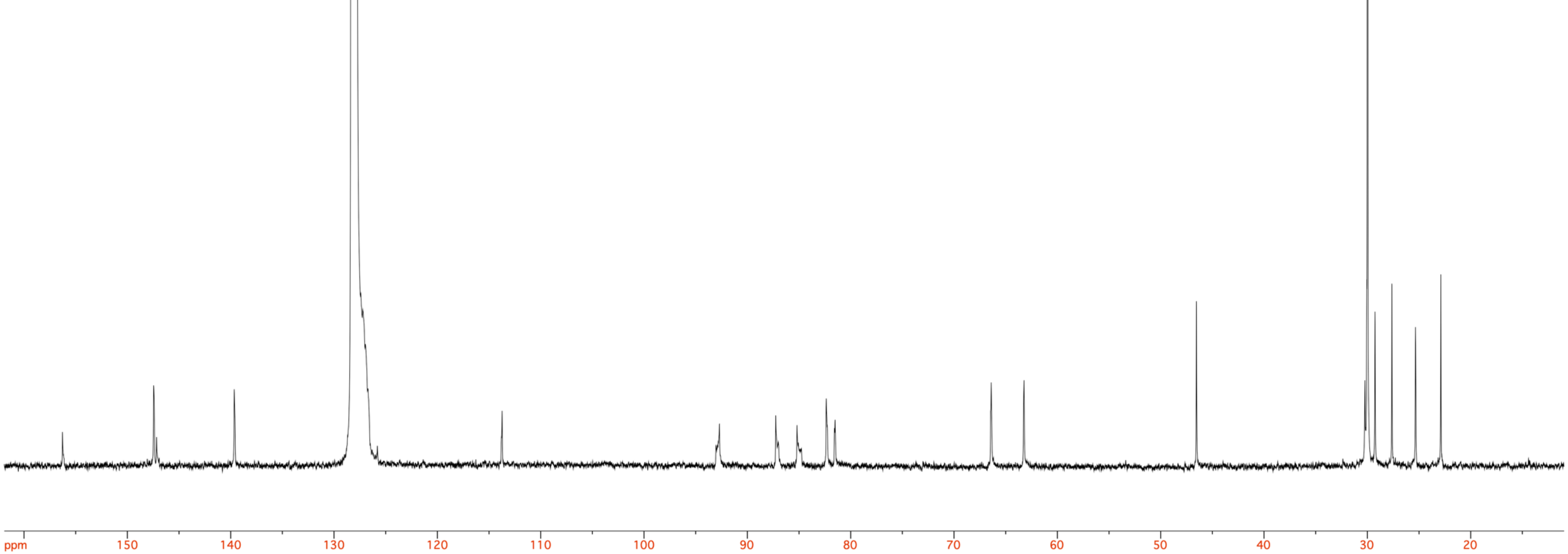

ppm
150
140
130
120
110
100
90
80
70
60
50
40
30
20

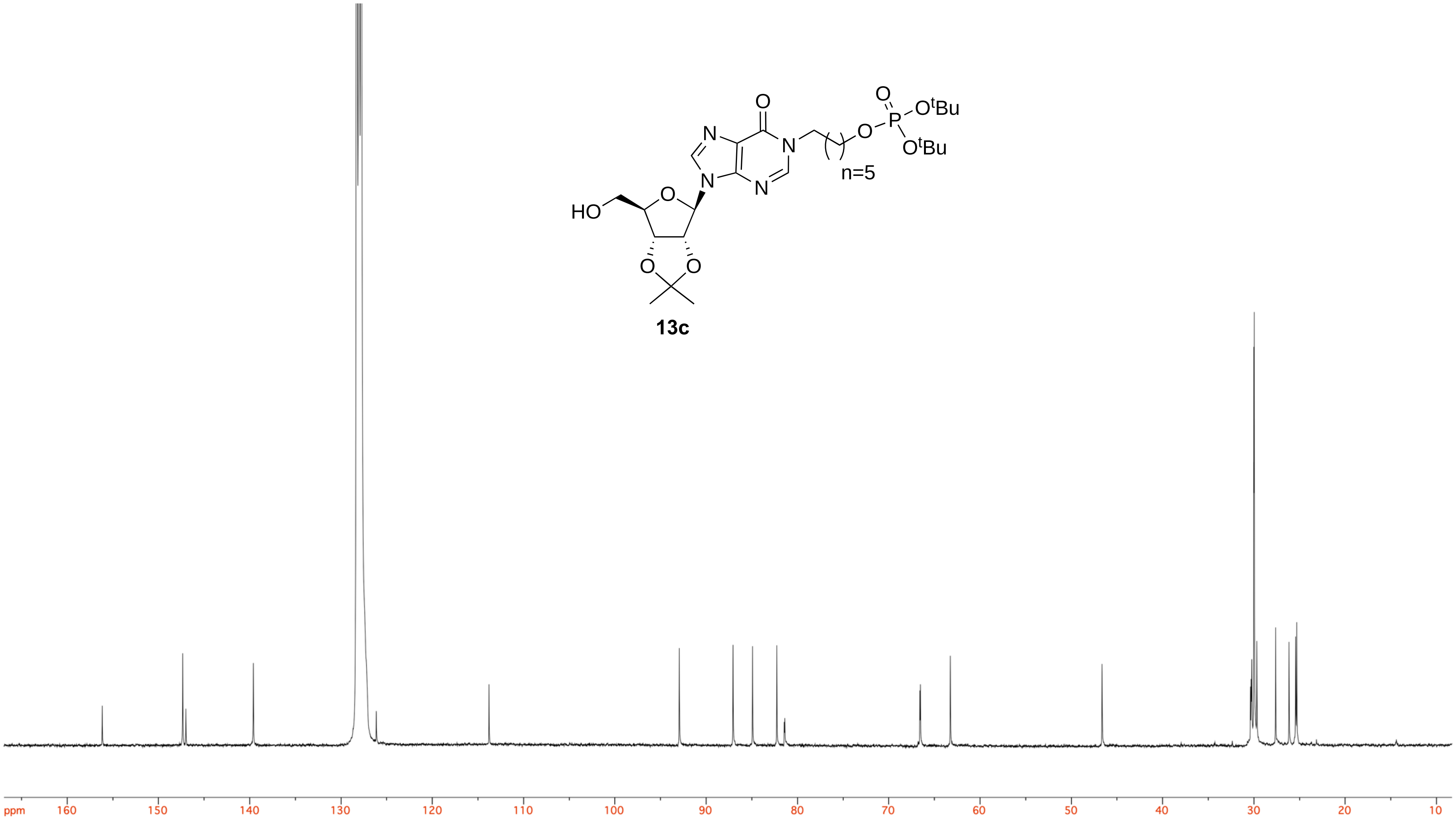
O
N
N
HO
O
N
N
O
O
n=5
O
P
O^{t}Bu
O^{t}Bu
13c
ppm
160
150
140
130
120
110
100
90
80
70
60
50
40
30
20
10

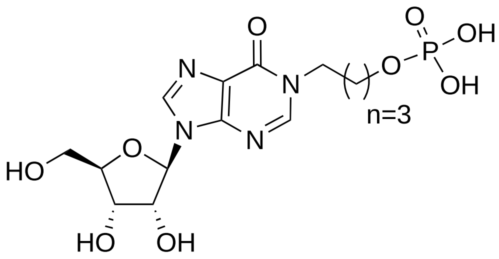

14a

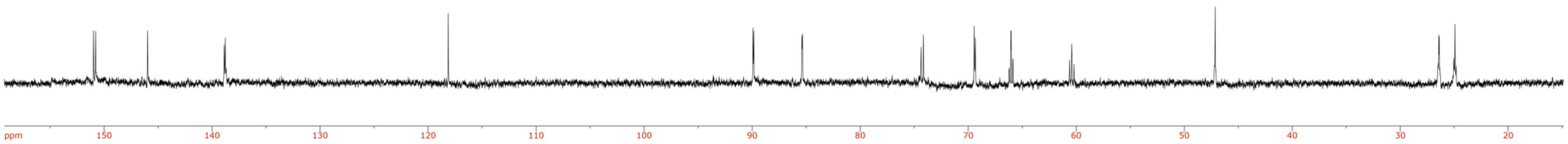

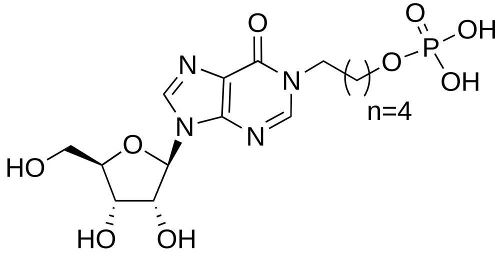
O
N
N
N
N
O
P
OH
OH
n=4
HO
O
HO
OH

14b

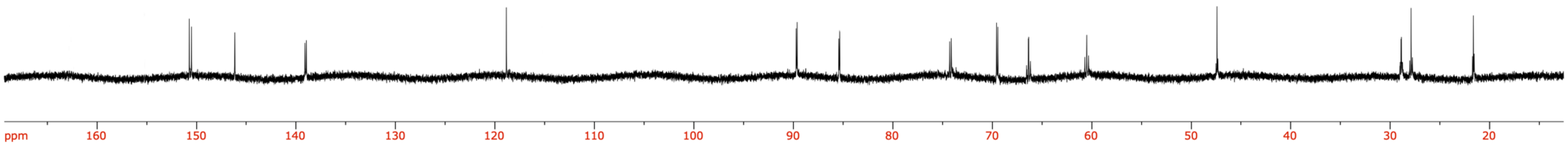
ppm
160
150
140
130
120
110
100
90
80
70
60
50
40
30
20

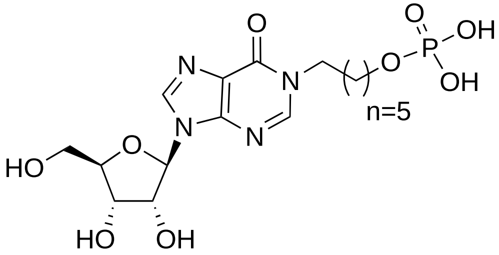
O
N
N
N
N
O
O
P
OH
OH
n=5
HO
O
HO
OH

14c

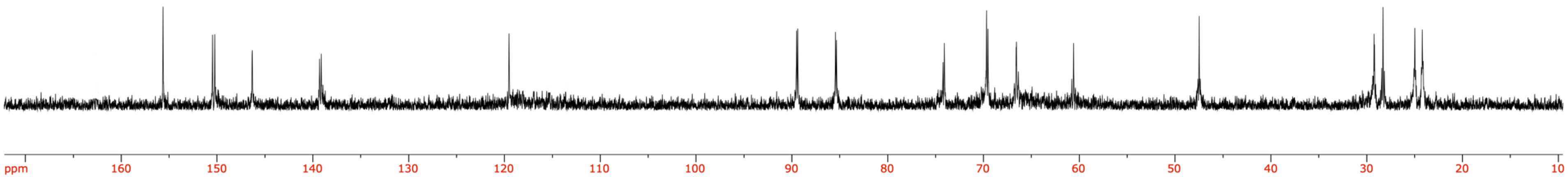
ppm
160
150
140
130
120
110
100
90
80
70
60
50
40
30
20
10

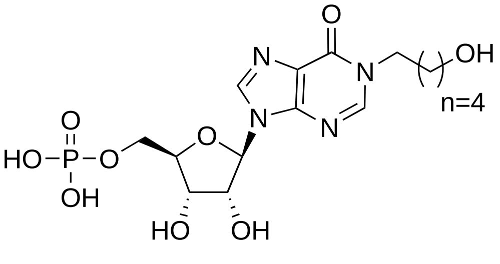

16

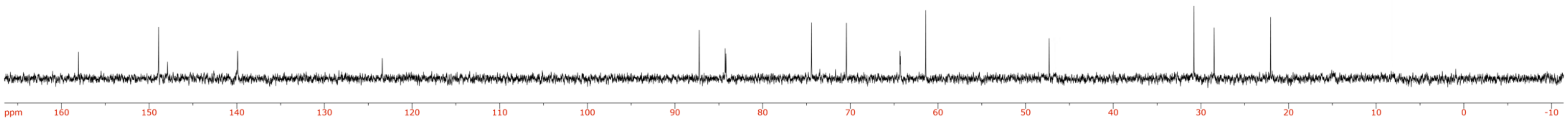

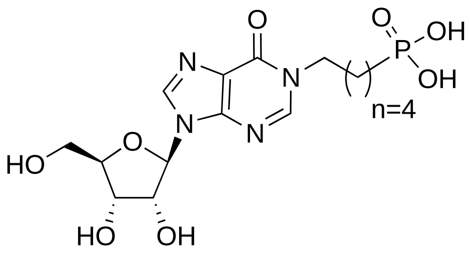

O
N
N
N
N
O
P
OH
OH
n=4
HO
HO
OH

18

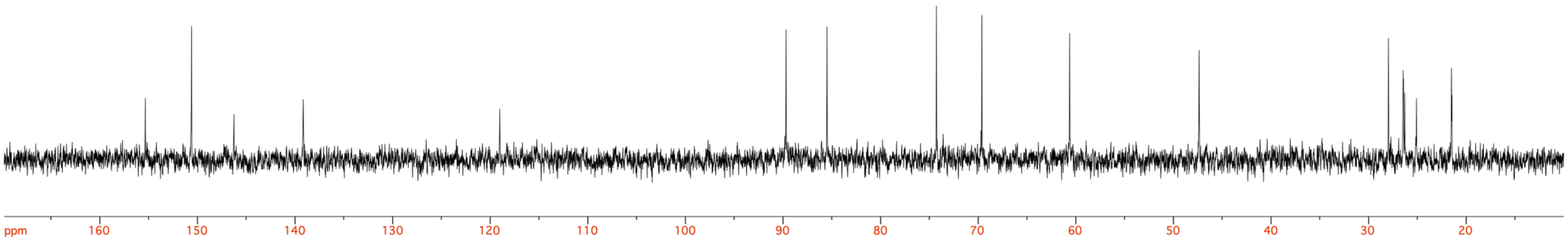

ppm
160
150
140
130
120
110
100
90
80
70
60
50
40
30
20

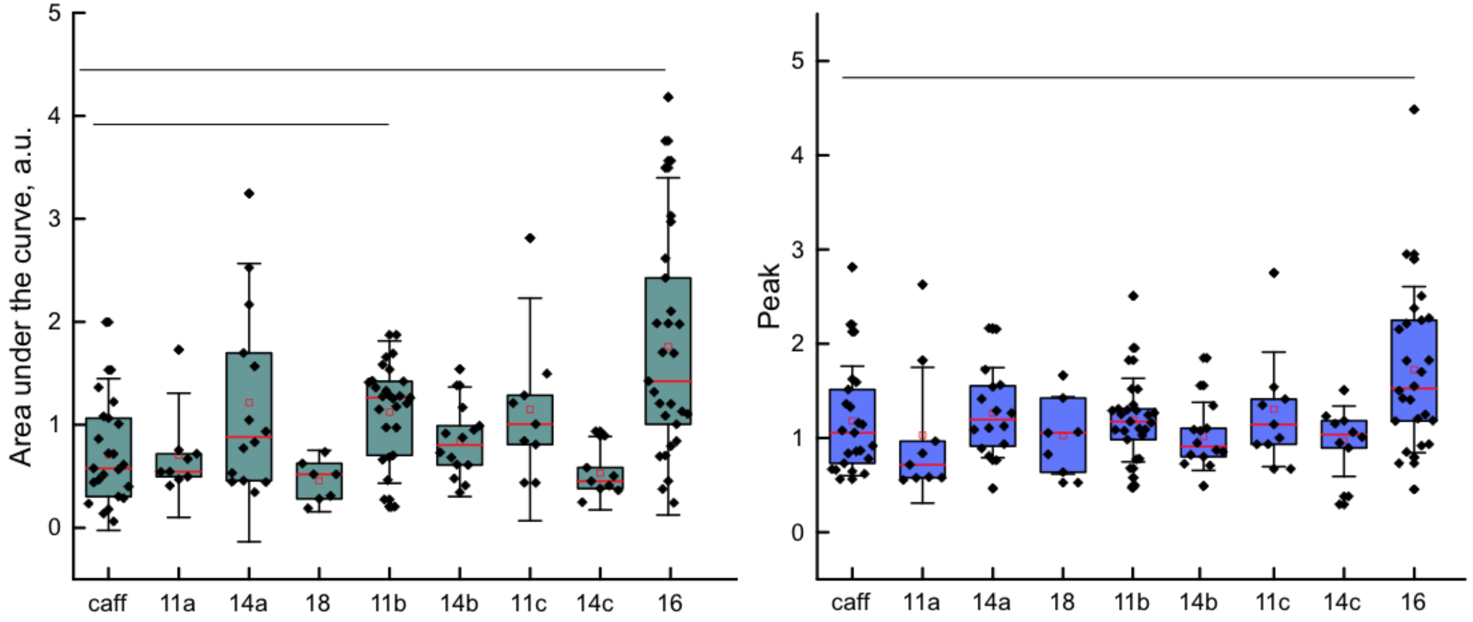

Figure 3. Average area (left panel) and peak values (right panel), above the baseline, in response to 20 mM caffeine addition, or to caffeine added 5 min after the addition of 1 μM of the indicated compound. Box size 25-75 percentile, red square: mean values, red bar: median, whiskers: standard deviation, each symbol represents an individual cell. Only cells responding to caffeine were included in this calculation. P 0.05
